# Supplementary material for: A genetic compensatory mechanism regulated by Jun and Mef2d modulates the expression of distinct class IIa Hdacs to ensure peripheral nerve myelination and repair
Source: eLife. 2022 Jan 25;11:e72917. doi: 10.7554/eLife.72917 (PMC8853665; doi:10.7554/eLife.72917)
Supplement: Source data 1. [file elife-72917-data1.docx]

**Figure 1**

Figure 1A – Compensation dKO mRNA % of control littermate

| **Gen** | **Age** | **Mean** | **SEM** | **n** | **Comparisons made**  **p-value** |
| --- | --- | --- | --- | --- | --- |
| *Hdac7* | P60 | 325.08 | 48.08 | 4 | control VS dKO  p=0.0034  ** |
| *Hdac9* |  | 122.22 | 13.26 | 7 | control VS dKO  p=0.1196  ns |

Unpaired t test

Figure 1C – TEM Nerve area (µ^2^)

| **Genotype** | **Age** | **Mean** | **SEM** | **n** | **Comparisons made**  **p-value** |
| --- | --- | --- | --- | --- | --- |
| control | P2 | 31506 | 1908 | 4 | control VS tKO P2  p=0.5234  ns |
| tKO |  | 29689 | 1659 | 3 |  |
| control | P8 | 62480 | 3070 | 4 | control VS tKO P8  p=0.9279  ns |
| tKO |  | 62166 | 1283 | 4 |  |
| control | P21 | 88087 | 3382 | 3 | control VS tKO P21  p=0.9009  ns |
| tKO |  | 89401 | 8131 | 4 |  |

Unpaired t test

Figure 1D – TEM Myelinated axons

| **Genotype** | **Age** | **Mean** | **SEM** | **n** | **Comparisons made**  **p-value** |
| --- | --- | --- | --- | --- | --- |
| control | P2 | 1160 | 29 | 4 | control VS tKO P2  p<0.0001  *** |
| tKO |  | 208 | 24 | 3 |  |
| control | P8 | 4325 | 129 | 4 | control VS tKO P8  p<0.0001  *** |
| tKO |  | 1487 | 179 | 4 |  |
| control | P21 | 5550 | 166 | 3 | control VS tKO P21  p=0.1412  ns |
| tKO |  | 5120 | 172 | 4 |  |

Unpaired t test

Figure 1E – TEM g-ratio

| **Genotype** | **Age** | **Mean** | **SEM** | **n** | **Comparisons made** | **p-value** |
| --- | --- | --- | --- | --- | --- | --- |
| control | P8 | 0.76 | 0.01 | 4 | control VS tKO P8  p=0.0045  ** | |
| tKO |  | 0.80 | 0.01 | 4 |  |  |
| control | P21 | 0.74 | 0.03 | 3 | control VS tKO P21  p=0.9633  ns | |
| tKO |  | 0.74 | 0.01 | 4 |  |  |

Unpaired t test

Figure 1F – TEM Unmyelinated axons in 1:1 relationship

| **Genotype** | **Age** | **Mean** | **SEM** | **n** | **Comparisons made** | **p-value** |
| --- | --- | --- | --- | --- | --- | --- |
| control | P2 | 971 | 67 | 4 | control VS tKO P2  p=0.6517  ns | |
| tKO |  | 920 | 84 | 3 |  |  |
| control | P8 | 628 | 21 | 4 | control VS tKO P8  p<0.0001  *** | |
| tKO |  | 3187 | 111 | 4 |  |  |
| control | P21 | 32 | 24 | 3 | control VS tKO P21  p=0.0559  ns | |
| tKO |  | 110 | 20 | 4 |  |  |

Unpaired t test

Figure 1G – TEM Total axons number in 1:1 relationship

| **Genotype** | **Age** | **Mean** | **SEM** | **n** | **Comparisons made**  **p-value** |
| --- | --- | --- | --- | --- | --- |
| control | P2 | 2131 | 95 | 4 | control VS tKO P2  p=0.0007  *** |
| tKO |  | 1128 | 90 | 3 |  |
| control | P8 | 4953 | 149 | 4 | control VS tKO P8  p=0.2899  ns |
| tKO |  | 4674 | 188 | 4 |  |
| control | P21 | 5557 | 167 | 3 | control VS tKO P21  p=0.2506  ns |
| tKO |  | 5230 | 177 | 4 |  |

Unpaired t test

Figure 1H – TEM Schwann cell nuclei

| **Genotype** | **Age** | **Mean** | **SEM** | **n** | **Comparisons made**  **p-value** |
| --- | --- | --- | --- | --- | --- |
| control | P2 | 340 | 8 | 4 | control VS tKO P2  p=0.0825  ns |
| tKO |  | 391 | 26 | 3 |  |
| control | P8 | 476 | 20 | 4 | control VS tKO P8  p=0.0002  *** |
| tKO |  | 823 | 37 | 4 |  |
| control | P21 | 337 | 32 | 3 | control VS tKO P21  p=0.0152  * |
| tKO |  | 503 | 31 | 4 |  |

Unpaired t test

Figure 1I – TEM Myelinating Schwann cell nuclei

| **Genotype** | **Age** | **Mean** | **SEM** | **n** | **Comparisons made**  **p-value** |
| --- | --- | --- | --- | --- | --- |
| control | P2 | 134 | 8 | 4 | control VS tKO P2  p<0.0001  *** |
| tKO |  | 22 | 1 | 3 |  |
| control | P8 | 309 | 11 | 4 | control VS tKO P8  p=0.0013  ** |
| tKO |  | 153 | 25 | 4 |  |
| control | P21 | 251 | 19 | 3 | control VS tKO P21  p=0.0780  ns |
| tKO |  | 310 | 18 | 4 |  |

Unpaired t test

Figure 1J – TEM % Axons

| **Genotype** | **Age** | **Mean** | **SEM** | **n** | **Comparisons made**  **p-value** |
| --- | --- | --- | --- | --- | --- |
| control | P2 | 54.6 | 1.1 | 4 | control VS tKO P2  p<0.0001  *** |
| tKO |  | 18.5 | 2.1 | 3 |  |
| control | P8 | 87.2 | 0.1 | 4 | control VS tKO P8  p<0.0001  *** |
| tKO |  | 31.6 | 2.9 | 4 |  |
| control | P21 | 99.9 | 0.0 | 3 | control VS tKO P21  p=0.0135  ** |
| tKO |  | 97.9 | 0.4 | 4 |  |

Unpaired t test

Figure 1K – qPCR P2 mRNA % of control littermate

| **Gen** | **Age** | **Mean** | **SEM** | **n** | **Comparisons made** | **p-value** |
| --- | --- | --- | --- | --- | --- | --- |
| *Jun* | P2 | 326.00 | 58.70 | 4 | control VS tKO P2 | p=0.0032  ** |
| *Runx2* |  | 457.14 | 53.36 | 5 | control VS tKO P2 | p=0.0002  *** |
| *Ngfr* |  | 276.60 | 25.51 | 5 | control VS tKO P2 | p=0.0001  *** |
| *Pou3f1* |  | 174.83 | 11.83 | 5 | control VS tKO P2 | p=0.002  *** |
| *Sox10* |  | 104.89 | 5.54 | 5 | control VS tKO P2 | p=0.4032  ns |
| *Krox-20* |  | 60.67 | 10.09 | 5 | control VS tKO P2 | p=0.0046  ** |
| *Prx* |  | 33.50 | 6.79 | 5 | control VS tKO P2 | p<0.0001  *** |
| *Mpz* |  | 31.93 | 4.78 | 5 | control VS tKO P2 | p<0.0001  *** |
| *Hmgcr* |  | 34.75 | 5.39 | 5 | control VS tKO P2 | p=<0.0001  *** |

Unpaired t test

Figure 1L – qPCR P8 mRNA % of control littermate

| **Gen** | **Age** | **Mean** | **SEM** | **n** | **Comparisons made** | **p-value** |
| --- | --- | --- | --- | --- | --- | --- |
| *Jun* | P8 | 356.04 | 27.54 | 4 | control VS tKO P8 | p <0.0001  *** |
| *Runx2* |  | 667.05 | 28.07 | 4 | control VS tKO P8 | p <0.0001  *** |
| *Ngf*r |  | 331.19 | 15.11 | 4 | control VS tKO P8 | p <0.0001  *** |
| *Pou3f1* |  | 307.62 | 60.77 | 4 | control VS tKO P8 | p =0.0142  * |
| *Sox10* |  | 133.39 | 9.11 | 4 | control VS tKO P8 | p =0.0105  * |
| *Krox-20* |  | 119.36 | 11.82 | 4 | control VS tKO P8 | p =0.1527  ns |
| *Prx* |  | 66.69 | 1.81 | 4 | control VS tKO P8 | p <0.0001  *** |
| *Mpz* |  | 64.32 | 4.80 | 4 | control VS tKO P8 | p =0.0003  *** |
| *Hmgcr* |  | 66.94 | 3.86 | 4 | control VS tKO P8 | p =0.0001  *** |

Unpaired t test

Figure 1M – qPCR P21 mRNA % of control littermate

| **Gen** | **Age** | **Mean** | **SEM** | **n** | **Comparisons made** | **p-value** |
| --- | --- | --- | --- | --- | --- | --- |
| *Jun* | P21 | 425.27 | 31.17 | 4 | control VS tKO P21 | p <0.0001  *** |
| *Runx2* |  | 911.14 | 217.06 | 4 | control VS tKO P21 | p =0.0097  ** |
| *Ngf*r |  | 656.21 | 82.33 | 4 | control VS tKO P21 | p =0.0005  *** |
| *Pou3f1* |  | 552.32 | 44.98 | 4 | control VS tKO P21 | p <0.0001  *** |
| *Sox10* |  | 142.76 | 18.68 | 4 | control VS tKO P21 | p =0.0620  ns |
| *Krox-20* |  | 103.68 | 2.76 | 4 | control VS tKO P21 | p =0.2304  ns |
| *Prx* |  | 76.28 | 3.06 | 4 | control VS tKO P21 | p =0.0002  *** |
| *Mpz* |  | 79.48 | 5.83 | 4 | control VS tKO P21 | p =0.0126  * |
| *Hmgcr* |  | 84.35 | 4.29 | 4 | control VS tKO P21 | p =0.0108  * |

Unpaired t test

Figure 1N - Western Blot P8.

| **Genotype** | **Protein** | **Age** | **Mean** | **SEM** | **n** | **Comparisons made**  **p-value** |
| --- | --- | --- | --- | --- | --- | --- |
| WT | JUN | P8 | 1.298 | 0.133 | 4 | WT VS control  p =0.8952  ns |
| control | JUN |  | 1.119 | 0.0708 | 4 | control VS tKO  p =0.0040  ** |
| tKO | JUN |  | 2.888 | 0.1901 | 4 | WT VS tKO  p =0.0077  ** |

One-way ANOVA tukey’s test P=0.028 **

| **Genotype** | **Protein** | **Age** | **Mean** | **SEM** | **n** | **Comparisons made**  **p-value** |
| --- | --- | --- | --- | --- | --- | --- |
| WT | KROX-20 | P8 | 0.919 | 0.1233 | 4 | WT VS control  p =0.9699  ns |
| control | KROX-20 |  | 0.979 | 0.0526 | 4 | control VS tKO  p =0.9537  ns |
| tKO | KROX-20 |  | 0.905 | 0.0780 | 4 | WT VS tKO  p =0.9981  ns |

One-way ANOVA tukey’s test P=0.9529 ns

| **Genotype** | **Protein** | **Age** | **Mean** | **SEM** | **n** | **Comparisons made**  **p-value** |
| --- | --- | --- | --- | --- | --- | --- |
| WT | MPZ | P8 | 1.1160 | 0.0556 | 4 | WT VS control  p =0.8717  ns |
| control | MPZ |  | 1.2065 | 0.0894 | 4 | control VS tKO  p =0.0128  * |
| tKO | MPZ |  | 0.5452 | 0.0323 | 4 | WT VS tKO  p =0.0276  * |

One-way ANOVA tukey’s test P=0.0103 **

| **Genotype** | **Protein** | **Age** | **Mean** | **SEM** | **n** | **Comparisons made**  **p-value** |
| --- | --- | --- | --- | --- | --- | --- |
| WT | MBP | P8 | 0.9192 | 0.0258 | 4 | WT VS control  p =0.1405  ns |
| control | MBP |  | 1.308 | 0.0998 | 4 | control VS tKO  p =0.0115  * |
| tKO | MBP |  | 0.6205 | 0.0447 | 4 | WT VS tKO  p =0.2840  ns |

One-way ANOVA tukey’s test P=0.0143*

**Figure 2**

Figure 2C – qPCR mRNA level relative to 18S control and tKO at P60

| **Genotype** | **Gen** | **Mean** | **SEM** | **n** | **Comparisons made**  **p-value** |
| --- | --- | --- | --- | --- | --- |
| control | *Jun* | 0.72x10^-4^ | 0.05 x10^-4^ | 5 | control VS tKO  p=0.0027  ** |
| tKO |  | 1.88 x10^-4^ | 0.19 x10^-4^ | 5 |  |

Unpaired t test with Welch´s correlation

| **Genotype** | **Gen** | **Mean** | **SEM** | **n** | **Comparisons made**  **p-value** |
| --- | --- | --- | --- | --- | --- |
| control | *Mpz* | 2.13 x10^-2^ | 0.16 x10^-4^ | 5 | control VS tKO  p=0.0270  * |
| tKO |  | 1.57 x10^-2^ | 0.13 x10^-4^ | 5 |  |

Unpaired t test

Figure 2D – Western Blot UI WT. control and tKO normalize to WT at P60

| **Genotype** | **Protein** | **Mean** | **SEM** | **n** | **Comparisons made** | **p-value** |
| --- | --- | --- | --- | --- | --- | --- |
| WT | JUN | 1 | 0.11 | 6 | WT VS control | p=0.9568  ns |
| control |  | 1.05 | 0.04 | 7 | WT VS tKO | p=0.0002  *** |
| tKO |  | 2.04 | 0.22 | 6 | control VS tKO | p=0.0003  *** |

One-way ANOVA tukey’s test P<0.0001 ***

| **Genotype** | **Protein** | **Mean** | **SEM** | **n** | **Comparisons made** | **p-value** |
| --- | --- | --- | --- | --- | --- | --- |
| WT | MPZ | 1 | 0.02 | 7 | WT VS control | p=0.9725  ns |
| control |  | 0.98 | 0.06 | 7 | WT VS tKO | p=0.0663  ns |
| tKO |  | 0.81 | 0.07 | 7 | control VS tKO | p=0.1014  ns |

One-way ANOVA tukey’s test P=0.0506 ns

Figure 2E – Axons per Remak Bundle at P60

| **Genotype** | **# axons per Remak Bundle** | **Mean** | **SEM** | **n** | **Comparisons made**  **p-value** |
| --- | --- | --- | --- | --- | --- |
| control | 2-5 | 23 | 1 | 4 | control VS tKO  p=0.0187  * |
| tKO |  | 33 | 3 | 4 |  |
| control | 6-10 | 21 | 1 | 4 | control VS tKO  p=0.1357  ns |
| tKO |  | 17 | 2 | 4 |  |
| control | 11-15 | 23 | 1 | 4 | control VS tKO  p=0.0010  ** |
| tKO |  | 14 | 1 | 4 |  |
| control | 16-20 | 12 | 2 | 4 | control VS tKO  p=0.1189  ns |
| tKO |  | 8 | 1 | 4 |  |
| control | 21-30 | 12 | 1 | 4 | control VS tKO  p=0.0949  ns |
| tKO |  | 9 | 1 | 4 |  |
| control | +30 | 9 | 2 | 4 | control VS tKO  p=0.1055  ns |
| tKO |  | 19 | 5 | 4 |  |

Mixed Model ANOVA with Bonferroni post-hoc test (P=0.0003)

| **Genotype** | **# axons per pocket** | **Mean** | **SEM** | **n** | **Comparisons made**  **p-value** |
| --- | --- | --- | --- | --- | --- |
| control | single | 82 | 1 | 4 | control VS tKO  p=0.0013  ** |
| tKO |  | 54 | 5 | 4 |  |
| control | 2-5 | 16 | 1 | 4 | control VS tKO  p=0.0003  *** |
| tKO |  | 30 | 2 | 4 |  |
| control | 6-10 | 2 | 0 | 4 | control VS tKO  p=0.0125  ** |
| tKO |  | 7 | 1 | 4 |  |
| control | 11-15 | 0 | 0 | 4 | control VS tKO  p=0.0134  * |
| tKO |  | 3 | 1 | 4 |  |
| control | 16-20 | 0 | 0 | 4 | control VS tKO  p=0.0171  * |
| tKO |  | 2 | 1 | 4 |  |
| control | 21-30 | 0 | 0 | 4 | control VS tKO  p=0.0050  ** |
| tKO |  | 2 | 1 | 4 |  |
| control | +30 | 0 | 0 | 4 | control VS tKO  p=0.2154  ns |
| tKO |  | 2 | 1 | 4 |  |

Mixed Model ANOVA with Bonferroni post-hoc test (P<0.0001)

| **Genotype** | **Axon diameter** | **Mean** | **SEM** | **n** | **Comparisons made**  **p-value** |
| --- | --- | --- | --- | --- | --- |
| control | <0.2µm | 0.24 | 0.19 | 4 | control VS tKO  P= 0.3  ns |
| tKO |  | 0.54 | 0.15 | 4 |  |
| control | 0.2-0.4 µm | 13.50 | 0.27 | 4 | control VS tKO  p = 0.15  ns |
| tKO |  | 11.08 | 1.45 | 4 |  |
| control | 0.4-0.6 µm | 50.63 | 1.65 | 4 | control VS tKO  p = 0.8  ns |
| tKO |  | 49.72 | 3.19 | 4 |  |
| control | 0.6-0.8 µm | 29.83 | 0.89 | 4 | control VS tKO  p = 0.58  ns |
| tKO |  | 31.55 | 2.76 | 4 |  |
| control | 0.8-1 µm | 4.42 | 0.93 | 4 | control VS tKO  p = 0.36  ns |
| tKO |  | 6.13 | 1.47 | 4 |  |
| control | >1 µm | 1.37 | 0.36 | 4 | control VS tKO  p = 0.46  ns |
| tKO |  | 0.98 | 0.34 | 4 |  |

Mixed Model ANOVA with Bonferroni post-hoc test (P=0.56)

**Figure 3**

Figure 3A – qPCR *Tyrp1* mRNA level relative to 18S at P60

| **Genotype** | **Gen** | **Mean** | **SEM** | **n** | **Comparisons made**  **p-value** |
| --- | --- | --- | --- | --- | --- |
| control | *Tyrp1* | 0.18x10^-6^ | 0.03x10^-6^ | 5 | control VS cKO4  p=0.0505  ns |
| cKO4 |  | 0.67x10^-6^ | 0.18x10^-6^ | 5 |  |

Unpaired t test with Welch´s correlation

| **Genotype** | **Gen** | **Mean** | **SEM** | **n** | **Comparisons made**  **p-value** |
| --- | --- | --- | --- | --- | --- |
| control | *Tyrp1* | 0.27x10^-6^ | 0.04x10^-6^ | 4 | control VS cKO7  p=0.0705  ns |
| cKO7 |  | 0.62x10^-6^ | 0.16x10^-6^ | 4 |  |

Unpaired t test

| **Genotype** | **Gen** | **Mean** | **SEM** | **n** | **Comparisons made**  **p-value** |
| --- | --- | --- | --- | --- | --- |
| control | *Tyrp1* | 0.11x10^-6^ | 0.03x10^-6^ | 4 | control VS dKO  p=0.0547  ns |
| dKO |  | 2x10^-6^ | 0.61x10^-6^ | 4 |  |

Unpaired t test with Welch´s correlation

| **Genotype** | **Gen** | **Mean** | **SEM** | **n** | **Comparisons made**  **p-value** |
| --- | --- | --- | --- | --- | --- |
| control | *Tyrp1* | 0.31x10^-6^ | 0.05x10^-6^ | 5 | control VS tKO  p=0.0092  ** |
| tKO |  | 3.35x10^-4^ | 0.71x10^-4^ | 5 |  |

Unpaired t test with Welch´s correlation

Figure 3B – qPCR *Mcam* mRNA level relative to 18S at P60

| **Genotype** | **Gen** | **Mean** | **SEM** | **n** | **Comparisons made**  **p-value** |
| --- | --- | --- | --- | --- | --- |
| control | *Mcam* | 1.3x10^-4^ | 0.07x10^-4^ | 5 | control VS cKO4  p=0.0086  ** |
| cKO4 |  | 1.69x10^-4^ | 0.09x10^-4^ | 5 |  |

Unpaired t test

| **Genotype** | **Gen** | **Mean** | **SEM** | **n** | **Comparisons made**  **p-value** |
| --- | --- | --- | --- | --- | --- |
| control | *Mcam* | 1.15x10^-4^ | 0.17x10^-4^ | 4 | control VS cKO7  p=0.0048  ** |
| cKO7 |  | 2.11x10^-4^ | 0.14x10^-4^ | 4 |  |

Unpaired t test

| **Genotype** | **Gen** | **Mean** | **SEM** | **n** | **Comparisons made**  **p-value** |
| --- | --- | --- | --- | --- | --- |
| control | *Mcam* | 0.24x10^-4^ | 0.04x10^-4^ | 4 | control VS dKO  p=0.2589  ns |
| dKO |  | 0.34x10^-4^ | 0.07x10^-4^ | 4 |  |

Unpaired t test with Welch´s correlation

| **Genotype** | **Gen** | **Mean** | **SEM** | **n** | **Comparisons made**  **p-value** |
| --- | --- | --- | --- | --- | --- |
| control | *Mcam* | 1.44x10^-4^ | 0.06x10^-4^ | 5 | control VS tKO  p=0.0016  ** |
| tKO |  | 7.39x10^-4^ | 0.79x10^-4^ | 5 |  |

Unpaired t test with Welch´s correlation

Figure 3C – qPCR *Mitf* mRNA level relative to 18S at P60

| **Genotype** | **Gen** | **Mean** | **SEM** | **n** | **Comparisons made**  **p-value** |
| --- | --- | --- | --- | --- | --- |
| control | *Mitf* | 0.29x10^-5^ | 0.03x10^-5^ | 5 | control VS cKO4  p=0.0621  ns |
| cKO4 |  | 0.03x10^-5^ | 0.01x10^-5^ | 5 |  |

Unpaired t test

| **Genotype** | **Gen** | **Mean** | **SEM** | **n** | **Comparisons made**  **p-value** |
| --- | --- | --- | --- | --- | --- |
| control | *Mitf* | 0.43x10^-5^ | 0.05x10^-5^ | 4 | control VS cKO7  p=0.1578  ns |
| cKO7 |  | 0.52x10^-5^ | 0.03x10^-5^ | 4 |  |

Unpaired t test

| **Genotype** | **Gen** | **Mean** | **SEM** | **n** | **Comparisons made**  **p-value** |
| --- | --- | --- | --- | --- | --- |
| control | *Mitf* | 0.12x10^-5^ | 0.01x10^-5^ | 4 | control VS dKO  p=0.9338  ns |
| dKO |  | 0.13x10^-5^ | 0.01x10^-5^ | 4 |  |

Unpaired t test

| **Genotype** | **Gen** | **Mean** | **SEM** | **n** | **Comparisons made**  **p-value** |
| --- | --- | --- | --- | --- | --- |
| control | *Mitf* | 0.5x10^-5^ | 0.02x10^-5^ | 5 | control VS tKO  p=0.0128  * |
| tKO |  | 0.87x10^-5^ | 0.09x10^-5^ | 5 |  |

Unpaired t test with Welch´s correlation

Figure 3D – qPCR *Ednrb* mRNA level relative to 18S at P60

| **Genotype** | **Gen** | **Mean** | **SEM** | **n** | **Comparisons made**  **p-value** |
| --- | --- | --- | --- | --- | --- |
| control | *Ednrb* | 0.74x10^-5^ | 0.05x10^-5^ | 5 | control VS cKO4  p=0.4558  ns |
| cKO4 |  | 0.68x10^-5^ | 0.07x10^-5^ | 5 |  |

Unpaired t test

| **Genotype** | **Gen** | **Mean** | **SEM** | **n** | **Comparisons made**  **p-value** |
| --- | --- | --- | --- | --- | --- |
| control | *Ednrb* | 0.38x10^-5^ | 0.04x10^-5^ | 4 | control VS cKO7  p=0.0592  ns |
| cKO7 |  | 0.55x10^-5^ | 0.06x10^-5^ | 4 |  |

Unpaired t test

| **Genotype** | **Gen** | **Mean** | **SEM** | **n** | **Comparisons made**  **p-value** |
| --- | --- | --- | --- | --- | --- |
| control | *Ednrb* | 0.23x10^-5^ | 0.03x10^-5^ | 4 | control VS dKO  p=0.3935  ns |
| dKO |  | 0.2x10^-5^ | 0.03x10^-5^ | 4 |  |

Unpaired t test

| **Genotype** | **Gen** | **Mean** | **SEM** | **n** | **Comparisons made**  **p-value** |
| --- | --- | --- | --- | --- | --- |
| control | *Ednrb* | 1.04x10^-5^ | 0.09x10^-5^ | 5 | control VS tKO  p=0.0032  ** |
| tKO |  | 4.23x10^-5^ | 0.52x10^-5^ | 5 |  |

Unpaired t test with Welch´s correlation

Figure 3E - MCAM Western Blot data at P60.

| **Genotype** | **Time** | **Mean** | **SEM** | **n** | **Comparisons made**  **p-value** |
| --- | --- | --- | --- | --- | --- |
| WT | UI | 1 | 0.08 | 8 | WT VS control  p=0.9923  ns |
| control |  | 1.30 | 0.13 | 4 | control VS tKO  p=0.0012  ** |
| tKO |  | 9.93 | 1.75 | 8 | WT VS tKO  p=0.0087  ** |

One-way ANOVA tukey’s test P=0009 ***

Figure 3F - NGFR Western Blot data at P60.

| **Genotype** | **Time** | **Mean** | **SEM** | **n** | **Comparisons made**  **p-value** |
| --- | --- | --- | --- | --- | --- |
| WT | UI | 1.000 | 0.05485 | 8 | WT VS control  p=0.9998  ns |
| control |  | 1.005 | 0.09163 | 4 | control VS tKO  p=0.0012  ** |
| tKO |  | 2.161 | 0.28702 | 8 | WT VS tKO  p=0.0070  ** |

One-way ANOVA tukey’s test P=0003 ***

**Figure 4**

Figure 4B – TEM Nerve area (µ^2^)

| **Genotype** | **Time** | **Mean** | **SEM** | **n** | **Comparisons made**  **p-value** |
| --- | --- | --- | --- | --- | --- |
| control | UI | 157184 | 5744 | 5 | control VS dKO UI  p=0.8043  ns |
| dKO |  | 158703 | 2640 | 6 |  |
| control | 10dpi | 197245 | 15549 | 4 | control VS dKO 10dpi  p=0.1953  ns |
| dKO |  | 216999 | 5324 | 6 |  |
| control | 20dpi | 191436 | 8517 | 5 | control VS dKO 20dpi  p=0.5590  ns |
| dKO |  | 186199 | 3546 | 6 |  |
| control | 30dpi | 150122 | 4234 | 4 | control VS dKO 30dpi  p=0.0594  ns |
| dKO |  | 171300 | 8526 | 3 |  |

Unpaired t test

Figure 4C – TEM Myelinated axons

| **Genotype** | **Time** | **Mean** | **SEM** | **n** | **Comparisons made**  **p-value** |
| --- | --- | --- | --- | --- | --- |
| control | UI | 5764 | 67 | 5 | control VS dKO UI  p=0.1906  ns |
| dKO |  | 5518 | 147 | 6 |  |
| control | 10dpi | 1889 | 330 | 4 | control VS dKO 10dpi  p=0.0005  *** |
| dKO |  | 388 | 55 | 6 |  |
| control | 20dpi | 5437 | 258 | 5 | control VS dKO 20dpi  p=0.1586  ns |
| dKO |  | 5036 | 107 | 6 |  |
| control | 30dpi | 5614 | 248 | 4 | control VS dKO 30dpi  p=0.6105  ns |
| dKO |  | 5412 | 274 | 3 |  |

Unpaired t test

Figure 4D – TEM g-ratio

| **Genotype** | **Time** | **Mean** | **SEM** | **n** | **Comparisons made**  **p-value** |
| --- | --- | --- | --- | --- | --- |
| control | UI | 0.69 | 0.007 | 5 | control VS dKO UI  p=0.5028  ns |
| dKO |  | 0.684 | 0.006 | 6 |  |
| control | 10dpi | 0.934 | 0.015 | 4 | control VS dKO 10dpi  pP=0.0020  ** |
| dKO |  | 0.989 | 0.003 | 6 |  |
| control | 20dpi | 0.767 | 0.003 | 5 | control VS dKO 20dpi  p=0.0428  * |
| dKO |  | 0.776 | 0.003 | 6 |  |
| control | 30dpi | 0.758 | 0.001 | 4 | control VS dKO 30dpi  p=0.4920  ns |
| dKO |  | 0.754 | 0.006 | 3 |  |

Unpaired t test

Figure 4E – TEM Unmyelinated axons number >1.5µm

| **Genotype** | **Time** | **Mean** | **SEM** | **n** | **Comparisons made**  **p-value** |
| --- | --- | --- | --- | --- | --- |
| control | UI | 46 | 14 | 5 | control VS dKO UI  p=0.5173  ns |
| dKO |  | 33 | 14 | 6 |  |
| control | 10dpi | 1512 | 119 | 4 | control VS dKO 10dpi  p=0.0007  *** |
| dKO |  | 2969 | 203 | 6 |  |
| control | 20dpi | 88 | 14 | 5 | control VS dKO 20dpi  p=0.0016  ** |
| dKO |  | 224 | 25 | 6 |  |
| control | 30dpi | 43 | 11 | 4 | control VS dKO 30dpi  p=0.1218  ns |
| dKO |  | 81 | 18 | 3 |  |

Unpaired t test

Figure 4F – TEM Unmyelinated >1.5µm axons in 1:1 relationship

| **Genotype** | **Time** | **Mean** | **SEM** | **n** | **Comparisons made**  **p-value** |
| --- | --- | --- | --- | --- | --- |
| control | UI | 4 | 1 | 5 | control VS dKO UI  p=0.5983  ns |
| dKO |  | 5 | 2 | 6 |  |
| control | 10dpi | 1158 | 56 | 4 | control VS dKO 10dpi  p=0.0011  ** |
| dKO |  | 2148 | 155 | 6 |  |
| control | 20dpi | 68 | 12 | 5 | control VS dKO 20dpi  p=0.0020  ** |
| dKO |  | 175 | 20 | 6 |  |
| control | 30dpi | 22 | 5 | 4 | control VS dKO 30dpi  p=0.0432  * |
| dKO |  | 63 | 17 | 3 |  |

Unpaired t test

Figure 4G – TEM Total axon number >1.5µm

| **Genotype** | **Time** | **Mean** | **SEM** | **n** | **Comparisons made**  **p-value** |
| --- | --- | --- | --- | --- | --- |
| control | UI | 5810 | 78 | 5 | control VS dKO UI  p=0.1562  ns |
| dKO |  | 5551 | 137 | 6 |  |
| control | 10dpi | 3401 | 370 | 4 | control VS dKO 10dpi  p=0.9096  ns |
| dKO |  | 3358 | 180 | 6 |  |
| control | 20dpi | 5524 | 264 | 5 | control VS dKO 20dpi  p=0.3490  ns |
| dKO |  | 5260 | 110 | 6 |  |
| control | 30dpi | 5657 | 239 | 4 | control VS dKO 30dpi  p=0.6655  ns |
| dKO |  | 5493 | 262 | 3 |  |

Unpaired t test

Figure 4H – TEM Total axon number >1.5µm axons in 1:1 relationship

| **Genotype** | **Time** | **Mean** | **SEM** | **n** | **Comparisons made**  **p-value** |
| --- | --- | --- | --- | --- | --- |
| control | UI | 5767 | 68 | 5 | control VS dKO UI  p=0.1931  ns |
| dKO |  | 5523 | 147 | 6 |  |
| control | 10dpi | 3047 | 350 | 4 | control VS dKO 10dpi  p=0.1688  ns |
| dKO |  | 2536 | 155 | 6 |  |
| control | 20dpi | 5504 | 265 | 5 | control VS dKO 20dpi  p=0.2943  ns |
| dKO |  | 5210 | 101 | 6 |  |
| control | 30dpi | 5636 | 245 | 4 | control VS dKO 30dpi  p=0.6821  ns |
| dKO |  | 5475 | 277 | 3 |  |

Unpaired t test

Figure 4I – TEM Schwann cell nuclei

| **Genotype** | **Time** | **Mean** | **SEM** | **n** | **Comparisons made**  **p-value** |
| --- | --- | --- | --- | --- | --- |
| control | UI | 243 | 9 | 5 | control VS dKO UI  p=0.3925  ns |
| dKO |  | 262 | 18 | 6 |  |
| control | 10dpi | 615 | 50 | 4 | control VS dKO 10dpi  p=0.0543  ns |
| dKO |  | 724 | 22 | 6 |  |
| control | 20dpi | 630 | 53 | 5 | control VS dKO 20dpi  p=0.0041  ** |
| dKO |  | 861 | 34 | 6 |  |
| control | 30dpi | 602 | 47 | 4 | control VS dKO 30dpi  p=0.4146  ns |
| dKO |  | 654 | 22 | 3 |  |

Unpaired t test

Figure 4J – TEM Myelinating Schwann cell nuclei

| **Genotype** | **Time** | **Mean** | **SEM** | **n** | **Comparisons made**  **p-value** |
| --- | --- | --- | --- | --- | --- |
| control | UI | 176 | 9 | 5 | control VS dKO UI  p=0.1239  ns |
| dKO |  | 203 | 12 | 6 |  |
| control | 10dpi | 164 | 37 | 4 | control VS dKO 10dpi  p=0.0032  ** |
| dKO |  | 35 | 8 | 6 |  |
| control | 20dpi | 267 | 22 | 5 | control VS dKO 20dpi  p=0.3393  ns |
| dKO |  | 292 | 14 | 6 |  |
| control | 30dpi | 328 | 24 | 4 | control VS dKO 30dpi  p=0.1811  ns |
| dKO |  | 275 | 22 | 3 |  |

Unpaired t test

Figure 4K – % Axons >1.5µm

| **Genotype** | **Time** | **Mean** | **SEM** | **n** | **Comparisons made**  **p-value** |
| --- | --- | --- | --- | --- | --- |
| control | UI | 99.9 | 0.0 | 5 | control VS dKO UI  p=0.4759  ns |
| dKO |  | 99.9 | 0.0 | 6 |  |
| control | 10dpi | 60.4 | 4.8 | 4 | control VS dKO 10dpi  p<0.0001  *** |
| dKO |  | 15.5 | 2.3 | 6 |  |
| control | 20dpi | 98.8 | 0.2 | 5 | control VS dKO 20dpi  p=0.0016  ** |
| dKO |  | 96.6 | 0.4 | 6 |  |
| control | 30dpi | 99.6 | 0.1 | 4 | control VS dKO 30dpi  p=0.0482  * |
| dKO |  | 98.9 | 0.3 | 3 |  |

Unpaired t test

Figure 4L – qPCR 10 dpi dKO mRNA fold change injured vs uninjured

| **Genotype** | **gen** | **Mean** | **SEM** | **n** | **Comparisons made**  **p-value** |
| --- | --- | --- | --- | --- | --- |
| control | *Jun* | 2.74 | 0.20 | 5 | control VS dKO  p=0.0056  ** |
| dKO |  | 4.15 | 0.30 | 7 |  |

Unpaired t test

| **Genotype** | **gen** | **Mean** | **SEM** | **n** | **Comparisons made**  **p-value** |
| --- | --- | --- | --- | --- | --- |
| control | *Runx2* | 14.66 | 2.33 | 7 | control VS dKO  p=0.3189  ns |
| dKO |  | 19.03 | 3.63 | 6 |  |

Unpaired t test

| **Genotype** | **gen** | **Mean** | **SEM** | **n** | **Comparisons made**  **p-value** |
| --- | --- | --- | --- | --- | --- |
| control | *Gdnf* | 30.90 | 2.34 | 8 | control VS dKO  p=0.0025  ** |
| dKO |  | 57.05 | 5.89 | 8 |  |

Unpaired t test with Welch´s correlation

| **Genotype** | **gen** | **Mean** | **SEM** | **n** | **Comparisons made**  **p-value** |
| --- | --- | --- | --- | --- | --- |
| control | *Bdnf* | 12.74 | 2.71 | 8 | control VS dKO  p=0.0010  *** |
| dKO |  | 33.12 | 4.11 | 8 |  |

Unpaired t test

| **Genotype** | **gen** | **Mean** | **SEM** | **n** | **Comparisons made**  **p-value** |
| --- | --- | --- | --- | --- | --- |
| control | *Olig1* | 165.15 | 22.29 | 8 | control VS dKO  p=0.0084  ** |
| dKO |  | 263.54 | 23.12 | 8 |  |

Unpaired t test

Figure 4M – qPCR 10 dpi dKO mRNA fold change injured vs uninjured

| **Genotype** | **gen** | **Mean** | **SEM** | **n** | **Comparisons made**  **p-value** |
| --- | --- | --- | --- | --- | --- |
| control | *Krox-20* | 0.43 | 0.06 | 8 | control VS dKO  p=0.0068  ** |
| dKO |  | 0.20 | 0.02 | 8 |  |

Unpaired t test with Welch´s correlation

| **Genotype** | **gen** | **Mean** | **SEM** | **n** | **Comparisons made**  **p-value** |
| --- | --- | --- | --- | --- | --- |
| control | *Pou3f1* | 7.89 | 0.82 | 8 | control VS dKO  p=0.0534  ns |
| dKO |  | 5.94 | 0.29 | 7 |  |

Unpaired t test with Welch´s correlation

| **Genotype** | **gen** | **Mean** | **SEM** | **n** | **Comparisons made**  **p-value** |
| --- | --- | --- | --- | --- | --- |
| control | *Prx* | 0.22 | 0.02 | 8 | control VS dKO  p=0.0012  ** |
| dKO |  | 0.10 | 0.01 | 8 |  |

Unpaired t test with Welch´s correlation

| **Genotype** | **gen** | **Mean** | **SEM** | **n** | **Comparisons made**  **p-value** |
| --- | --- | --- | --- | --- | --- |
| control | *Mpz* | 0.22 | 0.03 | 8 | control VS dKO  p=0.0054  *** |
| dKO |  | 0.08 | 0.01 | 8 |  |

Unpaired t test with Welch´s correlation

| **Genotype** | **gen** | **Mean** | **SEM** | **n** | **Comparisons made**  **p-value** |
| --- | --- | --- | --- | --- | --- |
| control | *Mbp* | 0.15 | 0.03 | 8 | control VS dKO  p=0.0121  * |
| dKO |  | 0.05 | 0.01 | 6 |  |

Unpaired t test with Welch´s correlation

Figure 4N – Western Blot JUN. KROX-20 y MPZ 10 y 21 dpi

| **Genotype** | **JUN** | **Mean** | **SEM** | **n** | **Comparisons made**  **p-value** |
| --- | --- | --- | --- | --- | --- |
| UI | UI | 0.20 | 0.02 | 8 | N / A |
| WT | 10dpi | 1 | 0.06 | 7 | WT VS KO5 10dpi  p=0.1459  ns |
| KO5 |  | 1.17 | 0.09 | 7 |  |
| control | 10dpi | 1.09 | 0.13 | 7 | control VS dKO 10dpi  p=0.0117  * |
| dKO |  | 1.72 | 0.17 | 7 |  |
| control | 21dpi | 0.92 | 0.10 | 9 | control VS dKO 21dpi  p=0.0501  ns |
| dKO |  | 1.32 | 0.16 | 9 |  |

Unpaired t test

| **Genotype** | **KROX-20** | **Mean** | **SEM** | **n** | **Comparisons made**  **p-value** |
| --- | --- | --- | --- | --- | --- |
| UI | UI | 0.57 | 0.05 | 8 | N / A |
| WT | 10dpi | 1 | 0.04 | 7 | WT VS KO5 10dpi  p=0.5275  ns |
| KO5 |  | 0.95 | 0.07 | 7 |  |
| control | 10dpi | 1.11 | 0.09 | 7 | control VS dKO 10dpi  p=0.1500  ns |
| dKO |  | 0.94 | 0.06 | 7 |  |
| control | 21dpi | 1.11 | 0.06 | 9 | control VS dKO 21dpi  p=0.7287  ns |
| dKO |  | 1.08 | 0.06 | 9 |  |

Unpaired t test

| **Genotype** | **MPZ** | **Mean** | **SEM** | **n** | **Comparisons made**  **p-value** |
| --- | --- | --- | --- | --- | --- |
| UI | UI | 1 | 0.06 | 8 | N / A |
| WT | 10dpi | 0.13 | 0.01 | 7 | WT VS KO5 10dpi  p=0.5761  ns |
| KO5 |  | 0.13 | 0.01 | 7 |  |
| control | 10dpi | 0.15 | 0.01 | 7 | control VS dKO 10dpi  p=0.1683  ns |
| dKO |  | 0.13 | 0.01 | 7 |  |
| control | 21dpi | 0.40 | 0.02 | 9 | control VS dKO 21dpi  p=0.0204  * |
| dKO |  | 0.32 | 0.02 | 9 |  |

Unpaired t test

**Figure 5**

Figure 5A – Number of intact myelin sheaths per nerve after 4 day cut tKO

| **Genotype** | **Time** | **Mean** | **SEM** | **n** | **Comparisons made**  **p-value** |
| --- | --- | --- | --- | --- | --- |
| control | 4d cut | 274 | 8 | 4 | control VS tKO 4d cut  p<0.0001  *** |
| tKO |  | 150 | 7 | 5 |  |

Unpaired t test

Figure 5B – Western Blot 4 day cut tKO

| **Genotype** | **Time** | **Mean** | **SEM** | **n** | **Comparisons made**  **p-value** |
| --- | --- | --- | --- | --- | --- |
| control | JUN | 1 | 0.07 | 5 | control VS tKO 4d cut  p=0.0089  ** |
| tKO |  | 1.69 | 0.19 | 7 |  |
| control | MPZ | 1 | 0.04 | 5 | control VS tKO 4d cut  p=0.0003  *** |
| tKO |  | 0.64 | 0.05 | 7 |  |

Unpaired t test with Welch´s and t test

Figure 5C – Western Blot 7 day cut tKO

| **Genotype** | **Time** | **Mean** | **SEM** | **n** | **Comparisons made**  **p-value** |
| --- | --- | --- | --- | --- | --- |
| control | JUN | 1 | 0.10 | 4 | control VS tKO 7d cut  p=0.2455  ns |
| tKO |  | 1.31 | 0.24 | 3 |  |
| control | MPZ | 1 | 0.12 | 4 | control VS tKO 7d cut  p=0.0124  * |
| tKO |  | 0.43 | 0.06 | 3 |  |

Unpaired t test

Figure 5E – TEM Nerve area (µ^2^)

| **Genotype** | **Time** | **Mean** | **SEM** | **n** | **Comparisons made**  **p-value** |
| --- | --- | --- | --- | --- | --- |
| control | UI | 144945 | 3525 | 4 | control VS tKO UI  p=0.8973  ns |
| tKO |  | 143975 | 6289 | 4 |  |
| control | 10dpi | 172795 | 4233 | 4 | control VS tKO 10dpi  p=0.4559  ns |
| tKO |  | 181092 | 9511 | 4 |  |
| control | 20dpi | 185383 | 11910 | 5 | control VS tKO 20dpi  p=0.6020  ns |
| tKO |  | 194680 | 12303 | 5 |  |
| control | 30dpi | 177410 | 4592 | 5 | control VS tKO 30dpi  p=0.0002  *** |
| tKO |  | 224649 | 5800 | 5 |  |
| control | 60dpi | 174833 | 7817 | 5 | control VS tKO 60dpi  p=0.0008  *** |
| tKO |  | 227951 | 6391 | 5 |  |

Unpaired t test

Figure 5F – TEM Myelinated axons

| **Genotype** | **Time** | **Mean** | **SEM** | **n** | **Comparisons made**  **p-value** |
| --- | --- | --- | --- | --- | --- |
| control | UI | 6382 | 90 | 4 | control VS tKO UI  p=0.1160  ns |
| tKO |  | 6021 | 175 | 4 |  |
| control | 10dpi | 2383 | 112 | 4 | control VS tKO 10dpi  p<0.0001  *** |
| tKO |  | 0 | 0 | 4 |  |
| control | 20dpi | 5525 | 222 | 5 | control VS tKO 20dpi  v<0.0001  *** |
| tKO |  | 606 | 200 | 5 |  |
| control | 30dpi | 6003 | 125 | 5 | control VS tKO 30dpi  p<0.0001  *** |
| tKO |  | 2659 | 323 | 5 |  |
| control | 60dpi | 6689 | 212 | 5 | control VS tKO 60dpi  p=0.4908  ns |
| tKO |  | 6458 | 240 | 5 |  |

Unpaired t test

Figure 5G – TEM g-ratio

| **Genotype** | **Time** | **Mean** | **SEM** | **n** | **Comparisons made**  **p-value** |
| --- | --- | --- | --- | --- | --- |
| control | UI | 0.71 | 0.01 | 4 | control VS tKO UI  p=0.5155  ns |
| tKO |  | 0.70 | 0.007 | 4 |  |
| control | 10dpi | 0.92 | 0.003 | 4 | control VS tKO 10dpi  p<0.0001  *** |
| tKO |  | 1 | 0 | 4 |  |
| control | 20dpi | 0.77 | 0.006 | 5 | control VS tKO 20dpi  p=0.0023  ** |
| tKO |  | 0.94 | 0.027 | 5 |  |
| control | 30dpi | 0.75 | 0.002 | 5 | control VS tKO 30dpi  p=0.0009  *** |
| tKO |  | 0.82 | 0.008 | 5 |  |
| control | 60dpi | 0.75 | 0.005 | 4 | control VS tKO 60dpi  p=0.1945  ns |
| tKO |  | 0.77 | 0.011 | 4 |  |

Unpaired t test & Unpaired t test with Welch´s correlation

Figure 5H – TEM Unmyelinated axons number >1.5µm

| **Genotype** | **Time** | **Mean** | **SEM** | **n** | **Comparisons made**  **p-value** |
| --- | --- | --- | --- | --- | --- |
| control | UI | 34 | 7 | 4 | control VS tKO UI  p=0.8544  ns |
| tKO |  | 36 | 12 | 4 |  |
| control | 10dpi | 950 | 116 | 4 | control VS tKO 10dpi  p<0.0001  *** |
| tKO |  | 3447 | 236 | 4 |  |
| control | 20dpi | 184 | 15 | 5 | control VS tKO 20dpi  p=0.0002  *** |
| tKO |  | 2885 | 209 | 5 |  |
| control | 30dpi | 76 | 16 | 5 | control VS tKO 30dpi  p=0.0044  ** |
| tKO |  | 1925 | 319 | 5 |  |
| control | 60dpi | 69 | 11 | 5 | control VS tKO 60dpi  p=0.0102  * |
| tKO |  | 257 | 43 | 5 |  |

Unpaired t test & Unpaired t test with Welch´s correlation

Figure 5I – TEM Unmyelinated >1.5µm axons in 1:1 relationship

| **Genotype** | **Time** | **Mean** | **SEM** | **n** | **Comparisons made**  **p-value** |
| --- | --- | --- | --- | --- | --- |
| control | UI | 2 | 1 | 4 | control VS tKO UI  p=0.1049  ns |
| tKO |  | 16 | 6 | 4 |  |
| control | 10dpi | 864 | 102 | 4 | control VS tKO 10dpi  p=0.0006  *** |
| tKO |  | 2405 | 209 | 4 |  |
| control | 20dpi | 110 | 13 | 5 | control VS tKO 20dpi  p=0.0001  *** |
| tKO |  | 2487 | 170 | 5 |  |
| control | 30dpi | 43 | 8 | 5 | control VS tKO 30dpi  p=0.0025  ** |
| tKO |  | 1728 | 250 | 5 |  |
| control | 60dpi | 28 | 5 | 5 | control VS tKO 60dpi  p=0.0095  ** |
| tKO |  | 224 | 43 | 5 |  |

Unpaired t test & Unpaired t test with Welch´s correlation

Figure 5J – TEM Total axon number >1.5µm

| **Genotype** | **Time** | **Mean** | **SEM** | **n** | **Comparisons made**  **p-value** |
| --- | --- | --- | --- | --- | --- |
| control | UI | 6415 | 94 | 4 | control VS tKO UI  p=0.1343  ns |
| tKO |  | 6057 | 184 | 4 |  |
| control | 10dpi | 3333 | 63 | 4 | control VS tKO 10dpi  p=0.6553  ns |
| tKO |  | 3447 | 236 | 4 |  |
| control | 20dpi | 5709 | 228 | 5 | control VS tKO 20dpi  p<0.0001  *** |
| tKO |  | 3492 | 184 | 5 |  |
| control | 30dpi | 6080 | 131 | 5 | control VS tKO 30dpi  p=0.0002  *** |
| tKO |  | 4584 | 184 | 5 |  |
| control | 60dpi | 6758 | 221 | 5 | control VS tKO 60dpi  p=0.8895  ns |
| tKO |  | 6716 | 198 | 5 |  |

Unpaired t test

Figure 5K – TEM Total axon number >1.5µm axons in 1:1 relationship

| **Genotype** | **Time** | **Mean** | **SEM** | **n** | **Comparisons made**  **p-value** |
| --- | --- | --- | --- | --- | --- |
| control | UI | 6383 | 89 | 4 | control VS tKO UI  p=0.1356  ns |
| tKO |  | 6036 | 181 | 4 |  |
| control | 10dpi | 3247 | 60 | 4 | control VS tKO 10dpi  p=0.0082  ** |
| tKO |  | 2405 | 209 | 4 |  |
| control | 20dpi | 5635 | 233 | 5 | control VS tKO 20dpi  p<0.0001  *** |
| tKO |  | 3093 | 147 | 5 |  |
| control | 30dpi | 6046 | 127 | 5 | control VS tKO 30dpi  p<0.0001  *** |
| tKO |  | 4387 | 158 | 5 |  |
| control | 60dpi | 6717 | 213 | 5 | control VS tKO 60dpi  p=0.9077  *** |
| tKO |  | 6682 | 198 | 5 |  |

Unpaired t test

Figure 5L – TEM Schwann cell nuclei

| **Genotype** | **Time** | **Mean** | **SEM** | **n** | **Comparisons made**  **p-value** |
| --- | --- | --- | --- | --- | --- |
| control | UI | 254 | 4 | 4 | control VS tKO UI  p=0.0705  ns |
| tKO |  | 329 | 28 | 4 |  |
| control | 10dpi | 609 | 33 | 4 | control VS tKO 10dpi  p=0.0068  ** |
| tKO |  | 1017 | 95 | 4 |  |
| control | 20dpi | 779 | 54 | 5 | control VS tKO 20dpi  p=0.0001  *** |
| tKO |  | 1576 | 100 | 5 |  |
| control | 30dpi | 723 | 47 | 5 | control VS tKO 30dpi  p<0.0001  *** |
| tKO |  | 1618 | 116 | 5 |  |
| control | 60dpi | 632 | 13 | 5 | control VS tKO 60dpi  p=0.0002  *** |
| tKO |  | 1237 | 53 | 5 |  |

Unpaired t test & Unpaired t test with Welch´s correlation

Figure 5M – TEM Myelinating Schwann cell nuclei

| **Genotype** | **Time** | **Mean** | **SEM** | **n** | **Comparisons made**  **p-value** |
| --- | --- | --- | --- | --- | --- |
| control | UI | 191 | 7 | 4 | control VS tKO UI  p=0.5889  ns |
| tKO |  | 201 | 17 | 4 |  |
| control | 10dpi | 212 | 18 | 4 | control VS tKO 10dpi  p<0.0001  *** |
| tKO |  | 0 | 0 | 4 |  |
| control | 20dpi | 326 | 33 | 5 | control VS tKO 20dpi  p<0.0001  *** |
| tKO |  | 48 | 12 | 5 |  |
| control | 30dpi | 325 | 17 | 5 | control VS tKO 30dpi  p=0.0011  ** |
| tKO |  | 181 | 24 | 5 |  |
| control | 60dpi | 348 | 18 | 5 | control VS tKO 60dpi  p=0.7832  ns |
| tKO |  | 358 | 31 | 5 |  |

Unpaired t test

Figure 5N – % Axons >1.5µm

| **Genotype** | **Time** | **Mean** | **SEM** | **n** | **Comparisons made**  **p-value** |
| --- | --- | --- | --- | --- | --- |
| control | UI | 100 | 0.0 | 4 | control VS tKO UI  p=0.0905  ns |
| tKO |  | 99.8 | 0.01 | 4 |  |
| control | 10dpi | 73.4 | 3.1 | 4 | control VS tKO 10dpi  p<0.0001  *** |
| tKO |  | 0 | 0.0 | 4 |  |
| control | 20dpi | 98.1 | 0.2 | 5 | control VS tKO 20dpi  p=0.0001  *** |
| tKO |  | 19.2 | 5.5 | 5 |  |
| control | 30dpi | 99.3 | 0.1 | 5 | control VS tKO 30dpi  p=0.0031  ** |
| tKO |  | 60.3 | 6.1 | 5 |  |
| control | 60dpi | 99.6 | 0.1 | 5 | control VS tKO 60dpi  p=0.0135  ** |
| tKO |  | 96.6 | 0.7 | 5 |  |

Unpaired t test & Unpaired t test with Welch´s correlati

Figure 5O – Western Blot 10 y 21 dpi WT. control and tKO

| **Genotype** | **JUN** | **Mean** | **SEM** | **n** | **Comparisons made**  **p-value** |
| --- | --- | --- | --- | --- | --- |
| WT | UI | 0.48 | 0.03 | 8 | N / A |
| WT | 10dpi | 1 | 0 | 4 | WT VS control  p=0.9665  ns |
| control |  | 1.07 | 0.07 | 4 | WT VS tKO  p<0.0001  *** |
| tKO |  | 3.24 | 0.35 | 4 | control VS tKO  p<0.0001  *** |

One-way ANOVA tukey’s test P=<0.0001 ***

| **Genotype** | **JUN** | **Mean** | **SEM** | **n** | **Comparisons made**  **p-value** |
| --- | --- | --- | --- | --- | --- |
| WT | 21dpi | 0.9 | 0.17 | 4 | WT VS control  p=0.8175  ns |
| control |  | 1.12 | 0.16 | 4 | WT VS tKO  p=0.0120  * |
| tKO |  | 2.25 | 0.38 | 4 | control VS tKO  p=0.0307  * |

One-way ANOVA tukey’s test P=0.0103

| **Genotype** | **KROX-20** | **Mean** | **SEM** | **n** | **Comparisons made**  **p-value** |
| --- | --- | --- | --- | --- | --- |
| WT | UI | 0.89 | 0.09 | 8 | N / A |
| WT | 10dpi | 1 | 0 | 4 | WT VS control  p=0.5526  ns |
| control |  | 0.91 | 0.05 | 4 | WT VS tKO  p=0.0023  ** |
| tKO |  | 0.61 | 0.08 | 4 | control VS tKO  p=0.0108  * |

One-way ANOVA tukey’s test P=0.0022 **

| **Genotype** | **KROX-20** | **Mean** | **SEM** | **n** | **Comparisons made**  **p-value** |
| --- | --- | --- | --- | --- | --- |
| WT | 21dpi | 0.83 | 0.05 | 4 | WT VS control  p=0.3167  ns |
| control |  | 0.94 | 0.02 | 4 | WT VS tKO  p=0.9887  ns |
| tKO |  | 0.84 | 0.06 | 4 | control VS tKO  p=0.3804  ns |

One-way ANOVA tukey’s test P=0.2446 ns

| **Genotype** | **MPZ** | **Mean** | **SEM** | **n** | **Comparisons made**  **p-value** |
| --- | --- | --- | --- | --- | --- |
| WT | UI | 1 | 0.07 | 8 | N / A |
| WT | 10dpi | 0.14 | 0.01 | 4 | WT VS control  p=0.0601  ns |
| control |  | 0.11 | 0.01 | 4 | WT VS tKO  p=0.0015  ** |
| tKO |  | 0.07 | 0.01 | 4 | control VS tKO  p=0.0771  ns |

One-way ANOVA tukey’s test P=0.0020 **

| **Genotype** | **MPZ** | **Mean** | **SEM** | **N** | **Comparisons made**  **p-value** |
| --- | --- | --- | --- | --- | --- |
| WT | 21dpi | 0.32 | 0.04 | 4 | WT VS control  p=0.6837  ns |
| control |  | 0.36 | 0.03 | 4 | WT VS tKO  p=0.0332  * |
| tKO |  | 0.15 | 0.04 | 4 | control VS tKO  p=0.0091  ** |

One-way ANOVA tukey’s test P=0.0086 **

**Figure 6**

Figure 6B – Voltage (V) A fibers

| **Genotype** | **Stimulus Intensity (V)** | **Mean** | **SEM** | **n** | **Comparisons made**  **p-value** |
| --- | --- | --- | --- | --- | --- |
| control | 5 | 0.31 | 0.19 | 16 | control VS tKO  p>0.1  ns |
| tKO |  | 0.21 | 0.14 | 7 |  |
| control | 8 | 1.77 | 0.64 | 18 | control VS tKO  p>0.1  ns |
| tKO |  | 1.83 | 0.56 | 8 |  |
| control | 10 | 1.91 | 0.70 | 18 | control VS tKO  p>0.1  ns |
| tKO |  | 1.69 | 0.48 | 8 |  |
| control | 15 | 2.45 | 0.79 | 18 | control VS tKO  p>0.1  ns |
| tKO |  | 1.86 | 0.88 | 8 |  |

Statistic Test-> Mann-Whitney’s U for non-parametric paired comparisons

Figure 6C – Voltage (V) C fibers

| **Genotype** | **Stimulus Intensity (V)** | **Mean** | **SEM** | **n** | **Comparisons made**  **p-value** |
| --- | --- | --- | --- | --- | --- |
| control | 5 | 0.008 | 0.005 | 17 | control VS tKO  p>0.1  ns |
| tKO |  | 0.014 | 0.013 | 7 |  |
| control | 8 | 0.064 | 0.019 | 18 | control VS tKO  p>0.1  ns |
| tKO |  | 0.101 | 0.04 | 8 |  |
| control | 10 | 0.082 | 0.020 | 18 | control VS tKO  p>0.1  ns |
| tKO |  | 0.113 | 0.037 | 8 |  |
| control | 15 | 0.156 | 0.068 | 18 | control VS tKO  p>0.1  ns |
| tKO |  | 0.116 | 0.039 | 8 |  |

Statistic Test-> Mann-Whitney’s U for non-parametric paired comparisons

Figure 6D – Nerve Conduction Velocity (m/s)

| **Genotype** | **Stimulus Intensity (V)** | **Mean** | **SEM** | **n** | **Comparisons made**  **p-value** |
| --- | --- | --- | --- | --- | --- |
| control | 5 | 17.7 | 7.4 | 16 | control VS tKO  p>0.1  ns |
| tKO |  | 7.1 | 5.7 | 7 |  |
| control | 8 | 21.7 | 6.7 | 18 | control VS tKO  p>0.1  ns |
| tKO |  | 16.9 | 6.4 | 8 |  |
| control | 10 | 19.1 | 6.9 | 18 | control VS tKO  p>0.1  ns |
| tKO |  | 15.3 | 5.7 | 8 |  |
| control | 15 | 18.1 | 6.7 | 18 | control VS tKO  p>0.1  ns |
| tKO |  | 11.6 | 6.4 | 8 |  |

Statistic Test-> Mann-Whitney’s U for non-parametric paired comparisons

Figure 6F – Voltage (V) A fibers

| **Genotype** | **Stimulus Intensity (V)** | **Mean** | **SEM** | **n** | **Comparisons made**  **p-value** |
| --- | --- | --- | --- | --- | --- |
| control | 5 | 0 | 0 | 7 | N/A |
| tKO |  | 0 | 0 | 7 |  |
| control | 8 | 0.262 | 0.116 | 9 | control VS tKO  p=0.018  * |
| tKO |  | 0.003 | 0.003 | 8 |  |
| control | 10 | 0.326 | 0.165 | 9 | control VS tKO  p=0.043  * |
| tKO |  | 0.006 | 0.006 | 8 |  |
| control | 15 | 0.466 | 0.215 | 9 | control VS tKO  p=0.046  * |
| tKO |  | 0.068 | 0.030 | 8 |  |

Statistic Test-> Mann-Whitney’s U for non-parametric paired comparisons

Figure 6G – Voltage (V) C fibers

| **Genotype** | **Stimulus Intensity (V)** | **Mean** | **SEM** | **N** | **Comparisons made**  **p-value** |
| --- | --- | --- | --- | --- | --- |
| control | 5 | 0 | 0 | 7 | N/A |
| tKO |  | 0 | 0 | 7 |  |
| control | 8 | 0.023 | 0.009 | 9 | control VS tKO  p=0.036  * |
| tKO |  | 0.004 | 0.004 | 8 |  |
| control | 10 | 0.079 | 0.030 | 9 | control VS tKO  p=0.021  * |
| tKO |  | 0.006 | 0.006 | 8 |  |
| control | 15 | 0.127 | 0.046 | 9 | control VS tKO  p>0.1  ns |
| tKO |  | 0.040 | 0.016 | 8 |  |

Statistic Test-> Mann-Whitney’s U for non-parametric paired comparisons

Figure 6H – Nerve Conduction Velocity (m/s)

| **Genotype** | **Stimulus Intensity (V)** | **Mean** | **SEM** | **n** | **Comparisons made**  **p-value** |
| --- | --- | --- | --- | --- | --- |
| control | 5 | 0 | 0 | 7 | N/A |
| tKO |  | 0 | 0 | 7 |  |
| control | 8 | 2.31 | 1.03 | 9 | control VS tKO  p=0.056  ns |
| tKO |  | 0.10 | 0.10 | 8 |  |
| control | 10 | 3.84 | 2.73 | 9 | control VS tKO  p=0.056  ns |
| tKO |  | 0.17 | 0.17 | 8 |  |
| control | 15 | 9.17 | 5.88 | 9 | control VS tKO  p=0.049  * |
| tKO |  | 0.39 | 0.17 | 8 |  |

Statistic Test-> Mann-Whitney’s U for non-parametric paired comparisons

Figure 6I – Mouse number

| **Genotype** | **Stimulus Intensity (V)** | **Mean** | **SEM** | **N** | **Comparisons made**  **p-value** |
| --- | --- | --- | --- | --- | --- |
| control | 5 | 0 |  | 9 | N/A |
| tKO |  | 0 |  | 8 |  |
| control | 8 | 6 |  | 9 | control VS tKO  p=0.023  * |
| tKO |  | 1 |  | 8 |  |
| control | 10 | 6 |  | 9 | control VS tKO  p=0.023  * |
| tKO |  | 1 |  | 8 |  |
| control | 15 | 7 |  | 9 | control VS tKO  p=0.079  ns |
| tKO |  | 4 |  | 8 |  |

Statistic Test-> Chi-Squared test

**Figure 7**

Figure 7H – ChIP *Pou3f1* Promoter

| **Antibody** | **Promoter** | **Mean** | **SEM** | **n** | **Comparisons made**  **p-value** |
| --- | --- | --- | --- | --- | --- |
| IgG | *Pou3f1* | 0.02 | 0.01 | 4 | IgG VS HDAC4  p=0.0286  * |
| HDAC4 |  | 10.04 | 3.2 | 4 |  |

Mann-Whitney test

**Figure 8**

Figure 8A – ChIP JUN in the *Hdac7* Promoter

| **Antibody** | **Promoter** | **Mean** | **SEM** | **n** | **Comparisons made**  **p-value** |
| --- | --- | --- | --- | --- | --- |
| IgG | *Hdac7* | 0.05 | 0.02 | 4 | IgG VS J  p=0.0472  * |
| JUN |  | 0.95 | 0.29 | 4 |  |

Paired t test

Figure 8B – Luciferase Jun in the *Hdac7* Promoter

| **Plasmid** | **Promoter** | **Mean** | **SEM** | **n** | **Comparisons made**  **p-value** |
| --- | --- | --- | --- | --- | --- |
| *pCDNA3* | *Hdac7* | 1 | 0.10 | 10 | *pCDNA3* VS *Jun*  p<0.0001  *** |
| *Jun* |  | 3.44 | 0.22 | 10 |  |

Unpaired t test with Welch´s correlation

Figure 8C – *Jun* mRNA level relative to 18S

| **Genotype** | **Gen** | **Mean** | **SEM** | **n** | **Comparisons made**  **p-value** |
| --- | --- | --- | --- | --- | --- |
| WT | *Jun* | 0.79x10^-4^ | 0.06x10^-4^ | 5 | WT VS Jun_OE  p=0.0022  ** |
| Jun_OE |  | 7.06x10^-4^ | 1x10^-4^ | 5 |  |

Unpaired t test with Welch´s correlation

| **Genotype** | **Gen** | **Mean** | **SEM** | **n** | **Comparisons made**  **p-value** |
| --- | --- | --- | --- | --- | --- |
| WT | *Jun* | 0.61x10^-4^ | 0.02x10^-4^ | 4 | WT VS Jun_cKO  p=0.0002  *** |
| Jun_cKO |  | 0.39x10^-4^ | 0.02x10^-4^ | 4 |  |

Unpaired t test

Figure 8D – *Hdac7* mRNA level relative to 18S

| **Genotype** | **Gen** | **Mean** | **SEM** | **n** | **Comparisons made**  **p-value** |
| --- | --- | --- | --- | --- | --- |
| WT | *Hdac7* | 3.09x10^-5^ | 0.29x10^-5^ | 5 | WT VS Jun_OE  p=0.0054  ** |
| Jun_OE |  | 5.16x10^-5^ | 0.46x10^-5^ | 5 |  |

Unpaired t test

| **Genotype** | **Gen** | **Mean** | **SEM** | **n** | **Comparisons made**  **p-value** |
| --- | --- | --- | --- | --- | --- |
| WT | *Hdac7* | 2.84x10^-5^ | 0.28x10^-5^ | 5 | WT VS Jun_cKO  p=0.9220  ns |
| Jun_cKO |  | 2.83x10^-5^ | 0.05x10^-5^ | 5 |  |

Unpaired t test with Welch´s correlation

Figure 8E – *Hdac4* mRNA level relative to 18S

| **Genotype** | **Gen** | **Mean** | **SEM** | **n** | **Comparisons made**  **p-value** |
| --- | --- | --- | --- | --- | --- |
| WT | *Hdac4* | 4.45x10^-5^ | 0.59x10^-5^ | 5 | WT VS Jun_OE  p=0.4103  ns |
| Jun_OE |  | 5.23x10^-5^ | 0.67x10^-5^ | 5 |  |

Unpaired t test

| **Genotype** | **Gen** | **Mean** | **SEM** | **n** | **Comparisons made**  **p-value** |
| --- | --- | --- | --- | --- | --- |
| WT | *Hdac4* | 3.67x10^-5^ | 0.22x10^-5^ | 4 | WT VS Jun_cKO  p=0.8641  ns |
| Jun_cKO |  | 3.72x10^-5^ | 0.17x10^-5^ | 4 |  |

Unpaired t test

Figure 8F – *Hdac5* mRNA level relative to 18S

| **Genotype** | **Gen** | **Mean** | **SEM** | **n** | **Comparisons made**  **p-value** |
| --- | --- | --- | --- | --- | --- |
| WT | *Hdac5* | 3.05x10^-5^ | 0.38x10^-5^ | 5 | WT VS Jun_OE  p=0.8888  ns |
| Jun_OE |  | 3.13x10^-5^ | 0.45x10^-5^ | 5 |  |

Unpaired t test

| **Genotype** | **Gen** | **Mean** | **SEM** | **n** | **Comparisons made**  **p-value** |
| --- | --- | --- | --- | --- | --- |
| WT | *Hdac5* | 2.60x10^-5^ | 0.03x10^-5^ | 4 | WT VS Jun_cKO  p=0.4485  ns |
| Jun_cKO |  | 2.71x10^-5^ | 0.13x10^-5^ | 4 |  |

Unpaired t test with Welch´s correlation

Figure 8G – HDAC4 ChiP in the *Hdac7* Promoter

| **Antibody** | **Promoter** | **Mean** | **SEM** | **n** | **Comparisons made**  **p-value** |
| --- | --- | --- | --- | --- | --- |
| IgG | *Hdac7* | 0.01 | 0.003 | 4 | IgG VS HDAC4  p=0.0300  * |
| HDAC4 |  | 10.06 | 2.58 | 4 |  |

Paired t test

Figure 8I – *Hdac7* qPCR dKO;Jun_cKO normalize to control *development*

| **Age** | **Gen** | **Mean** | **SEM** | **n** | **p-value** |
| --- | --- | --- | --- | --- | --- |
| P8 | *Hdac7* | 95.03 | 2.64 | 4 | p=0.9431  ns |
| P60 |  | 103.11 | 7.37 | 5 | p=0.7155  ns |

Unpaired t test

Figure 8J – *Hdac9* qPCR dKO;Jun_cKO normalize to control development

| **Age** | **Gen** | **Mean** | **SEM** | **n** | **p-value** |
| --- | --- | --- | --- | --- | --- |
| P8 | *Hdac9* | 113.19 | 3.89 | 4 | p=0.8936  ns |
| P60 |  | 153.84 | 16.20 | 5 | p=0.0208  * |

Unpaired t test

**Figure 9**

Figure 9A – *Hdac9* mRNA level relative to 18S

| **Genotype** | **Line** | **Mean** | **SEM** | **n** | **Comparisons made**  **p-value** |
| --- | --- | --- | --- | --- | --- |
| control | cKO4 | 0.88x10^-6^ | 0.03x10^-6^ | 4 | control VS cKO4  p=0.0121  * |
| cKO4 |  | 1.18x10^-6^ | 0.08x10^-6^ | 4 |  |
| WT | KO5 | 0.17x10^-6^ | 0.04x10^-6^ | 5 | WT VS KO5  p=0.3066  ns |
| KO5 |  | 0.22x10^-6^ | 0.03x10^-6^ | 6 |  |
| control | cKO7 | 0.96x10^-6^ | 0.18x10^-6^ | 4 | control VS cKO7  P=0.0057  ** |
| cKO7 |  | 2.32x10^-6^ | 0.27x10^-6^ | 4 |  |
| control | dKO | 0.55x10^-6^ | 0.18x10^-6^ | 8 | control VS dKO  p=0.8093  ns |
| dKO |  | 0.61x10^-6^ | 0.15x10^-6^ | 8 |  |
| control | tKO | 1.46x10^-6^ | 0.28x10^-6^ | 8 | control VS tKO  P=<0.0001  *** |
| tKO |  | 6.48x10^-6^ | 0.53x10^-6^ | 7 |  |

Unpaired t test

Figure 9B – *Hdac9* mRNA level relative to 18S during development tKO

| **Genotype** | **Development** | **Mean** | **SEM** | **n** | **Comparisons made**  **p-value** |
| --- | --- | --- | --- | --- | --- |
| control | P2 | 0.39x10^-6^ | 0.03x10^-6^ | 5 | control VS tKO P2  p=0.0004  *** |
| tKO |  | 1.67x10^-6^ | 0.13x10^-6^ | 5 |  |
| control | P8 | 0.65x10^-6^ | 0.09x10^-6^ | 4 | control VS tKO P8  p=0.0117  * |
| tKO |  | 3.43x10^-6^ | 0.52x10^-6^ | 4 |  |

Unpaired t test with Welch´s correlation

Figure 9C – ChIP IgG and H3K9Ac relative enrichment UI control and tKO *Hdac9* promoter

| **Genotype** | **ChIP** | **Mean** | **SEM** | **n** | **Comparisons made**  **p-value** |
| --- | --- | --- | --- | --- | --- |
| control | H3K9AC | 16.75 | 7.39 | 3 | control VS tKO  p=0.0247  * |
| tKO |  | 57.11 | 8.81 | 3 |  |

Unpaired t test

Figure 9E – *Hdac9* RNA-seq tKO

| **Genotype** | **Dpi** | **Mean** | **SEM** | **n** | **Comparisons made**  **p-value** |
| --- | --- | --- | --- | --- | --- |
| control | UI | 0.66 | 0.01 | 3 | control VS tKO UI  p<0.0001  **** |
| tKO |  | 2.57 | 0.02 | 3 |  |
| control | 1 | 1.15 | 0.16 | 3 | control VS tKO 1dpi  p=0.0559  ns |
| tKO |  | 1.86 | 0.15 | 3 |  |
| control | 10 | 0.89 | 0.06 | 3 | control VS tKO 10dpi  p<0.0001  **** |
| tKO |  | 4.57 | 0.40 | 3 |  |
| control | 20 | 0.72 | 0.03 | 3 | control VS tKO 20dpi  p<0.0001  **** |
| tKO |  | 5.74 | 0.23 | 3 |  |

2 Way ANOVA with Sidak's multiple comparisons test

Figure 9F – *Mef2d* mRNA level relative to 18S during development tKO

| **Genotype** | **Development** | **Mean** | **SEM** | **n** | **Comparisons made**  **p-value** |
| --- | --- | --- | --- | --- | --- |
| control | P2 | 0.47x10^-5^ | 0.07x10^-5^ | 5 | control VS tKO P2  p=0.1216  ns |
| tKO |  | 0.65x10^-5^ | 0.07x10^-5^ | 5 |  |
| control | P8 | 0.97x10^-5^ | 0.10x10^-5^ | 4 | control VS tKO P8  p=0.0170  * |
| tKO |  | 1.65x10^-5^ | 0.18x10^-5^ | 4 |  |

Unpaired t test

Figure 9G – *Mef2d* RNA-seq tKO

| **Genotype** | **Dpi** | **Mean** | **SEM** | **n** | **Comparisons made**  **p-value** |
| --- | --- | --- | --- | --- | --- |
| control | UI | 7.71 | 0.34 | 3 | control VS tKO UI  p=0.8164  ns |
| tKO |  | 9.56 | 0.35 | 3 |  |
| control | 1 | 11.71 | 0.08 | 3 | control VS tKO 1dpi  p=0.9778  ns |
| tKO |  | 12.68 | 0.41 | 3 |  |
| control | 10 | 15.96 | 0.23 | 3 | control VS tKO 10dpi  p=0.0015  ** |
| tKO |  | 24.51 | 2.67 | 3 |  |
| control | 20 | 12.74 | 0.77 | 3 | control VS tKO 20dpi  p=0.0025  ** |
| tKO |  | 20.82 | 2.50 | 3 |  |

2 Way ANOVA with Sidak's multiple comparisons test

Figure 9H – MEF2D WB UI-10dpi

| **Genotype** | **Dpi** | **Mean** | **SEM** | **n** | **Comparisons made**  **p-value** |
| --- | --- | --- | --- | --- | --- |
| control | UI | 1.27 | 0.13 | 3 | control VS tKO UI  p>0.9996  ns |
| tKO |  | 1.20 | 0.07 | 4 |  |
| control | 10 | 1.33 | 0.19 | 3 | control 10dpi VS tKO 10dpi  p=0.0069  **  tKO UI VS tKO 10 dpi  p=0.0011  ** |
| tKO |  | 2.31 | 0.19 | 4 |  |

ANOVA with Tukey's multiple comparisons test

Figure 9I – MEF2D WB UI-20dpi

| **Genotype** | **Dpi** | **Mean** | **SEM** | **n** | **Comparisons made**  **p-value** |
| --- | --- | --- | --- | --- | --- |
| control | UI | 0.84 | 0.20 | 3 | control VS tKO UI  p>0.9999  ns |
| tKO |  | 0.83 | 0.26 | 3 |  |
| control | 20 | 1.33 | 0.11 | 3 | Control 20dpi VS tKO 20dpi  p=0.0073  **  tKO UI VS tKO 20 dpi  p=0.004  *** |
| tKO |  | 2.19 | 0.03 | 3 |  |

ANOVA with Tukey's multiple comparisons test

Figure 9K – ChIP IgG and MEF2D relative enrichment 15-20dpi tKO *Hdac9 promoter*

| **ChIP** | **Genotype** | **Mean** | **SEM** | **n** | **Comparisons made**  **p-value** |
| --- | --- | --- | --- | --- | --- |
| IgG | tKO | 1 | 0 | 5 | control VS tKO  p<0.0001  *** |
| MEF2D |  | 3.26 | 0.27 | 5 |  |

Unpaired t test

**Figure 1 – figure supplement 1**

Figure 1 – figure supplement 1A – Compensation cKO4 mRNA % of control littermate

| **Gen** | **Age** | **Mean** | **SEM** | **n** | **Comparisons made**  **p-value** |
| --- | --- | --- | --- | --- | --- |
| *Hdac5* | P60 | 109.18 | 3.54 | 4 | control VS cKO4  p=0.0412  * |
| *Hdac7* |  | 97.49 | 3.97 | 4 | control VS cKO4  p=0.5510  ns |
| *Hdac9* |  | 134.25 | 6.93 | 4 | control VS cKO4  p=0.0026  ** |

Unpaired t test

Figure 1 – figure supplement 1B – Compensation KO5 mRNA % of WT littermate

| **Gen** | **Age** | **Mean** | **SEM** | **n** | **Comparisons made**  **p-value** |
| --- | --- | --- | --- | --- | --- |
| *Hdac4* | P60 | 121.24 | 10.26 | 4 | WT VS KO5  p=0.0838  ns |
| *Hdac7* |  | 103.18 | 5.93 | 7 | WT VS KO5  p=0.6021  ns |
| *Hdac9* |  | 134.68 | 20.29 | 5 | WT VS KO5  p=0.1258  ns |

Unpaired t test

Figure 1 – figure supplement 1C – qPCR dKO normalize to control *development*

| **Age** | **Gen** | **Mean** | **SEM** | **n** | **Comparisons made**  **p-value** |
| --- | --- | --- | --- | --- | --- |
| P2 | *Hdac7* | 133.3 | 6.31 | 4 | p<0.0019 ** |
| P8 |  | 131.4 | 9.03 | 4 | p<0.0139 * |

Unpaired t test

Figure 1 – figure supplement 1D – qPCR cKO4 normalize to control *Hdac4*

| **Genotype** | **Gen** | **Mean** | **SEM** | **n** | **Comparisons made**  **p-value** |
| --- | --- | --- | --- | --- | --- |
| control | *Hdac4* | 100 | 0 | 5 | control VS cKO4  p<0.0001  *** |
| cKO4 |  | 40.08 | 1.60 | 5 |  |

Unpaired t test

Figure 1 – figure supplement 1E – qPCR KO5 normalize to WT *Hdac5*

| **Genotype** | **Gen** | **Mean** | **SEM** | **n** | **Comparisons made**  **p-value** |
| --- | --- | --- | --- | --- | --- |
| WT | *Hdac5* | 100 | 0 | 4 | WT VS KO5  p<0.0001  *** |
| KO5 |  | 0.38 | 0.04 | 4 |  |

Unpaired t test

Figure 1 – figure supplement 1F – qPCR cKO7 normalize to control *Hdac7*

| **Genotype** | **Gen** | **Mean** | **SEM** | **n** | **Comparisons made**  **p-value** |
| --- | --- | --- | --- | --- | --- |
| control | *Hdac7* | 100 | 0 | 7 | control VS cKO7  p=0.0021  ** |
| cKO7 |  | 57.97 | 10.77 | 7 |  |

Unpaired t test

**Figure 1 – figure supplement 2**

Figure 1 – figure supplement 2B – TEM Nerve area (µ^2^)

| **Genotype** | **Age** | **Mean** | **SEM** | **n** | **Comparisons made**  **p-value** |
| --- | --- | --- | --- | --- | --- |
| control | P2 | 30780 | 1485 | 4 | control VS cKO4 P2  p=0.6148  ns |
| cKO4 |  | 31570 | 655 | 5 |  |
| control | P8 | 75991 | 3977 | 4 | control VS cKO4 P8  p=0.3640  ns |
| cKO4 |  | 83507 | 6538 | 4 |  |

Unpaired t test

Figure 1 – figure supplement 2C – TEM Myelinated axons

| **Genotype** | **Age** | **Mean** | **SEM** | **n** | **Comparisons made**  **p-value** |
| --- | --- | --- | --- | --- | --- |
| control | P2 | 1363 | 29 | 4 | control VS cKO4 P2  p<0.0001  *** |
| cKO4 |  | 912 | 25 | 5 |  |
| control | P8 | 4718 | 185 | 4 | control VS cKO4 P8  p=0.5227  ns |
| cKO4 |  | 4921 | 236 | 4 |  |

Unpaired t test

Figure 1 – figure supplement 2D – TEM g-ratio

| **Genotype** | **Age** | **Mean** | **SEM** | **n** | **Comparisons made**  **p-value** |
| --- | --- | --- | --- | --- | --- |
| control | P8 | 0.71 | 0.01 | 3 | control VS cKO4 P8  p=0.5478  ns |
| cKO4 |  | 0.72 | 0.01 | 4 |  |

Unpaired t test

Figure 1 – figure supplement 2E – TEM Unmyelinated axons in 1:1 relationship

| **Genotype** | **Age** | **Mean** | **SEM** | **n** | **Comparisons made**  **p-value** |
| --- | --- | --- | --- | --- | --- |
| control | P2 | 1078 | 129 | 4 | control VS cKO4 P2  p=0.1455  ns |
| cKO4 |  | 1312 | 78 | 5 |  |
| control | P8 | 625 | 45 | 4 | control VS cKO4 P8  p=0.0234  * |
| cKO4 |  | 845 | 57 | 4 |  |

Unpaired t test

Figure 1 – figure supplement 2F – TEM Total axons number in 1:1 relationship

| **Genotype** | **Age** | **Mean** | **SEM** | **n** | **Comparisons made**  **p-value** |
| --- | --- | --- | --- | --- | --- |
| control | P2 | 2441 | 111 | 4 | control VS cKO4 P2  p=0.1370  ns |
| cKO4 |  | 2223 | 75 | 5 |  |
| control | P8 | 5342 | 226 | 4 | control VS cKO4 P8  p=0.2926  ns |
| cKO4 |  | 5766 | 289 | 4 |  |

Unpaired t test

Figure 1 – figure supplement 2G – TEM Schwann cell nuclei

| **Genotype** | **Age** | **Mean** | **SEM** | **n** | **Comparisons made**  **p-value** |
| --- | --- | --- | --- | --- | --- |
| control | P2 | 333 | 25 | 4 | control VS cKO4 P2  p=0.0134  * |
| cKO4 |  | 421 | 13 | 5 |  |
| control | P8 | 501 | 42 | 4 | control VS cKO4 P8  p=0.0524  ns |
| cKO4 |  | 675 | 59 | 4 |  |

Unpaired t test

Figure 1 – figure supplement 2H – TEM Myelinating Schwann cell nuclei

| **Genotype** | **Age** | **Mean** | **SEM** | **n** | **Comparisons made**  **p-value** |
| --- | --- | --- | --- | --- | --- |
| control | P2 | 167 | 12 | 4 | control VS cKO4 P2  p=0.4633  ns |
| cKO4 |  | 156 | 8 | 5 |  |
| control | P8 | 333 | 24 | 4 | control VS cKO4 P8  p=0.0637  ns |
| cKO4 |  | 412 | 25 | 4 |  |

Unpaired t test

Figure 1 – figure supplement 2I – TEM % Axons

| **Genotype** | **Age** | **Mean** | **SEM** | **n** | **Comparisons made**  **p-value** |
| --- | --- | --- | --- | --- | --- |
| control | P2 | 56.29 | 3.39 | 4 | control VS cKO4 P2  p=0.0037  ** |
| cKO4 |  | 41.17 | 1.68 | 5 |  |
| control | P8 | 88.35 | 0.41 | 4 | control VS cKO4 P8  p=0.0020  ** |
| cKO4 |  | 85.37 | 0.40 | 4 |  |

Unpaired t test

**Figure 1 – figure supplement 3**

Figure 1 – figure supplement 3B – TEM Nerve area (µ^2^)

| **Genotype** | **Age** | **Mean** | **SEM** | **n** | **Comparisons made**  **p-value** |
| --- | --- | --- | --- | --- | --- |
| WT | P2 | 31440 | 3405 | 3 | WT VS KO5 P2  p=0.5767  ns |
| KO5 |  | 33609 | 1084 | 3 |  |
| WT | P8 | 72831 | 817 | 3 | WT VS KO5 P8  p=0.4838  ns |
| KO5 |  | 75216 | 2036 | 3 |  |

Unpaired t test

Figure 1 – figure supplement 3C – TEM Myelinated axons

| **Genotype** | **Age** | **Mean** | **SEM** | **n** | **Comparisons made**  **p-value** |
| --- | --- | --- | --- | --- | --- |
| WT | P2 | 1556 | 188 | 3 | WT VS KO5 P2  p=0.9828  ns |
| KO5 |  | 1561 | 110 | 3 |  |
| WT | P8 | 4871 | 417 | 3 | WT VS KO5 P8  p=0.5426  ns |
| KO5 |  | 4660 | 126 | 3 |  |

Unpaired t test

Figure 1 – figure supplement 3D – TEM g-ratio

| **Genotype** | **Age** | **Mean** | **SEM** | **n** | **Comparisons made**  **p-value** |
| --- | --- | --- | --- | --- | --- |
| WT | P8 | 0.72 | 0.01 | 3 | WT VS KO5 P8  p=0.9619  ns |
| KO5 |  | 0.72 | 0.01 | 3 |  |

Unpaired t test

Figure 1 – figure supplement 3E – TEM Unmyelinated axons in 1:1 relationship

| **Genotype** | **Age** | **Mean** | **SEM** | **n** | **Comparisons made**  **p-value** |
| --- | --- | --- | --- | --- | --- |
| WT | P2 | 932 | 149 | 3 | WT VS KO5 P2  p=0.3083  ns |
| KO5 |  | 1126 | 75 | 3 |  |
| WT | P8 | 503 | 57 | 3 | WT VS KO5 P8  p=0.0442  * |
| KO5 |  | 632 | 19 | 3 |  |

Unpaired t test

Figure 1 – figure supplement 3F – TEM Total axons number in 1:1 relationship

| **Genotype** | **Age** | **Mean** | **SEM** | **n** | **Comparisons made**  **p-value** |
| --- | --- | --- | --- | --- | --- |
| WT | P2 | 2488 | 331 | 3 | WT VS KO5 P2  p=0.6221  ns |
| KO5 |  | 2687 | 176 | 3 |  |
| WT | P8 | 5374 | 361 | 3 | WT VS KO5 P8  p=0.7944  ns |
| KO5 |  | 5292 | 128 | 3 |  |

Unpaired t test

Figure 1 – figure supplement 3G – TEM Schwann cell nuclei

| **Genotype** | **Age** | **Mean** | **SEM** | **n** | **Comparisons made**  **p-value** |
| --- | --- | --- | --- | --- | --- |
| WT | P2 | 328 | 53 | 3 | WT VS KO5 P2  p=0.4890  ns |
| KO5 |  | 370 | 18 | 3 |  |
| WT | P8 | 516 | 69 | 3 | WT VS KO5 P8  p=0.6832  ns |
| KO5 |  | 493 | 19 | 3 |  |

Unpaired t test

Figure 1 – figure supplement 3H – TEM Myelinating Schwann cell nuclei

| **Genotype** | **Age** | **Mean** | **SEM** | **n** | **Comparisons made**  **p-value** |
| --- | --- | --- | --- | --- | --- |
| WT | P2 | 146 | 25 | 3 | WT VS KO5 P2  p=0.6652  ns |
| KO5 |  | 158 | 4 | 3 |  |
| WT | P8 | 351 | 58 | 3 | WT VS KO5 P8  p=0.4684  ns |
| KO5 |  | 319 | 12 | 3 |  |

Unpaired t test

Figure 1 – figure supplement 3I – TEM % Axons

| **Genotype** | **Age** | **Mean** | **SEM** | **n** | **Comparisons made**  **p-value** |
| --- | --- | --- | --- | --- | --- |
| WT | P2 | 62.85 | 1.69 | 3 | WT VS KO5 P2  p=0.0727  ns |
| KO5 |  | 58.07 | 1.02 | 3 |  |
| WT | P8 | 90.54 | 1.70 | 3 | WT VS KO5 P8  p=0.1099  ns |
| KO5 |  | 88.04 | 0.43 | 3 |  |

Unpaired t test

**Figure 1 – figure supplement 4**

Figure 1 – figure supplement 4B – TEM Nerve area (µ^2^)

| **Genotype** | **Age** | **Mean** | **SEM** | **n** | **Comparisons made**  **p-value** |
| --- | --- | --- | --- | --- | --- |
| control | P2 | 32163 | 472 | 4 | control VS cKO7 P2  p=0.8714  ns |
| cKO7 |  | 32477 | 2099 | 3 |  |
| control | P8 | 73089 | 1276 | 5 | control VS cKO7 P8  p=0.2930  ns |
| cKO7 |  | 77890 | 4518 | 4 |  |

Unpaired t test

Figure 1 – figure supplement 4C – TEM Myelinated axons

| **Genotype** | **Age** | **Mean** | **SEM** | **n** | **Comparisons made**  **p-value** |
| --- | --- | --- | --- | --- | --- |
| control | P2 | 1076 | 51 | 4 | control VS cKO7 P2  p=0.6789  ns |
| cKO7 |  | 1111 | 61 | 3 |  |
| control | P8 | 4663 | 96 | 5 | control VS cKO7 P8  p=0.2050  ns |
| cKO7 |  | 4474 | 92 | 4 |  |

Unpaired t test

Figure 1 – figure supplement 4D – TEM g-ratio

| **Genotype** | **Age** | **Mean** | **SEM** | **n** | **Comparisons made**  **p-value** |
| --- | --- | --- | --- | --- | --- |
| control | P8 | 0.726 | 0.004 | 5 | control VS cKO7 P8  p=0.6326  ns |
| cKO7 |  | 0.729 | 0.004 | 4 |  |

Unpaired t test

Figure 1 – figure supplement 4E – TEM Unmyelinated axons in 1:1 relationship

| **Genotype** | **Age** | **Mean** | **SEM** | **n** | **Comparisons made**  **p-value** |
| --- | --- | --- | --- | --- | --- |
| control | P2 | 1460 | 102 | 4 | control VS cKO7 P2  p=0.4295  ns |
| cKO7 |  | 1340 | 83 | 3 |  |
| control | P8 | 691 | 52 | 5 | control VS cKO7 P8  p=0.8513  ns |
| cKO7 |  | 705 | 44 | 4 |  |

Unpaired t test

Figure 1 – figure supplement 4F – TEM Total axons number in 1:1 relationship

| **Genotype** | **Age** | **Mean** | **SEM** | **n** | **Comparisons made**  **p-value** |
| --- | --- | --- | --- | --- | --- |
| control | P2 | 2536 | 97 | 4 | control VS cKO7 P2  p=0.6201  ns |
| cKO7 |  | 2451 | 133 | 3 |  |
| control | P8 | 5354 | 101 | 5 | control VS cKO7 P8  p=0.2158  ns |
| cKO7 |  | 5179 | 67 | 4 |  |

Unpaired t test

Figure 1 – figure supplement 4G – TEM Schwann cell nuclei

| **Genotype** | **Age** | **Mean** | **SEM** | **n** | **Comparisons made**  **p-value** |
| --- | --- | --- | --- | --- | --- |
| control | P2 | 384 | 20 | 4 | control VS cKO7 P2  p=0.8209  ns |
| cKO7 |  | 390 | 12 | 3 |  |
| control | P8 | 562 | 19 | 5 | control VS cKO7 P8  p=0.6559  ns |
| cKO7 |  | 551 | 14 | 4 |  |

Unpaired t test

Figure 1 – figure supplement 4H – TEM Myelinating Schwann cell nuclei

| **Genotype** | **Age** | **Mean** | **SEM** | **n** | **Comparisons made**  **p-value** |
| --- | --- | --- | --- | --- | --- |
| control | P2 | 123 | 7 | 4 | control VS cKO7 P2  p=0.1050  ns |
| cKO7 |  | 141 | 5 | 3 |  |
| control | P8 | 349 | 9 | 5 | control VS cKO7 P8  p=0.1829  ns |
| cKO7 |  | 329 | 10 | 4 |  |

Unpaired t test

Figure 1 – figure supplement 4I – TEM % Axons

| **Genotype** | **Age** | **Mean** | **SEM** | **n** | **Comparisons made**  **p-value** |
| --- | --- | --- | --- | --- | --- |
| control | P2 | 42.59 | 2.41 | 4 | control VS cKO7 P2  p=0.4010  ns |
| cKO7 |  | 45.33 | 1.06 | 3 |  |
| control | P8 | 87.10 | 0.93 | 5 | control VS cKO7 P8  p=0.6049  ns |
| cKO7 |  | 86.38 | 0.94 | 4 |  |

Unpaired t test

**Figure 1 – figure supplement 5**

Figure 1 – figure supplement 5B – TEM Nerve area (µ^2^)

| **Genotype** | **Age** | **Mean** | **SEM** | **n** | **Comparisons made**  **p-value** |
| --- | --- | --- | --- | --- | --- |
| control | P2 | 31718 | 2229 | 4 | control VS dKO P2  p=0.6141  ns |
| dKO |  | 30097 | 2090 | 5 |  |
| control | P8 | 70586 | 1686 | 4 | control VS dKO P8  p=0.0585  ns |
| dKO |  | 78926 | 2984 | 5 |  |

Unpaired t test

Figure 1 – figure supplement 5C – TEM Myelinated axons

| **Genotype** | **Age** | **Mean** | **SEM** | **n** | **Comparisons made**  **p-value** |
| --- | --- | --- | --- | --- | --- |
| control | P2 | 1645 | 269 | 4 | control VS dKO P2  p=0.0124  * |
| dKO |  | 743 | 115 | 5 |  |
| control | P8 | 4687 | 130 | 4 | control VS dKO P8  p=0.4499  ns |
| dKO |  | 4540 | 127 | 5 |  |

Unpaired t test

Figure 1 – figure supplement 5D – TEM g-ratio

| **Genotype** | **Age** | **Mean** | **SEM** | **n** | **Comparisons made**  **p-value** |
| --- | --- | --- | --- | --- | --- |
| control | P8 | 0.72 | 0.014 | 4 | control VS dKO P8  p=0.7050  ns |
| dKO |  | 0.73 | 0.003 | 5 |  |

Unpaired t test

Figure 1 – figure supplement 5E – TEM Unmyelinated axons in 1:1 relationship

| **Genotype** | **Age** | **Mean** | **SEM** | **n** | **Comparisons made**  **p-value** |
| --- | --- | --- | --- | --- | --- |
| control | P2 | 1132 | 179 | 4 | control VS dKO P2  p=0.4028  ns |
| dKO |  | 1394 | 220 | 5 |  |
| control | P8 | 550 | 15 | 4 | control VS dKO P8  p=0.0029  ** |
| dKO |  | 721 | 32 | 5 |  |

Unpaired t test

Figure 1 – figure supplement 5F – TEM Total axons number in 1:1 relationship

| **Genotype** | **Age** | **Mean** | **SEM** | **n** | **Comparisons made**  **p-value** |
| --- | --- | --- | --- | --- | --- |
| control | P2 | 2776 | 435 | 4 | control VS dKO P2  p=0.2694  ns |
| dKO |  | 2136 | 328 | 5 |  |
| control | P8 | 5237 | 119 | 4 | control VS dKO P8  p=0.8975  ns |
| dKO |  | 5261 | 129 | 5 |  |

Unpaired t test

Figure 1 – figure supplement 5G – TEM Schwann cell nuclei

| **Genotype** | **Age** | **Mean** | **SEM** | **n** | **Comparisons made**  **p-value** |
| --- | --- | --- | --- | --- | --- |
| control | P2 | 358 | 38 | 4 | control VS dKO P2  p=0.4120  ns |
| dKO |  | 405 | 37 | 5 |  |
| control | P8 | 461 | 29 | 4 | control VS dKO P8  p=0.0388  * |
| dKO |  | 543 | 18 | 5 |  |

Unpaired t test

Figure 1 – figure supplement 5H – TEM Myelinating Schwann cell nuclei

| **Genotype** | **Age** | **Mean** | **SEM** | **n** | **Comparisons made**  **p-value** |
| --- | --- | --- | --- | --- | --- |
| control | P2 | 143 | 23 | 4 | control VS dKO P2  p=0.3542  ns |
| dKO |  | 118 | 12 | 5 |  |
| control | P8 | 309 | 21 | 4 | control VS dKO P8  p=0.2938  ns |
| dKO |  | 345 | 23 | 5 |  |

Unpaired t test

Figure 1 – figure supplement 5I – TEM % Axons

| **Genotype** | **Age** | **Mean** | **SEM** | **n** | **Comparisons made**  **p-value** |
| --- | --- | --- | --- | --- | --- |
| control | P2 | 59.29 | 1.97 | 4 | control VS dKO P2  p<0.0001  *** |
| dKO |  | 34.89 | 1.75 | 5 |  |
| control | P8 | 89.47 | 0.49 | 4 | control VS dKO P8  p=0.0061  ** |
| dKO |  | 86.27 | 0.62 | 5 |  |

Unpaired t test

**Figure 2 – figure supplement 1**

Figure 2 – figure supplement 1A – Remaks single Kos and dKO

| **Genotype** | **# axons per Remak Bundle** | **Mean** | **SEM** | **n** | **Comparisons made**  **p-value** |
| --- | --- | --- | --- | --- | --- |
| control | 2-5 | 26.29 | 4 | 3 | control VS cKO4  p=0.58  ns |
| cKO4 |  | 23.66 | 3 | 4 |  |
| control | 6-10 | 25.95 | 4 | 3 | control VS cKO4  p=0.494  ns |
| cKO4 |  | 22.85 | 2 | 4 |  |
| control | 11-15 | 18.09 | 3 | 3 | control VS cKO4  p=0.731  ns |
| cKO4 |  | 19.13 | 0.2 | 4 |  |
| control | 16-20 | 13.29 | 2.5 | 3 | control VS cKO4  p=0.226  ns |
| cKO4 |  | 9.01 | 2 | 4 |  |
| control | 21-30 | 10.39 | 2.6 | 3 | control VS cKO4  p=0.106  ns |
| cKO4 |  | 15.93 | 1.5 | 4 |  |
| control | +30 | 6 | 2 | 3 | control VS cKO4  p=0.332  ns |
| cKO4 |  | 9.42 | 2.2 | 4 |  |

Mixed Model ANOVA with Bonferroni post-hoc test (P=0.401)

| **Genotype** | **# axons per pocket** | **Mean** | **SEM** | **n** | **Comparisons made**  **p-value** |
| --- | --- | --- | --- | --- | --- |
| control | single | 88.08 | 3 | 3 | control VS cKO4  p=0.406  ns |
| cKO4 |  | 84.70 | 0.5 | 4 |  |
| control | 2-5 | 10.19 | 3 | 3 | control VS cKO4  p=0.748  ns |
| cKO4 |  | 11.22 | 0.5 | 4 |  |
| control | 6-10 | 1.37 | 0.4 | 3 | control VS cKO4  p=0.096  ns |
| cKO4 |  | 2.96 | 0.4 | 4 |  |
| control | 11-15 | 0.36 | 0.2 | 3 | control VS cKO4  p=0.125  ns |
| cKO4 |  | 0.92 | 0.2 | 4 |  |
| control | 16-20 | 0 | 0 | 3 | control VS cKO4  p=0.391  ns |
| cKO4 |  | 0.18 | 0.2 | 4 |  |
| control | 21-30 | 0 | 0 | 3 | control VS cKO4  p=NaN  ns |
| cKO4 |  | 0 | 0 | 4 |  |
| control | +30 | 0 | 0 | 3 | control VS cKO4  p=NaN  ns |
| cKO4 |  | 0 | 0 | 4 |  |

Mixed Model ANOVA with Bonferroni post-hoc test (P=0.384)

| **Genotype** | **# axons per Remak Bundle** | **Mean** | **SEM** | **n** | **Comparisons made**  **p-value** |
| --- | --- | --- | --- | --- | --- |
| WT | 2-5 | 23.08 | 3 | 4 | WT VS KO5  p=0.397  ns |
| KO5 |  | 27.15 | 2.4 | 3 |  |
| WT | 6-10 | 24.35 | 3 | 4 | WT VS KO5  p=0.483  ns |
| KO5 |  | 21.72 | 2 | 3 |  |
| WT | 11-15 | 19.64 | 3 | 4 | WT VS KO5  p=0.748  ns |
| KO5 |  | 18.20 | 3 | 3 |  |
| WT | 16-20 | 14.43 | 2.5 | 4 | WT VS KO5  p=0.869  ns |
| KO5 |  | 13.83 | 2 | 3 |  |
| WT | 21-30 | 10.58 | 1 | 4 | WT VS KO5  p=0.368  ns |
| KO5 |  | 12.12 | 1 | 3 |  |
| WT | +30 | 7.92 | 1 | 4 | WT VS KO5  p=0.56  ns |
| KO5 |  | 6.97 | 1.4 | 3 |  |

Mixed Model ANOVA with Bonferroni post-hoc test (P=0.081)

| **Genotype** | **# axons per pocket** | **Mean** | **SEM** | **n** | **Comparisons made**  **p-value** |
| --- | --- | --- | --- | --- | --- |
| WT | single | 91.38 | 1 | 4 | WT VS KO5  p=0.096  ns |
| KO5 |  | 87.74 | 2 | 4 |  |
| WT | 2-5 | 7.04 | 1 | 4 | WT VS KO5  p=0.084  ns |
| KO5 |  | 9.88 | 1 | 4 |  |
| WT | 6-10 | 1.33 | 0.5 | 4 | WT VS KO5  p=0.441  ns |
| KO5 |  | 1.97 | 1 | 4 |  |
| WT | 11-15 | 0.26 | 0.3 | 4 | WT VS KO5  p=0.626  ns |
| KO5 |  | 0.09 | 0.1 | 4 |  |
| WT | 16-20 | 0 | 0 | 4 | WT VS KO5  p=NaN  ns |
| KO5 |  | 0.00 | 0 | 4 |  |
| WT | 21-30 | 0 | 0 | 4 | WT VS KO5  p=0.072  ns |
| KO5 |  | 0.32 | 0.2 | 4 |  |
| WT | +30 | 0 | 0 | 4 | WT VS KO5  p=NaN  ns |
| KO5 |  | 0.00 | 0 | 4 |  |

Mixed Model ANOVA with Bonferroni post-hoc test (P=0.005)

| **Genotype** | **# axons per Remak Bundle** | **Mean** | **SEM** | **n** | **Comparisons made**  **p-value** |
| --- | --- | --- | --- | --- | --- |
| control | 2-5 | 23.66 | 2 | 3 | control VS cKO7  p=0.042  ns |
| cKO7 |  | 23.28 | 4 | 3 |  |
| control | 6-10 | 19.96 | 3 | 3 | control VS cKO7  p=0.445  ns |
| cKO7 |  | 23.81 | 3 | 3 |  |
| control | 11-15 | 17.26 | 2 | 3 | control VS cKO7  p=0.471  ns |
| cKO7 |  | 19.76 | 2 | 3 |  |
| control | 16-20 | 12.65 | 3 | 3 | control VS cKO7  p=0.887  ns |
| cKO7 |  | 13.14 | 1 | 3 |  |
| control | 21-30 | 16.34 | 4 | 3 | control VS cKO7  p=0.263  ns |
| cKO7 |  | 10.40 | 2 | 3 |  |
| control | +30 | 10.14 | 1 | 3 | control VS cKO7  p=0.904  ns |
| cKO7 |  | 9.61 | 4 | 3 |  |

Mixed Model ANOVA with Bonferroni post-hoc test (P=0.728)

| **Genotype** | **# axons per pocket** | **Mean** | **SEM** | **n** | **Comparisons made**  **p-value** |
| --- | --- | --- | --- | --- | --- |
| control | single | 92.01 | 0.3 | 3 | control VS cKO7  p=0.116  ns |
| cKO7 |  | 88.74 | 1 | 3 |  |
| control | 2-5 | 7.42 | 0.4 | 3 | control VS cKO7  p=0.501  ns |
| cKO7 |  | 8.47 | 1 | 3 |  |
| control | 6-10 | 0.57 | 0.1 | 3 | control VS cKO7  p=0.137  ns |
| cKO7 |  | 2.26 | 1 | 3 |  |
| control | 11-15 | 0 | 0 | 3 | control VS cKO7  p=0.286  ns |
| cKO7 |  | 0.13 | 0.1 | 3 |  |
| control | 16-20 | 0 | 0 | 3 | control VS cKO7  p=NaN  ns |
| cKO7 |  | 0 | 0 | 3 |  |
| control | 21-30 | 0 | 0 | 3 | control VS cKO7  p=NaN  ns |
| cKO7 |  | 0 | 0 | 3 |  |
| control | +30 | 0 | 0 | 3 | control VS cKO7  p=0.286  ns |
| cKO7 |  | 0.39 | 0.4 | 3 |  |

Mixed Model ANOVA with Bonferroni post-hoc test (P=0.009)

| **Genotype** | **# axons per Remak Bundle** | **Mean** | **SEM** | **n** | **Comparisons made**  **p-value** |
| --- | --- | --- | --- | --- | --- |
| control | 2-5 | 25.12 | 2 | 3 | control VS dKO  p=0.775  ns |
| dKO |  | 26.16 | 3 | 3 |  |
| control | 6-10 | 22.92 | 3 | 3 | control VS dKO  p=0.255  ns |
| dKO |  | 28.05 | 3 | 3 |  |
| control | 11-15 | 15.94 | 1 | 3 | control VS dKO  p=0.259  ns |
| dKO |  | 19.57 | 3 | 3 |  |
| control | 16-20 | 13.92 | 2 | 3 | control VS dKO  p=0.722  ns |
| dKO |  | 12.89 | 2 | 3 |  |
| control | 21-30 | 16.00 | 3 | 3 | control VS dKO  p=0.330  ns |
| dKO |  | 11.02 | 4 | 3 |  |
| control | +30 | 6.10 | 2 | 3 | control VS dKO  p=0.157  ns |
| dKO |  | 2.32 | 1 | 3 |  |

Mixed Model ANOVA with Bonferroni post-hoc test (P=0.338)

| **Genotype** | **# axons per pocket** | **Mean** | **SEM** | **n** | **Comparisons made**  **p-value** |
| --- | --- | --- | --- | --- | --- |
| control | single | 25.12 | 2 | 3 | control VS dKO  p=0.776  ns |
| dKO |  | 26.16 | 3 | 3 |  |
| control | 2-5 | 22.92 | 3 | 3 | control VS dKO  p=0.255  ns |
| dKO |  | 28.05 | 3 | 3 |  |
| control | 6-10 | 15.94 | 0.5 | 3 | control VS dKO  p=0.259  ns |
| dKO |  | 19.57 | 3 | 3 |  |
| control | 11-15 | 13.92 | 2 | 3 | control VS dKO  p=0.722  ns |
| dKO |  | 12.89 | 2 | 3 |  |
| control | 16-20 | 16.00 | 3 | 3 | control VS dKO  p=0.330  ns |
| dKO |  | 11.02 | 4 | 3 |  |
| control | 21-30 | 6.10 | 2 | 3 | control VS dKO  p=0.157  ns |
| dKO |  | 2.32 | 0.6 | 3 |  |
| control | +30 | 0 | 0 | 3 | control VS dKO  p=NaN  ns |
| dKO |  | 0 | 0 | 3 |  |

Mixed Model ANOVA with Bonferroni post-hoc test (P=0.333)

Figure 2 – figure supplement 1B – qPCR tKO normalize to control *development*

| **Genotype** | **Gen** | **Mean** | **SEM** | **n** | **Comparisons made**  **p-value** |
| --- | --- | --- | --- | --- | --- |
| P2 | *Tyrp1* | 1879.65 | 197.67 | 5 | p<0.0001 *** |
| P8 |  | 12927.94 | 691.51 | 4 | p<0.0001 *** |
| P21 |  | 28013.17 | 4347.82 | 4 | p=0.0007 *** |

Unpaired t test

| **Genotype** | **Gen** | **Mean** | **SEM** | **n** | **Comparisons made**  **p-value** |
| --- | --- | --- | --- | --- | --- |
| P2 | *Mcam* | 270.62 | 30.37 | 5 | p=0.0005 *** |
| P8 |  | 380.80 | 20.35 | 4 | p<0.0001 *** |
| P21 |  | 623.21 | 60.19 | 4 | p=0.0001 *** |

Unpaired t test

| **Genotype** | **Gen** | **Mean** | **SEM** | **n** | **Comparisons made**  **p-value** |
| --- | --- | --- | --- | --- | --- |
| P2 | *Mitf* | 126.81 | 10.31 | 5 | p=0.0297 * |
| P8 |  | 198.78 | 7.68 | 4 | p<0.0001 *** |
| P21 |  | 228.74 | 28.43 | 4 | p=0.0040 ** |

Unpaired t test

| **Genotype** | **Gen** | **Mean** | **SEM** | **n** | **Comparisons made**  **p-value** |
| --- | --- | --- | --- | --- | --- |
| P2 | *Ednrb* | 448.24 | 36.45 | 5 | p<0.0001 *** |
| P8 |  | 574.42 | 22.27 | 4 | p<0.0001 *** |
| P21 |  | 1108.65 | 219.61 | 4 | p=0.0037 ** |

Unpaired t test

Figure 2 – figure supplement 1C – WB tKO P8

| **Genotype** | **Gen** | **Mean** | **SEM** | **n** | **Comparisons made**  **p-value** |
| --- | --- | --- | --- | --- | --- |
| WT | *Mcam* | 0.981 | 0.029 | 4 | WT VS control  P= 0.6549  ns |
| control |  | 0.8302 | 0.034 | 4 | WT VS tKO  p = 0.0001  *** |
| tKO |  | 2.94 | 0.159 | 4 | control VS tKO  p = 0.0001  **** |

ANOVA and Tukey’s multiple comparisons

Figure 2 – figure supplement 1D – WB tKO normalize to Iris

| **Genotype** | **Gen** | **Mean** | **SEM** | **n** | **Comparisons made**  **p-value** |
| --- | --- | --- | --- | --- | --- |
| WT | *Tyrp1* | 0.04 | 0.01 | 3 | WT VS control p = 0.6549 ns |
| control |  | 0.06 | 0.02 | 3 | WT VS tKO p = 0.3774 ns |
| tKO |  | 0.01 | 0.004 | 3 | control VS tKO p= 0.1225 ns |

ANOVA and Bonferroni´s multiple comparisons (P = 0.4851)

**Figure 4 – figure supplement 1**

Figure 4 – figure supplement 1B – TEM Nerve area (µ^2^)

| **Genotype** | **Time** | **Mean** | **SEM** | **n** | **Comparisons made**  **p-value** |
| --- | --- | --- | --- | --- | --- |
| control | UI | 163055 | 3809 | 4 | control VS cKO4 UI  p=0.8936  ns |
| cKO4 |  | 164193 | 7212 | 4 |  |
| control | 10dpi | 209148 | 7294 | 5 | control VS cKO4 10dpi  p=0.0372  * |
| cKO4 |  | 232561 | 6377 | 7 |  |
| control | 20dpi | 194551 | 8453 | 5 | control VS cKO4 20dpi  p=0.6455  ns |
| cKO4 |  | 199290 | 5185 | 5 |  |
| control | 30dpi | 175697 | 4728 | 5 | control VS cKO4 30dpi  p=0.455  ns |
| cKO4 |  | 168893 | 7677 | 4 |  |

Unpaired t test

Figure 4 – figure supplement 1C – TEM Myelinated axons

| **Genotype** | **Time** | **Mean** | **SEM** | **n** | **Comparisons made**  **p-value** |
| --- | --- | --- | --- | --- | --- |
| control | UI | 5855 | 115 | 4 | control VS cKO4 UI  p=0.2340  ns |
| cKO4 |  | 6233 | 261 | 4 |  |
| control | 10dpi | 1113 | 156 | 5 | control VS cKO4 10dpi  P=0.1526  ns |
| cKO4 |  | 807 | 125 | 7 |  |
| control | 20dpi | 5244 | 96 | 5 | control VS cKO4 20dpi  p=0.1738  Ns |
| cKO4 |  | 5417 | 65 | 5 |  |
| control | 30dpi | 5731 | 150 | 5 | control VS cKO4 30dpi  p=0.6440  ns |
| cKO4 |  | 5825 | 109 | 4 |  |

Unpaired t test

Figure 4 – figure supplement 1D – TEM g-ratio

| **Genotype** | **Time** | **Mean** | **SEM** | **n** | **Comparisons made**  **p-value** |
| --- | --- | --- | --- | --- | --- |
| control | UI | 0.68 | 0.003 | 4 | control VS cKO4 UI  p=0.4728  ns |
| cKO4 |  | 0.67 | 0.009 | 4 |  |
| control | 10dpi | 0.98 | 0.004 | 4 | control VS cKO4 10dpi  p=0.7437  ns |
| cKO4 |  | 0.98 | 0.004 | 4 |  |
| control | 20dpi | 0.76 | 0.003 | 5 | control VS cKO4 20dpi  p=0.1554  ns |
| cKO4 |  | 0.77 | 0.007 | 5 |  |
| control | 30dpi | 0.75 | 0.005 | 5 | control VS cKO4 30dpi  p=0.5690  ns |
| cKO4 |  | 0.75 | 0.005 | 4 |  |

Unpaired t test

Figure 4 – figure supplement 1E – TEM Unmyelinated axons number >1.5µm

| **Genotype** | **Time** | **Mean** | **SEM** | **n** | **Comparisons made**  **p-value** |
| --- | --- | --- | --- | --- | --- |
| control | UI | 21 | 8 | 4 | control VS cKO4 UI  p=0.5248  ns |
| cKO4 |  | 30 | 10 | 4 |  |
| control | 10dpi | 1730 | 175 | 5 | control VS cKO4 10dpi  p=0.0236  * |
| cKO4 |  | 2266 | 116 | 7 |  |
| control | 20dpi | 149 | 18 | 4 | control VS cKO4 20dpi  p=0.3500  ns |
| cKO4 |  | 179 | 24 | 4 |  |
| control | 30dpi | 62 | 16 | 5 | control VS cKO4 30dpi  p=0.4853  ns |
| cKO4 |  | 82 | 21 | 4 |  |

Unpaired t test

Figure 4 – figure supplement 1F – TEM Unmyelinated >1.5µm axons in 1:1 relationship

| **Genotype** | **Time** | **Mean** | **SEM** | **n** | **Comparisons made**  **p-value** |
| --- | --- | --- | --- | --- | --- |
| control | UI | 8 | 2 | 4 | control VS cKO4 UI  p=0.0216  * |
| cKO4 |  | 2 | 1 | 4 |  |
| control | 10dpi | 1472 | 130 | 5 | control VS cKO4 10dpi  p=0.0465  * |
| cKO4 |  | 1869 | 115 | 7 |  |
| control | 20dpi | 105 | 10 | 5 | control VS cKO4 20dpi  p=0.6500  ns |
| cKO4 |  | 114 | 16 | 5 |  |
| control | 30dpi | 38 | 9 | 5 | control VS cKO4 30dpi  p=0.2922  ns |
| cKO4 |  | 58 | 16 | 4 |  |

Unpaired t test

Figure 4 – figure supplement 1G – TEM Total axon number >1.5µm

| **Genotype** | **Time** | **Mean** | **SEM** | **n** | **Comparisons made**  **p-value** |
| --- | --- | --- | --- | --- | --- |
| control | UI | 5877 | 121 | 4 | control VS cKO4 UI  p=0.2310  ns |
| cKO4 |  | 6263 | 263 | 4 |  |
| control | 10dpi | 2844 | 103 | 5 | control VS cKO4 10dpi  p=0.3984  ns |
| cKO4 |  | 3073 | 204 | 7 |  |
| control | 20dpi | 5426 | 111 | 5 | control VS cKO4 20dpi  p=0.1521  ns |
| cKO4 |  | 5626 | 60 | 5 |  |
| control | 30dpi | 5794 | 163 | 5 | control VS cKO4 30dpi  p=0.6157  ns |
| cKO4 |  | 5908 | 128 | 4 |  |

Unpaired t test

Figure 4 – figure supplement 1H – TEM Total axon number >1.5µm axons in 1:1 relationship

| **Genotype** | **Time** | **Mean** | **SEM** | **n** | **Comparisons made**  **p-value** |
| --- | --- | --- | --- | --- | --- |
| control | UI | 5863 | 115 | 4 | control VS cKO4 UI  p=0.2401  ns |
| cKO4 |  | 6235 | 261 | 4 |  |
| control | 10dpi | 2585 | 86 | 5 | control VS cKO4 10dpi  p=0.7313  ns |
| cKO4 |  | 2676 | 204 | 7 |  |
| control | 20dpi | 5368 | 102 | 5 | control VS cKO4 20dpi  p=0.1378  ns |
| cKO4 |  | 5558 | 54 | 5 |  |
| control | 30dpi | 5769 | 155 | 5 | control VS cKO4 30dpi  p=0.5944  ns |
| cKO4 |  | 5884 | 122 | 4 |  |

Unpaired t test

Figure 4 – figure supplement 1I – TEM Schwann cell nuclei

| **Genotype** | **Time** | **Mean** | **SEM** | **n** | **Comparisons made**  **p-value** |
| --- | --- | --- | --- | --- | --- |
| control | UI | 221 | 10 | 4 | control VS cKO4 UI  p=0.0592  ns |
| cKO4 |  | 283 | 25 | 4 |  |
| control | 10dpi | 707 | 72 | 5 | control VS cKO4 10dpi  p=0.3883  ns |
| cKO4 |  | 789 | 57 | 7 |  |
| control | 20dpi | 643 | 41 | 5 | control VS cKO4 20dpi  p=0.0410  * |
| cKO4 |  | 765 | 29 | 5 |  |
| control | 30dpi | 575 | 32 | 5 | control VS cKO4 30dpi  p=0.0066  ** |
| cKO4 |  | 752 | 34 | 4 |  |

Unpaired t test

Figure 4 – figure supplement 1J – TEM Myelinating Schwann cell nuclei

| **Genotype** | **Time** | **Mean** | **SEM** | **n** | **Comparisons made**  **p-value** |
| --- | --- | --- | --- | --- | --- |
| control | UI | 163 | 7 | 4 | control VS cKO4 UI  p=0.0527  ns |
| cKO4 |  | 197 | 12 | 4 |  |
| control | 10dpi | 96 | 12 | 5 | control VS cKO4 10dpi  p=0.0866  ns |
| cKO4 |  | 66 | 10 | 7 |  |
| control | 20dpi | 319 | 14 | 5 | control VS cKO4 20dpi  p=0.0451  * |
| cKO4 |  | 368 | 15 | 5 |  |
| control | 30dpi | 318 | 19 | 5 | control VS cKO4 30dpi  p=0.1113  ns |
| cKO4 |  | 378 | 28 | 4 |  |

Unpaired t test

Figure 4 – figure supplement 1K – % Axons >1.5µm

| **Genotype** | **Time** | **Mean** | **SEM** | **n** | **Comparisons made**  **p-value** |
| --- | --- | --- | --- | --- | --- |
| control | UI | 99.87 | 0.03 | 4 | control VS cKO4 UI  p=0.5003  ns |
| cKO4 |  | 99.97 | 0.02 | 4 |  |
| control | 10dpi | 42.83 | 5.42 | 5 | control VS cKO4 10dpi  p=0.0474  * |
| cKO4 |  | 28.96 | 3.21 | 6 |  |
| control | 20dpi | 97.71 | 0.36 | 5 | control VS cKO4 20dpi  p=0.7046  ns |
| cKO4 |  | 97.45 | 0.56 | 5 |  |
| control | 30dpi | 99.03 | 0.26 | 4 | control VS cKO4 30dpi  p=0.3007  ns |
| cKO4 |  | 99.34 | 0.14 | 5 |  |

Unpaired t test

Figure 4 – figure supplement 1L – Western Blot 10 y 21 dpi cKO4 J

| **Genotype** | **Time** | **Mean** | **SEM** | **n** | **Comparisons made**  **p-value** |
| --- | --- | --- | --- | --- | --- |
| UI | - | 0.22 | 0.03 | 4 | N / A |
| control | 10dpi | 1 | 0.05 | 6 | control VS cKO4 10dpi  p=0.0009  *** |
| cKO4 |  | 1.37 | 0.07 | 6 |  |
| control | 21dpi | 1.03 | 0.05 | 6 | control VS cKO4 20dpi  p=0.0563  ns |
| cKO4 |  | 1.27 | 0.1 | 6 |  |

Unpaired t test

Figure 4 – figure supplement 1L – Western Blot 10 y 21 dpi cKO4 Krox-20

| **Genotype** | **Time** | **Mean** | **SEM** | **n** | **Comparisons made**  **p-value** |
| --- | --- | --- | --- | --- | --- |
| UI | - | 0.59 | 0.09 | 4 | N / A |
| control | 10dpi | 1 | 0.05 | 6 | control VS cKO4 10dpi  p=0.1455  ns |
| cKO4 |  | 0.88 | 0.05 | 6 |  |
| control | 21dpi | 1.15 | 0.07 | 6 | control VS cKO4 20dpi  p=0.8159  ns |
| cKO4 |  | 1.12 | 0.06 | 6 |  |

Unpaired t test

Figure 4 – figure supplement 1L – Western Blot 10 y 21 dpi cKO4 MPZ

| **Genotype** | **Time** | **Mean** | **SEM** | **n** | **Comparisons made**  **p-value** |
| --- | --- | --- | --- | --- | --- |
| UI | - | 1 | 0.05 | 6 | N / A |
| control | 10dpi | 0.11 | 0.004 | 7 | control VS cKO4 10dpi  p=0.2836  ns |
| cKO4 |  | 0.11 | 0.01 | 7 |  |
| control | 21dpi | 0.37 | 0.02 | 9 | control VS cKO4 20dpi  p=0.1703  ns |
| cKO4 |  | 0.33 | 0.03 | 9 |  |

Unpaired t test

Figure 4 – figure supplement 1M – qPCR 10 dpi cKO4 mRNA fold change injured vs uninjured

| **Genotype** | **gen** | **Mean** | **SEM** | **n** | **Comparisons made**  **p-value** |
| --- | --- | --- | --- | --- | --- |
| control | *Jun* | 2.48 | 0.27 | 5 | control VS cKO4  p=0.299  ns |
| cKO4 |  | 2.94 | 0.31 | 6 |  |

Unpaired t test

| **Genotype** | **gen** | **Mean** | **SEM** | **n** | **Comparisons made**  **p-value** |
| --- | --- | --- | --- | --- | --- |
| control | *Runx2* | 14.73 | 1.68 | 4 | control VS cKO4  p=0.0508  ns |
| cKO4 |  | 23.89 | 3.37 | 4 |  |

Unpaired t test

| **Genotype** | **gen** | **Mean** | **SEM** | **n** | **Comparisons made**  **p-value** |
| --- | --- | --- | --- | --- | --- |
| control | *Gdnf* | 37.65 | 5.10 | 5 | control VS cKO4  p=0.0775  ns |
| cKO4 |  | 60.37 | 9.99 | 5 |  |

Unpaired t test

Figure 4 – figure supplement 1N – qPCR 10 dpi cKO4 mRNA fold change injured vs uninjured

| **Genotype** | **gen** | **Mean** | **SEM** | **n** | **Comparisons made**  **p-value** |
| --- | --- | --- | --- | --- | --- |
| control | *Krox-20* | 0.38 | 0.03 | 6 | control VS cKO4  p=0.9181  ns |
| cKO4 |  | 0.38 | 0.06 | 6 |  |

Unpaired t test

| **Genotype** | **gen** | **Mean** | **SEM** | **n** | **Comparisons made**  **p-value** |
| --- | --- | --- | --- | --- | --- |
| control | *Pou3f1* | 10.63 | 1.50 | 4 | control VS cKO4  p=0.5898  ns |
| cKO4 |  | 11.75 | 1.27 | 4 |  |

Unpaired t test

| **Genotype** | **gen** | **Mean** | **SEM** | **n** | **Comparisons made**  **p-value** |
| --- | --- | --- | --- | --- | --- |
| control | *Prx* | 0.29 | 0.02 | 5 | control VS cKO4  p=0.0125  * |
| cKO4 |  | 0.21 | 0.02 | 5 |  |

Unpaired t test

| **Genotype** | **gen** | **Mean** | **SEM** | **n** | **Comparisons made**  **p-value** |
| --- | --- | --- | --- | --- | --- |
| control | *Mpz* | 0.32 | 0.03 | 6 | control VS cKO4  p=0.0157  * |
| cKO4 |  | 0.21 | 0.02 | 6 |  |

Unpaired t test

| **Genotype** | **gen** | **Mean** | **SEM** | **n** | **Comparisons made**  **p-value** |
| --- | --- | --- | --- | --- | --- |
| control | *Mbp* | 0.19 | 0.02 | 5 | control VS cKO4  p=0.0083  ** |
| cKO4 |  | 0.11 | 0.02 | 5 |  |

Unpaired t test

Figure 4 – figure supplement 1O – Number of intact myelin sheaths per nerve after 4 day cut cKO4

| **Genotype** | **Time** | **Mean** | **SEM** | **n** | **Comparisons made**  **p-value** |
| --- | --- | --- | --- | --- | --- |
| control | Cut 4d | 262 | 19 | 6 | control VS cKO4  p=0.3112  ns |
| cKO4 |  | 294 | 23 | 6 |  |

Unpaired t test

**Figure 4 – figure supplement 2**

Figure 4 – figure supplement 2B – TEM Nerve area (µ^2^)

| **Genotype** | **Time** | **Mean** | **SEM** | **n** | **Comparisons made**  **p-value** |
| --- | --- | --- | --- | --- | --- |
| WT | UI | 155108 | 4058 | 4 | WT VS KO5 UI  p=0.7529  ns |
| KO5 |  | 152954 | 5121 | 4 |  |
| WT | 10dpi | 215140 | 7409 | 4 | WT VS KO5 10dpi  p=0.3508  ns |
| KO5 |  | 207334 | 2157 | 4 |  |
| WT | 20dpi | 164863 | 5154 | 4 | WT VS KO5 20dpi  p=0.6077  ns |
| KO5 |  | 160974 | 5002 | 4 |  |
| WT | 30dpi | 152210 | 10235 | 5 | WT VS KO5 30dpi  p=0.3273  ns |
| KO5 |  | 168494 | 9386 | 3 |  |
| WT | 60dpi | 144730 | 4484 | 4 | WT VS KO5 60dpi  p=0.0419  * |
| KO5 |  | 170207 | 9238 | 3 |  |

Unpaired t test

Figure 4 – figure supplement 2C – TEM Myelinated axons

| **Genotype** | **Time** | **Mean** | **SEM** | **n** | **Comparisons made**  **p-value** |
| --- | --- | --- | --- | --- | --- |
| WT | UI | 6088 | 120 | 4 | WT VS KO5 UI  p=0.7544  ns |
| KO5 |  | 6170 | 223 | 4 |  |
| WT | 10dpi | 2038 | 97 | 4 | WT VS KO5 10dpi  p=0.3329  ns |
| KO5 |  | 2215 | 137 | 4 |  |
| WT | 20dpi | 5144 | 102 | 4 | WT VS KO5 20dpi  p=0.9260  ns |
| KO5 |  | 5159 | 106 | 4 |  |
| WT | 30dpi | 5850 | 266 | 5 | WT VS KO5 30dpi  p=0.8080  ns |
| KO5 |  | 5945 | 165 | 3 |  |
| WT | 60dpi | 6277 | 162 | 4 | WT VS KO5 60dpi  p=0.5952  ns |
| KO5 |  | 6402 | 132 | 3 |  |

Unpaired t test

Figure 4 – figure supplement 2D – TEM g-ratio

| **Genotype** | **Time** | **Mean** | **SEM** | **n** | **Comparisons made**  **p-value** |
| --- | --- | --- | --- | --- | --- |
| WT | UI | 0.68 | 0.006 | 4 | WT VS KO5 UI  p=0.3266  ns |
| KO5 |  | 0.69 | 0.006 | 4 |  |
| WT | 10dpi | 0.96 | 0.002 | 4 | WT VS KO5 10dpi  p=0.7049  ns |
| KO5 |  | 0.96 | 0.008 | 4 |  |
| WT | 20dpi | 0.78 | 0.007 | 4 | WT VS KO5 20dpi  p=0.0154  * |
| KO5 |  | 0.75 | 0.004 | 4 |  |
| WT | 30dpi | 0.75 | 0.005 | 5 | WT VS KO5 30dpi  p=0.7991  ns |
| KO5 |  | 0.75 | 0.005 | 3 |  |
| WT | 60dpi | 0.75 | 0.001 | 3 | WT VS KO5 60dpi  p=0.5406  ns |
| KO5 |  | 0.76 | 0.005 | 3 |  |

Unpaired t test

Figure 4 – figure supplement 2E – TEM Unmyelinated axons number >1.5µm

| **Genotype** | **Time** | **Mean** | **SEM** | **n** | **Comparisons made**  **p-value** |
| --- | --- | --- | --- | --- | --- |
| WT | UI | 35 | 9 | 4 | WT VS KO5 UI  p=0.5226  ns |
| KO5 |  | 47 | 15 | 4 |  |
| WT | 10dpi | 1194 | 155 | 4 | WT VS KO5 10dpi  p=0.4499  ns |
| KO5 |  | 1326 | 51 | 4 |  |
| WT | 20dpi | 144 | 16 | 4 | WT VS KO5 20dpi  p=0.6371  ns |
| KO5 |  | 133 | 14 | 4 |  |
| WT | 30dpi | 51 | 16 | 5 | WT VS KO5 30dpi  p=0.7079  ns |
| KO5 |  | 42 | 12 | 3 |  |
| WT | 60dpi | 22 | 6 | 4 | WT VS KO5 60dpi  p=0.0352  * |
| KO5 |  | 52 | 10 | 3 |  |

Unpaired t test

Figure 4 – figure supplement 2F – TEM Unmyelinated >1.5µm axons in 1:1 relationship

| **Genotype** | **Time** | **Mean** | **SEM** | **n** | **Comparisons made**  **p-value** |
| --- | --- | --- | --- | --- | --- |
| WT | UI | 4 | 2 | 4 | WT VS KO5 UI  p=0.9240  ns |
| KO5 |  | 5 | 2 | 4 |  |
| WT | 10dpi | 1088 | 131 | 4 | WT VS KO5 10dpi  p=0.8036  ns |
| KO5 |  | 1123 | 42 | 4 |  |
| WT | 20dpi | 109 | 14 | 4 | WT VS KO5 20dpi  p=0.1374  ns |
| KO5 |  | 82 | 7 | 4 |  |
| WT | 30dpi | 22 | 7 | 5 | WT VS KO5 30dpi  p=0.7025  ns |
| KO5 |  | 27 | 12 | 3 |  |
| WT | 60dpi | 11 | 3 | 4 | WT VS KO5 60dpi  p=0.2749  ns |
| KO5 |  | 20 | 8 | 3 |  |

Unpaired t test

Figure 4 – figure supplement 2G – TEM Total axon number >1.5µm

| **Genotype** | **Time** | **Mean** | **SEM** | **n** | **Comparisons made**  **p-value** |
| --- | --- | --- | --- | --- | --- |
| WT | UI | 6123 | 113 | 4 | WT VS KO5 UI  p=0.7190  ns |
| KO5 |  | 6217 | 224 | 4 |  |
| WT | 10dpi | 3232 | 147 | 4 | WT VS KO5 10dpi  p=0.2213  ns |
| KO5 |  | 3541 | 171 | 4 |  |
| WT | 20dpi | 5288 | 115 | 4 | WT VS KO5 20dpi  p=0.9800  ns |
| KO5 |  | 5292 | 115 | 4 |  |
| WT | 30dpi | 5901 | 268 | 5 | WT VS KO5 30dpi  p=0.8248  ns |
| KO5 |  | 5987 | 155 | 3 |  |
| WT | 60dpi | 6298 | 167 | 4 | WT VS KO5 60dpi  p=0.5207  ns |
| KO5 |  | 6454 | 132 | 3 |  |

Unpaired t test

Figure 4 – figure supplement 2H – TEM Total axon number >1.5µm axons in 1:1 relationship

| **Genotype** | **Time** | **Mean** | **SEM** | **n** | **Comparisons made**  **p-value** |
| --- | --- | --- | --- | --- | --- |
| WT | UI | 6092 | 119 | 4 | WT VS KO5 UI  p=0.7550  ns |
| KO5 |  | 6175 | 224 | 4 |  |
| WT | 10dpi | 3125 | 132 | 4 | WT VS KO5 10dpi  p=0.2925  ns |
| KO5 |  | 3338 | 128 | 4 |  |
| WT | 20dpi | 5253 | 108 | 4 | WT VS KO5 20dpi  p=0.9392  ns |
| KO5 |  | 5241 | 110 | 4 |  |
| WT | 30dpi | 5872 | 269 | 5 | WT VS KO5 30dpi  p=0.7982  ns |
| KO5 |  | 5973 | 155 | 3 |  |
| WT | 60dpi | 6288 | 164 | 4 | WT VS KO5 60dpi  p=0.5718  ns |
| KO5 |  | 6422 | 126 | 3 |  |

Unpaired t test

Figure 4 – figure supplement 2I – TEM Schwann cell nuclei

| **Genotype** | **Time** | **Mean** | **SEM** | **n** | **Comparisons made**  **p-value** |
| --- | --- | --- | --- | --- | --- |
| WT | UI | 254 | 10 | 4 | WT VS KO5 UI  p=0.6594  ns |
| KO5 |  | 244 | 19 | 4 |  |
| WT | 10dpi | 598 | 18 | 4 | WT VS KO5 10dpi  p=0.4526  ns |
| KO5 |  | 632 | 38 | 4 |  |
| WT | 20dpi | 683 | 28 | 4 | WT VS KO5 20dpi  p=0.2068  ns |
| KO5 |  | 618 | 36 | 4 |  |
| WT | 30dpi | 630 | 51 | 5 | WT VS KO5 30dpi  p=0.9539  ns |
| KO5 |  | 626 | 55 | 3 |  |
| WT | 60dpi | 611 | 25 | 4 | WT VS KO5 60dpi  p=0.3009  ns |
| KO5 |  | 644 | 5 | 3 |  |

Unpaired t test

Figure 4 – figure supplement 2J – TEM Myelinating Schwann cell nuclei

| **Genotype** | **Time** | **Mean** | **SEM** | **n** | **Comparisons made**  **p-value** |
| --- | --- | --- | --- | --- | --- |
| WT | UI | 183 | 17 | 4 | WT VS KO5 UI  p=0.9504  ns |
| KO5 |  | 185 | 16 | 4 |  |
| WT | 10dpi | 172 | 14 | 4 | WT VS KO5 10dpi  p=0.6694  ns |
| KO5 |  | 186 | 27 | 4 |  |
| WT | 20dpi | 291 | 11 | 4 | WT VS KO5 20dpi  p=0.2612  ns |
| KO5 |  | 262 | 21 | 4 |  |
| WT | 30dpi | 333 | 22 | 5 | WT VS KO5 30dpi  p=0.3739  ns |
| KO5 |  | 302 | 19 | 3 |  |
| WT | 60dpi | 320 | 8 | 4 | WT VS KO5 60dpi  p=0.1063  ns |
| KO5 |  | 343 | 8 | 3 |  |

Unpaired t test

Figure 4 – figure supplement 2K – % Axons >1.5µm

| **Genotype** | **Time** | **Mean** | **SEM** | **n** | **Comparisons made**  **p-value** |
| --- | --- | --- | --- | --- | --- |
| WT | UI | 99.93 | 0.03 | 4 | WT VS KO5 UI  p=0.0736  ns |
| KO5 |  | 99.93 | 0.03 | 4 |  |
| WT | 10dpi | 65.43 | 3.33 | 4 | WT VS KO5 10dpi  p=0.8453  ns |
| KO5 |  | 66.20 | 1.76 | 4 |  |
| WT | 20dpi | 97.94 | 0.25 | 4 | WT VS KO5 20dpi  p=0.1178  ns |
| KO5 |  | 98.44 | 0.11 | 4 |  |
| WT | 30dpi | 99.62 | 0.11 | 5 | WT VS KO5 30dpi  p=0.7171  ns |
| KO5 |  | 99.54 | 0.22 | 3 |  |
| WT | 60dpi | 99.83 | 0.04 | 4 | WT VS KO5 60dpi  p=0.2602  ns |
| KO5 |  | 99.68 | 0.12 | 3 |  |

Unpaired t test & Unpaired t test with Welch´s correlation

Figure 4 – figure supplement 2L – qPCR 10 dpi KO5 mRNA fold change injured vs uninjured

| **Genotype** | **gen** | **Mean** | **SEM** | **n** | **Comparisons made**  **p-value** |
| --- | --- | --- | --- | --- | --- |
| WT | *Jun* | 2.85 | 0.30 | 8 | WT VS KO5  p=0.7033  ns |
| KO5 |  | 3.02 | 0.32 | 8 |  |

Unpaired t test

| **Genotype** | **gen** | **Mean** | **SEM** | **n** | **Comparisons made**  **p-value** |
| --- | --- | --- | --- | --- | --- |
| WT | *Runx2* | 17.86 | 4.05 | 7 | WT VS KO5  p=0.7202  ns |
| KO5 |  | 16.04 | 2.49 | 6 |  |

Unpaired t test

| **Genotype** | **gen** | **Mean** | **SEM** | **n** | **Comparisons made**  **p-value** |
| --- | --- | --- | --- | --- | --- |
| WT | *Gdnf* | 29.90 | 2.72 | 6 | WT VS KO5  p=0.1958  ns |
| KO5 |  | 34.44 | 1.82 | 6 |  |

Unpaired t test

Figure 4 – figure supplement 2M – qPCR 10 dpi KO5 mRNA fold change injured vs uninjured

| **Genotype** | **gen** | **Mean** | **SEM** | **n** | **Comparisons made**  **p-value** |
| --- | --- | --- | --- | --- | --- |
| WT | *Krox-20* | 0.66 | 0.10 | 8 | WT VS KO5  p=0.2241  ns |
| KO5 |  | 0.51 | 0.06 | 8 |  |

Unpaired t test

| **Genotype** | **gen** | **Mean** | **SEM** | **n** | **Comparisons made**  **p-value** |
| --- | --- | --- | --- | --- | --- |
| WT | *Pou3f1* | 12.64 | 1.14 | 8 | WT VS KO5  p=0.1121  ns |
| KO5 |  | 9.61 | 1.38 | 8 |  |

Unpaired t test

| **Genotype** | **gen** | **Mean** | **SEM** | **n** | **Comparisons made**  **p-value** |
| --- | --- | --- | --- | --- | --- |
| WT | *Prx* | 0.37 | 0.03 | 5 | WT VS KO5  p=0.0774  ns |
| KO5 |  | 0.30 | 0.02 | 6 |  |

Unpaired t test

| **Genotype** | **gen** | **Mean** | **SEM** | **n** | **Comparisons made**  **p-value** |
| --- | --- | --- | --- | --- | --- |
| WT | *Mpz* | 0.38 | 0.04 | 8 | WT VS KO5  p=0.3339  ns |
| KO5 |  | 0.33 | 0.03 | 8 |  |

Unpaired t test

| **Genotype** | **gen** | **Mean** | **SEM** | **n** | **Comparisons made**  **p-value** |
| --- | --- | --- | --- | --- | --- |
| WT | *Mbp* | 0.30 | 0.02 | 8 | WT VS KO5  p=0.0071  ** |
| KO5 |  | 0.21 | 0.02 | 8 |  |

Unpaired t test

Figure 4 – figure supplement 2N – Number of intact myelin sheaths per nerve after 4 day cut KO5

| **Genotype** | **Time** | **Mean** | **SEM** | **n** | **Comparisons made**  **p-value** |
| --- | --- | --- | --- | --- | --- |
| WT | Cut 4d | 368 | 9 | 4 | WT VS KO5  p=0.0717  ns |
| KO5 |  | 337 | 11 | 4 |  |

Unpaired t test

**Figure 4 – figure supplement 3**

Figure 4 – figure supplement 3A – Number of intact myelin sheaths per nerve after 4 day cut dKO

| **Genotype** | **Time** | **Mean** | **SEM** | **n** | **Comparisons made**  **p-value** |
| --- | --- | --- | --- | --- | --- |
| control | Cut 4d | 352 | 24 | 5 | control VS dKO  p=0.9413  ns |
| dKO |  | 349 | 31 | 4 |  |

Unpaired t test

Figure 4 – figure supplement 3B – Western Blot Myelin clearance dKO: JUN

| **Genotype** | **Time** | **Mean** | **SEM** | **n** | **Comparisons made**  **p-value** |
| --- | --- | --- | --- | --- | --- |
| WT | UI | 0.13 | 0.008 | 3 | ANOVA  p=0.1694  ns |
| control |  | 0.12 | 0.007 | 3 |  |
| dKO |  | 0.15 | 0.003 | 3 |  |
| WT | 4 d | 0.93 | 0.059 | 3 | ANOVA  p=0.5977  ns |
| control |  | 0.89 | 0.114 | 3 |  |
| dKO |  | 1 | 0.006 | 3 |  |
| WT | 7 d | 1 | 0 | 3 | ANOVA  p=0.1111  ns |
| control |  | 1.32 | 0.094 | 3 |  |
| dKO |  | 1.31 | 1.48 | 3 |  |
| WT | 10 d | 0.9 | 0.033 | 3 | ANOVA  p=0.7220  ns |
| control |  | 0.92 | 0.114 | 3 |  |
| dKO |  | 0.81 | 0.146 | 3 |  |

ANOVA

Figure 4 – figure supplement 3B – Western Blot Myelin clearance dKO: MPZ

| **Genotype** | **Time** | **Mean** | **SEM** | **n** | **Comparisons made**  **p-value** |
| --- | --- | --- | --- | --- | --- |
| WT | UI | 1 | 0 | 3 | ANOVA  p=0.1900  ns |
| control |  | 1.16 | 0.2 | 3 |  |
| dKO |  | 1.4 | 0.11 | 3 |  |
| WT | 4 d | 0.71 | 0.06 | 3 | ANOVA  p=0.6068  ns |
| control |  | 0.87 | 0.19 | 3 |  |
| dKO |  | 0.96 | 0.21 | 3 |  |
| WT | 7 d | 0.3 | 0.05 | 3 | ANOVA  p=0.2341  ns |
| control |  | 0.45 | 0.06 | 3 |  |
| dKO |  | 0.32 | 0.08 | 3 |  |
| WT | 10 d | 0.16 | 0.02 | 3 | ANOVA  p=0.1983  ns |
| control |  | 0.18 | 0.02 | 3 |  |
| dKO |  | 0.13 | 0.02 | 3 |  |

ANOVA

Figure 4 – figure supplement 3C – qPCR Repair cells *Gdnf*

| **Genotype** | **Time** | **Mean** | **SEM** | **n** | **Comparisons made**  **p-value** |
| --- | --- | --- | --- | --- | --- |
| control | 1d | 355.74 | 84.60 | 4 | control VS dKO 1d  p=0.6085  ns |
| dKO |  | 414.35 | 67.93 | 4 |  |
| control | 4d | 265.45 | 20.52 | 4 | control VS dKO 4d  p=0.8736  ns |
| dKO |  | 274.15 | 54.92 | 3 |  |
| control | 7d | 257.30 | 39.63 | 4 | control VS dKO 7d  p=0.5347  ns |
| dKO |  | 291.56 | 33.71 | 4 |  |

Unpaired t test

Figure 4 – figure supplement 3C – qPCR Repair cells *Olig1*

| **Genotype** | **Time** | **Mean** | **SEM** | **n** | **Comparisons made**  **p-value** |
| --- | --- | --- | --- | --- | --- |
| control | 1d | 0.85 | 0.30 | 4 | control VS dKO 1d  p=0.6340  ns |
| dKO |  | 1.11 | 0.43 | 4 |  |
| control | 4d | 30.43 | 3.81 | 4 | control VS dKO 4d  p=0.1588  ns |
| dKO |  | 22.82 | 1.07 | 3 |  |
| control | 7d | 241.33 | 19.15 | 4 | control VS dKO 7d  p=0.6912  ns |
| dKO |  | 255.14 | 27.02 | 4 |  |

Unpaired t test

Figure 4 – figure supplement 3C – qPCR Repair cells *Shh*

| **Genotype** | **Time** | **Mean** | **SEM** | **n** | **Comparisons made**  **p-value** |
| --- | --- | --- | --- | --- | --- |
| control | 1d | 176.91 | 40.33 | 4 | control VS dKO 1d  p=0.7497  ns |
| dKO |  | 190.55 | 6.54 | 4 |  |
| control | 4d | 166.73 | 50.44 | 4 | control VS dKO 4d  p=0.6056  ns |
| dKO |  | 129.90 | 36.12 | 3 |  |
| control | 7d | 314.30 | 59.06 | 4 | control VS dKO 7d  p=0.6191  ns |
| dKO |  | 276.98 | 39.82 | 4 |  |

Unpaired t test

Figure 4 – figure supplement 3C – qPCR Repair cells *Jun*

| **Genotype** | **Time** | **Mean** | **SEM** | **n** | **Comparisons made**  **p-value** |
| --- | --- | --- | --- | --- | --- |
| control | 1d | 2.35 | 0.22 | 4 | control VS dKO 1d  p=0.0102  * |
| dKO |  | 3.71 | 0.29 | 4 |  |
| control | 4d | 5.49 | 0.80 | 4 | control VS dKO 4d  p=0.2569  ns |
| dKO |  | 4.12 | 0.59 | 3 |  |
| control | 7d | 6.87 | 0.98 | 4 | control VS dKO 7d  p=0.6854  ns |
| dKO |  | 7.38 | 0.70 | 4 |  |

Unpaired t test

Figure 4 – figure supplement 3C – qPCR Repair cells *Ngfr*

| **Genotype** | **Time** | **Mean** | **SEM** | **n** | **Comparisons made**  **p-value** |
| --- | --- | --- | --- | --- | --- |
| control | 1d | 1.78 | 0.08 | 4 | control VS dKO 1d  p=0.8782  ns |
| dKO |  | 1.75 | 0.16 | 3 |  |
| control | 4d | 15.88 | 2.14 | 4 | control VS dKO 4d  p=0.0545  ns |
| dKO |  | 9.22 | 0.96 | 3 |  |
| control | 7d | 24.72 | 5.93 | 4 | control VS dKO 7d  p=0.2129  ns |
| dKO |  | 15.58 | 2.81 | 4 |  |

Unpaired t test

Figure 4 – figure supplement 3C – qPCR Repair cells *Mpz*

| **Genotype** | **Time** | **Mean** | **SEM** | **n** | **Comparisons made**  **p-value** |
| --- | --- | --- | --- | --- | --- |
| control | 1d | 0.29 | 0.03 | 4 | control VS dKO 1d  p=0.2090  ns |
| dKO |  | 0.39 | 0.06 | 4 |  |
| control | 4d | 0.0024 | 0.0006 | 4 | control VS dKO 4d  pP=0.5674  ns |
| dKO |  | 0.0020 | 0.0001 | 3 |  |

Unpaired t test

**Figure 5 – figure supplement 1**

Figure 5 – figure supplement 1B – TEM Nerve area (µ^2^)

| **Genotype** | **Time** | **Mean** | **SEM** | **n** | **Comparisons made**  **p-value** |
| --- | --- | --- | --- | --- | --- |
| control | UI | 165776 | 2923 | 5 | control VS cKO7 UI  p=0.7819  ns |
| cKO7 |  | 167925 | 6911 | 5 |  |
| control | 10dpi | 195235 | 10494 | 5 | control VS cKO7 10dpi  p=0.2806  ns |
| cKO7 |  | 208393 | 4375 | 5 |  |
| control | 20dpi | 191607 | 2343 | 5 | control VS cKO7 20dpi  p=0.6779  ns |
| cKO7 |  | 186948 | 10556 | 5 |  |

Unpaired t test

Figure 5 – figure supplement 1C – TEM Myelinated axons

| **Genotype** | **Time** | **Mean** | **SEM** | **n** | **Comparisons made**  **p-value** |
| --- | --- | --- | --- | --- | --- |
| control | UI | 6555 | 134 | 5 | control VS cKO7 UI  pP=0.8477  ns |
| cKO7 |  | 6610 | 244 | 5 |  |
| control | 10dpi | 1680 | 155 | 5 | control VS cKO7 10dpi  p=0.3633  ns |
| cKO7 |  | 1469 | 154 | 5 |  |
| control | 20dpi | 5125 | 39 | 5 | control VS cKO7 20dpi  p=0.1606  ns |
| cKO7 |  | 4902 | 139 | 5 |  |

Unpaired t test

Figure 5 – figure supplement 1D – TEM g-ratio

| **Genotype** | **Time** | **Mean** | **SEM** | **n** | **Comparisons made**  **p-value** |
| --- | --- | --- | --- | --- | --- |
| control | UI | 0.69 | 0.005 | 5 | control VS cKO7 UI  p=0.3609  ns |
| cKO7 |  | 0.70 | 0.007 | 5 |  |
| control | 10dpi | 0.92 | 0.005 | 5 | control VS cKO7 10dpi  p=0.3003  ns |
| cKO7 |  | 0.93 | 0.006 | 5 |  |
| control | 20dpi | 0.75 | 0.006 | 5 | control VS cKO7 20dpi  p=0.5360  ns |
| cKO7 |  | 0.75 | 0.003 | 4 |  |

Unpaired t test

Figure 5 – figure supplement 1E – TEM Unmyelinated axons number >1.5µm

| **Genotype** | **Time** | **Mean** | **SEM** | **n** | **Comparisons made**  **p-value** |
| --- | --- | --- | --- | --- | --- |
| control | UI | 26 | 10 | 5 | control VS cKO7 UI  p=0.8782  ns |
| cKO7 |  | 24 | 6 | 5 |  |
| control | 10dpi | 1578 | 91 | 5 | control VS cKO7 10dpi  p=0.2347  ns |
| cKO7 |  | 1901 | 234 | 5 |  |
| control | 20dpi | 121 | 17 | 5 | control VS cKO7 20dpi  p=0.0561  ns |
| cKO7 |  | 174 | 17 | 5 |  |

Unpaired t test

Figure 5 – figure supplement 1F – TEM Unmyelinated >1.5µm axons in 1:1 relationship

| **Genotype** | **Time** | **Mean** | **SEM** | **n** | **Comparisons made**  **p-value** |
| --- | --- | --- | --- | --- | --- |
| control | UI | 0 | 0 | 5 | control VS cKO7 UI  p=0.2025  ns |
| cKO7 |  | 10 | 7 | 5 |  |
| control | 10dpi | 1384 | 131 | 5 | control VS cKO7 10dpi  p=0.3585  ns |
| cKO7 |  | 1637 | 224 | 5 |  |
| control | 20dpi | 86 | 10 | 5 | control VS cKO7 20dpi  p=0.1000  ns |
| cKO7 |  | 114 | 11 | 5 |  |

Unpaired t test

Figure 5 – figure supplement 1G – TEM Total axon number >1.5µm

| **Genotype** | **Time** | **Mean** | **SEM** | **n** | **Comparisons made**  **p-value** |
| --- | --- | --- | --- | --- | --- |
| control | UI | 6581 | 136 | 5 | control VS cKO7 UI  p=0.8514  ns |
| cKO7 |  | 6635 | 245 | 5 |  |
| control | 10dpi | 3258 | 241 | 5 | control VS cKO7 10dpi  p=0.7449  ns |
| cKO7 |  | 3369 | 225 | 5 |  |
| control | 20dpi | 5246 | 45 | 5 | control VS cKO7 20dpi  p=0.3158  ns |
| cKO7 |  | 5076 | 152 | 5 |  |

Unpaired t test

Figure 5 – figure supplement 1H – TEM Total axon number >1.5µm axons in 1:1 relationship

| **Genotype** | **Time** | **Mean** | **SEM** | **n** | **Comparisons made**  **p-value** |
| --- | --- | --- | --- | --- | --- |
| control | UI | 6555 | 134 | 5 | control VS cKO7 UI  p=0.8190  ns |
| cKO7 |  | 6620 | 241 | 5 |  |
| control | 10dpi | 3065 | 276 | 5 | control VS cKO7 10dpi  p=0.9133  ns |
| cKO7 |  | 3105 | 233 | 5 |  |
| control | 20dpi | 5212 | 40 | 5 | control VS cKO7 20dpi  p=0.2224  ns |
| cKO7 |  | 5015 | 143 | 5 |  |

Unpaired t test

Figure 5 – figure supplement 1I – TEM Schwann cell nuclei

| **Genotype** | **Time** | **Mean** | **SEM** | **n** | **Comparisons made**  **p-value** |
| --- | --- | --- | --- | --- | --- |
| control | UI | 266 | 18 | 5 | control VS cKO7 UI  p=0.8294  ns |
| cKO7 |  | 259 | 24 | 5 |  |
| control | 10dpi | 859 | 54 | 5 | control VS cKO7 10dpi  p=0.5996  ns |
| cKO7 |  | 898 | 46 | 5 |  |
| control | 20dpi | 701 | 34 | 5 | control VS cKO7 20dpi  p=0.9144  ns |
| cKO7 |  | 694 | 51 | 5 |  |

Unpaired t test

Figure 5 – figure supplement 1J – TEM Myelinating Schwann cell nuclei

| **Genotype** | **Time** | **Mean** | **SEM** | **n** | **Comparisons made**  **p-value** |
| --- | --- | --- | --- | --- | --- |
| control | UI | 193 | 9 | 5 | control VS cKO7 UI  p=>0.9999  ns |
| cKO7 |  | 193 | 20 | 5 |  |
| control | 10dpi | 181 | 21 | 5 | control VS cKO7 10dpi  p=0.3590  ns |
| cKO7 |  | 155 | 17 | 5 |  |
| control | 20dpi | 305 | 13 | 5 | control VS cKO7 20dpi  p=0.9885  ns |
| cKO7 |  | 305 | 23 | 5 |  |

Unpaired t test

Figure 5 – figure supplement 1K – % Axons >1.5µm

| **Genotype** | **Time** | **Mean** | **SEM** | **n** | **Comparisons made**  **p-value** |
| --- | --- | --- | --- | --- | --- |
| control | UI | 100.00 | 0 | 5 | control VS cKO7 UI  p=0.1886  ns |
| cKO7 |  | 99.84 | 0.11 | 5 |  |
| control | 10dpi | 54.85 | 1.11 | 5 | control VS cKO7 10dpi  p=0.1885  ns |
| cKO7 |  | 47.82 | 4.76 | 5 |  |
| control | 20dpi | 98.35 | 0.19 | 5 | control VS cKO7 20dpi  p=0.0561  ns |
| cKO7 |  | 97.74 | 0.19 | 5 |  |

Unpaired t test

Figure 5 – figure supplement 1L – Western Blot 10 y 21 dpi cKO7: JUN

| **Genotype** | **Time** | **Mean** | **SEM** | **n** | **Comparisons made**  **p-value** |
| --- | --- | --- | --- | --- | --- |
| UI | - | 0.29 | 0.03 | 6 | N / A |
| control | 10dpi | 1 | 0.04 | 7 | control VS cKO7 10dpi  p=0.3672  ns |
| cKO7 |  | 1.07 | 0.07 | 7 |  |
| control | 21dpi | 1.1 | 0.13 | 9 | control VS cKO7 20dpi  p=0.3015  ns |
| cKO7 |  | 1.34 | 0.18 | 9 |  |

Unpaired t test

Figure 5 – figure supplement 1L – Western Blot 10 y 21 dpi cKO7: KROX-20

| **Genotype** | **Time** | **Mean** | **SEM** | **n** | **Comparisons made**  **p-value** |
| --- | --- | --- | --- | --- | --- |
| UI | - | 0.77 | 0.08 | 6 | N / A |
| control | 10dpi | 1 | 0.02 | 7 | control VS cKO7 10dpi  p=0.4219  ns |
| cKO7 |  | 1.06 | 0.07 | 7 |  |
| control | 21dpi | 1.28 | 0.12 | 9 | control VS cKO7 20dpi  p=0.6097  ns |
| cKO7 |  | 1.17 | 0.15 | 9 |  |

Unpaired t test

Figure 5 – figure supplement 1L – Western Blot 10 y 21 dpi cKO7: MPZ

| **Genotype** | **Time** | **Mean** | **SEM** | **n** | **Comparisons made**  **p-value** |
| --- | --- | --- | --- | --- | --- |
| UI | - | 1 | 0.05 | 6 | N / A |
| control | 10dpi | 0.11 | 0.004 | 7 | control VS cKO7 10dpi  p=0.2836  ns |
| cKO7 |  | 0.11 | 0.006 | 7 |  |
| control | 21dpi | 0.37 | 0.02 | 9 | control VS cKO7 20dpi  p=0.1703  ns |
| cKO7 |  | 0.33 | 0.03 | 9 |  |

Unpaired t test

Figure 5 – figure supplement 1M – qPCR 10 dpi cKO7 mRNA fold change injured vs uninjured

| **Genotype** | **gen** | **Mean** | **SEM** | **n** | **Comparisons made**  **p-value** |
| --- | --- | --- | --- | --- | --- |
| control | *Jun* | 3.40 | 0.28 | 4 | control VS cKO7  p=0.3765  ns |
| cKO7 |  | 3.00 | 0.32 | 4 |  |

Unpaired t test

| **Genotype** | **gen** | **Mean** | **SEM** | **n** | **Comparisons made**  **p-value** |
| --- | --- | --- | --- | --- | --- |
| control | *Gdnf* | 28.48 | 4.82 | 4 | control VS cKO7  p=0.3608  ns |
| cKO7 |  | 37.41 | 7.63 | 4 |  |

Unpaired t test

Figure 5 – figure supplement 1N – qPCR 10 dpi cKO7 mRNA fold change injured vs uninjured

| **Genotype** | **gen** | **Mean** | **SEM** | **n** | **Comparisons made**  **p-value** |
| --- | --- | --- | --- | --- | --- |
| control | *Krox-20* | 0.52 | 0.13 | 4 | control VS cKO7  p=0.6124  ns |
| cKO7 |  | 0.44 | 0.06 | 4 |  |

Unpaired t test

| **Genotype** | **gen** | **Mean** | **SEM** | **n** | **Comparisons made**  **p-value** |
| --- | --- | --- | --- | --- | --- |
| control | *Prx* | 0.28 | 0.04 | 4 | control VS cKO7  p=0.3022  ns |
| cKO7 |  | 0.22 | 0.03 | 4 |  |

Unpaired t test

| **Genotype** | **gen** | **Mean** | **SEM** | **n** | **Comparisons made**  **p-value** |
| --- | --- | --- | --- | --- | --- |
| control | *Mpz* | 0.30 | 0.05 | 4 | control VS cKO7  p=0.4469  ns |
| cKO7 |  | 0.25 | 0.02 | 4 |  |

Unpaired t test

Figure 5 – figure supplement 1O – Number of intact myelin sheaths per nerve after 4 day cut cKO7

| **Genotype** | **Time** | **Mean** | **SEM** | **n** | **Comparisons made**  **p-value** |
| --- | --- | --- | --- | --- | --- |
| control | Cut 4d | 290 | 15 | 3 | control VS cKO7  p=0.0984  ns |
| cKO7 |  | 355 | 23 | 5 |  |

Unpaired t test

**Figure 5 – figure supplement 2**

Figure 5 – figure supplement 2A – qPCR Repair cells *Jun*

| **Genotype** | **Time** | **Mean** | **SEM** | **n** | **Comparisons made**  **p-value** |
| --- | --- | --- | --- | --- | --- |
| control | UI | 1 | 0.11 | 8 | control VS tKO UI  p<0.0001  *** |
| tKO |  | 2.19 | 0.19 | 10 |  |
| control | 1d | 4.44 | 0.15 | 3 | control VS tKO 1d  p=0.0009  *** |
| tKO |  | 8.16 | 0.39 | 3 |  |
| control | 4d | 2.93 | 0.20 | 3 | control VS tKO 4d  p=0.0438  * |
| tKO |  | 5.23 | 0.71 | 4 |  |
| control | 7d | 5.24 | 1.12 | 3 | control VS tKO 7d  p=0.6816  ns |
| tKO |  | 4.71 | 0.43 | 3 |  |

Unpaired t test

Figure 5 – figure supplement 2B – qPCR Repair cells *Olig1*

| **Genotype** | **Time** | **Mean** | **SEM** | **n** | **Comparisons made**  **p-value** |
| --- | --- | --- | --- | --- | --- |
| control | UI | 0.39 | 0.09 | 10 | control VS tKO UI  p<0.0001  *** |
| tKO |  | 8.48 | 1.49 | 11 |  |
| control | 1d | 0.43 | 0.09 | 3 | control VS tKO 1d  p=0.1295  ns |
| tKO |  | 2.23 | 0.94 | 3 |  |
| control | 4d | 9.47 | 2.24 | 5 | control VS tKO 4d  p=0.0142  * |
| tKO |  | 25.16 | 4.50 | 5 |  |
| control | 7d | 175.30 | 36.87 | 3 | control VS tKO 7d  p=0.8036  ns |
| tKO |  | 194.37 | 61.58 | 3 |  |

Unpaired t test

Figure 5 – figure supplement 2C – qPCR Repair cells *Shh*

| **Genotype** | **Time** | **Mean** | **SEM** | **n** | **Comparisons made**  **p-value** |
| --- | --- | --- | --- | --- | --- |
| control | UI | 1 | 0.12 | 11 | control VS tKO UI  p=0.1201  ns |
| tKO |  | 1.26 | 0.11 | 11 |  |
| control | 1d | 89.39 | 8.25 | 3 | control VS tKO 1d  p=0.0048  ** |
| tKO |  | 154.58 | 8.01 | 3 |  |
| control | 4d | 42.33 | 7.70 | 5 | control VS tKO 4d  p=0.8409  ns |
| tKO |  | 39.39 | 11.92 | 5 |  |
| control | 7d | 50.57 | 14.70 | 3 | control VS tKO 7d  p=0.7707  ns |
| tKO |  | 44.36 | 13.44 | 3 |  |

Unpaired t test

Figure 5 – figure supplement 2D – qPCR Repair cells *Ngfr*

| **Genotype** | **Time** | **Mean** | **SEM** | **n** | **Comparisons made**  **p-value** |
| --- | --- | --- | --- | --- | --- |
| control | UI | 0.55 | 0.05 | 9 | control VS tKO UI  p<0.0001  *** |
| tKO |  | 3.19 | 0.31 | 10 |  |
| control | 1d | 1.82 | 0.36 | 3 | control VS tKO 1d  p=0.0270  * |
| tKO |  | 3.74 | 0.43 | 3 |  |
| control | 4d | 5.82 | 0.42 | 4 | control VS tKO 4d  p=0.0382  * |
| tKO |  | 10.17 | 1.59 | 4 |  |
| control | 7d | 10.48 | 3.00 | 3 | control VS tKO 7d  p=0.5986  ns |
| tKO |  | 8.63 | 1.26 | 3 |  |

Unpaired t test

Figure 5 – figure supplement 2E – qPCR Repair cells *Mpz*

| **Genotype** | **Time** | **Mean** | **SEM** | **n** | **Comparisons made**  **p-value** |
| --- | --- | --- | --- | --- | --- |
| control | UI | 1 | 0.07 | 8 | control VS tKO UI  p=0.0398  * |
| tKO |  | 0.76 | 0.08 | 8 |  |
| control | 1d | 0.59 | 0.16 | 3 | control VS tKO 1d  p=0.6012  ns |
| tKO |  | 0.5 | 0.07 | 3 |  |
| control | 4d | 0.0012 | 0.00009 | 5 | control VS tKO 4d  p=0.0037  ** |
| tKO |  | 0.0005 | 0.00013 | 5 |  |

Unpaired t test

Figure 5 – figure supplement 2F – qPCR Repair cells *Gdnf*

| **Genotype** | **Time** | **Mean** | **SEM** | **n** | **Comparisons made**  **p-value** |
| --- | --- | --- | --- | --- | --- |
| control | UI | 1 | 0.12 | 10 | control VS tKO UI  p<0.0001  *** |
| tKO |  | 5.30 | 0.67 | 11 |  |
| control | 1d | 245.57 | 49.90 | 3 | control VS tKO 1d  p=0.1898  ns |
| tKO |  | 340.50 | 33.61 | 3 |  |
| control | 4d | 89.87 | 10.84 | 5 | control VS tKO 4d  p=0.0876  ns |
| tKO |  | 137.87 | 23.72 | 4 |  |
| control | 7d | 112.99 | 48.56 | 3 | control VS tKO 7d  p=0.6007  ns |
| tKO |  | 148.82 | 40.35 | 3 |  |

Unpaired t test

Figure 5 – figure supplement 2G – qPCR Repair cells *Ulk1 y Vps34*

| **Genotype** | **Gene** | **Time** | **Mean** | **SEM** | **n** | **Comparisons made**  **p-value** |
| --- | --- | --- | --- | --- | --- | --- |
| control | *Ulk1* | UI | 6.10 | 0.26 | 5 | control VS tKO UI  p=1275  ns |
| tKO |  |  | 7.64 | 0.20 | 5 |  |
| control |  | 4d cut | 5.11 | 0.52 | 5 | control VS tKO UI  p=6158  ns |
| tKO |  |  | 5.35 | 0.85 | 5 |  |

Unpaired t test

| **Genotype** | **Gene** | **Time** | **Mean** | **SEM** | **n** | **Comparisons made**  **p-value** |
| --- | --- | --- | --- | --- | --- | --- |
| control | *Vps34* | UI | 7.65 | 0.70 | 5 | control VS tKO UI  p=2227  ns |
| tKO |  |  | 9.01 | 0.21 | 5 |  |
| control |  | 4d cut | 4.42 | 0.79 | 5 | control VS tKO UI  p=5227  ns |
| tKO |  |  | 4.71 | 0.54 | 5 |  |

Unpaired t test

Figure 5 – figure supplement 2H – WB: LC3B

| **Genotype** | **Time** | **Mean** | **SEM** | **n** | **Comparisons made**  **p-value** |
| --- | --- | --- | --- | --- | --- |
| WT | UI | 1.00 | 0 | 3 | WT vs control p=0.7565 ns  WT vs tKO p=0.8134 ns  control vs tKO p=0.4635 ns |
| control |  | 1.05 | 0.04 | 3 |  |
| tKO |  | 0.95 | 0.02 | 3 |  |
| WT | 2d cut | 1.45 | 0.07 | 2 | WT vs control p=0.9248 ns  WT vs tKO p=0.8952 ns  control vs tKO p=0.9969 ns |
| control |  | 1.63 | 0.09 | 2 |  |
| tKO |  | 1.66 | 0.37 | 2 |  |
| WT | 4d cut | 1.83 | 0.33 | 2 | WT vs control p=0.9920 ns  WT vs tKO p=0.3762 ns  control vs tKO p=0.3372 ns |
| control |  | 1.89 | 0.30 | 2 |  |
| tKO |  | 1.00 | 0.08 | 2 |  |

One-way Anova Tukey test UI p=0.4864

One-way Anova Tukey test 2d cut p=0.8911

One-way Anova Tukey test 4d cut p=0.3048

Figure 5 – figure supplement 2I – Macrophages numbers after 4d cut (F4/80+ cells)

| **Genotype** | **Time** | **Mean** | **SEM** | **n** | **Comparisons made**  **p-value** |
| --- | --- | --- | --- | --- | --- |
| control | 4d cut | 41.83 | 0.91 | X | control VS cKO7 UI  p=0.3857  ns |
| tKO |  | 36.00 | 1.43 | X |  |

Unpaired t test

**Figure 5 – figure supplement 3**

Figure 5 – figure supplement 3A – Immunofluorescence SOX10 and JUN

| **Genotype** | **Time** | **Mean** | **SEM** | **n** | **Comparisons made**  **p-value** |
| --- | --- | --- | --- | --- | --- |
| control | UI | 0.78 | 0.33 | 4 | control VS tKO UI  p=0.0023  ** |
| tKO |  | 4.47 | 0.65 | 4 |  |
| control | 10d | 10.15 | 1.33 | 4 | control VS tKO 1d  p=0.0001  *** |
| tKO |  | 24.58 | 0.92 | 4 |  |

Unpaired t test

Figure 5 – figure supplement 3B – Immunofluorescence SOX10 and Ki67

| **Genotype** | **Time** | **Mean** | **SEM** | **n** | **Comparisons made**  **p-value** |
| --- | --- | --- | --- | --- | --- |
| control | UI | 0 | 0 | 3 | control VS tKO UI  p=0.3739  ns |
| tKO |  | 0.22 | 0.22 | 3 |  |
| control | 10d | 1.05 | 0.46 | 3 | control VS tKO 1d  p=0.0142  * |
| tKO |  | 5.06 | 0.85 | 3 |  |

Unpaired t test

**Figure 5 – figure supplement 5**

Figure 5 – figure supplement 5B – RNA-seq *Jun*

| **Genotype** | **Dpi** | **Mean** | **SEM** | **n** | **Comparisons made**  **p-value** |
| --- | --- | --- | --- | --- | --- |
| control | UI | 1 | 0.05 | 3 | control VS tKO UI  p=0.0153  * |
| tKO |  | 2.14 | 0.02 | 3 |  |
| control | 1 | 2.86 | 0.22 | 3 | control VS tKO 1dpi  p<0.0001  **** |
| tKO |  | 5.84 | 0.11 | 3 |  |
| control | 10 | 3.54 | 0.08 | 3 | control VS tKO 10dpi  p<0.0001  **** |
| tKO |  | 6.4 | 0.31 | 3 |  |
| control | 20 | 3.13 | 0.28 | 3 | control VS tKO 20dpi  p<0.0001  **** |
| tKO |  | 6.64 | 0.46 | 3 |  |

2 Way ANOVA with Sidak's multiple comparisons test

Figure 5 – figure supplement 5C – RNA-seq *Runx2*

| **Genotype** | **Dpi** | **Mean** | **SEM** | **n** | **Comparisons made**  **p-value** |
| --- | --- | --- | --- | --- | --- |
| control | UI | 1 | 0.03 | 3 | control VS tKO UI  p=0.9539  ns |
| tKO |  | 4.13 | 0.53 | 3 |  |
| control | 1 | 17.69 | 1.35 | 3 | control VS tKO 1dpi  p<0.0001  **** |
| tKO |  | 47.95 | 1.51 | 3 |  |
| control | 10 | 26.61 | 4.66 | 3 | control VS tKO 10dpi  p=0.0002  *** |
| tKO |  | 53.56 | 7.34 | 3 |  |
| control | 20 | 21.75 | 2.99 | 3 | control VS tKO 20dpi  p=0.0041  ** |
| tKO |  | 41.59 | 3.06 | 3 |  |

2 Way ANOVA with Sidak's multiple comparisons test

Figure 5 – figure supplement 5D – RNA-seq *Gdnf*

| **Genotype** | **Dpi** | **Mean** | **SEM** | **n** | **Comparisons made**  **p-value** |
| --- | --- | --- | --- | --- | --- |
| control | UI | 1 | 0.47 | 3 | control VS tKO UI  p=0.9993  ns |
| tKO |  | 6.08 | 1.24 | 3 |  |
| control | 1 | 234.43 | 41.92 | 3 | control VS tKO 1dpi  p=0.0002  *** |
| tKO |  | 369.69 | 13.60 | 3 |  |
| control | 10 | 29.69 | 5.12 | 3 | control VS tKO 10dpi  p=0.0085  ** |
| tKO |  | 119.02 | 15.15 | 3 |  |
| control | 20 | 19.31 | 3.31 | 3 | control VS tKO 20dpi  p=0.1942  ns |
| tKO |  | 70.46 | 13.24 | 3 |  |

2 Way ANOVA with Sidak's multiple comparisons test

Figure 5 – figure supplement 5E – RNA-seq *Ngfr*

| **Genotype** | **Dpi** | **Mean** | **SEM** | **n** | **Comparisons made**  **p-value** |
| --- | --- | --- | --- | --- | --- |
| control | UI | 1 | 0.06 | 3 | control VS tKO UI  p=0.0972  ns |
| tKO |  | 5.61 | 0.30 | 3 |  |
| control | 1 | 2.20 | 0.26 | 3 | control VS tKO 1dpi  p=0.1491  ns |
| tKO |  | 6.39 | 0.41 | 3 |  |
| control | 10 | 11.87 | 2.03 | 3 | control VS tKO 10dpi  p=0.0181  * |
| tKO |  | 18.03 | 1.84 | 3 |  |
| control | 20 | 9.83 | 1.30 | 3 | control VS tKO 20dpi  p=0.0164  * |
| tKO |  | 16.08 | 2.10 | 3 |  |

2 Way ANOVA with Sidak's multiple comparisons test

Figure 5 – figure supplement 5F – RNA-seq *Sox2*

| **Genotype** | **Dpi** | **Mean** | **SEM** | **n** | **Comparisons made**  **p-value** |
| --- | --- | --- | --- | --- | --- |
| control | UI | 1 | 0.05 | 3 | control VS tKO UI  p=0.7312  ns |
| tKO |  | 1.57 | 0.03 | 3 |  |
| control | 1 | 0.99 | 0.20 | 3 | control VS tKO 1dpi  p=0.0009  *** |
| tKO |  | 3.4 | 0.32 | 3 |  |
| control | 10 | 5.36 | 0.86 | 3 | control VS tKO 10dpi  p=0.8992  ns |
| tKO |  | 4.95 | 0.27 | 3 |  |
| control | 20 | 3.85 | 0.22 | 3 | control VS tKO 20dpi  p=0.1906  ns |
| tKO |  | 4.93 | 0.22 | 3 |  |

2 Way ANOVA with Sidak's multiple comparisons test

Figure 5 – figure supplement 5G – RNA-seq *Pou3f1*

| **Genotype** | **Dpi** | **Mean** | **SEM** | **n** | **Comparisons made**  **p-value** |
| --- | --- | --- | --- | --- | --- |
| control | UI | 1 | 0.08 | 3 | control VS tKO UI  p=0.1448  ns |
| tKO |  | 5.46 | 0.28 | 3 |  |
| control | 1 | 1.90 | 0.28 | 3 | control VS tKO 1dpi  p=0.8347  ns |
| tKO |  | 3.75 | 0.67 | 3 |  |
| control | 10 | 14.15 | 1.14 | 3 | control VS tKO 10dpi  p=0.7217  Nns |
| tKO |  | 16.39 | 2.93 | 3 |  |
| control | 20 | 4.74 | 0.56 | 3 | control VS tKO 20dpi  p<0.0001  **** |
| tKO |  | 19.87 | 2.19 | 3 |  |

2 Way ANOVA with Sidak's multiple comparisons test

Figure 5 – figure supplement 5H – RNA-seq *Krox-20*

| **Genotype** | **Dpi** | **Mean** | **SEM** | **n** | **Comparisons made**  **p-value** |
| --- | --- | --- | --- | --- | --- |
| control | UI | 1 | 0.08 | 3 | control VS tKO UI  p=0.6311  ns |
| tKO |  | 0.82 | 0.06 | 3 |  |
| control | 1 | 1.33 | 0.10 | 3 | control VS tKO 1dpi  p=0.1473  ns |
| tKO |  | 1.02 | 0.11 | 3 |  |
| control | 10 | 0.76 | 0.14 | 3 | control VS tKO 10dpi  p=0.0283  * |
| tKO |  | 0.33 | 0.04 | 3 |  |
| control | 20 | 0.65 | 0.13 | 3 | control VS tKO 20dpi  p=0.0457  * |
| tKO |  | 0.25 | 0.08 | 3 |  |

2 Way ANOVA with Sidak's multiple comparisons test

Figure 5 – figure supplement 5I – RNA-seq *Drp2*

| **Genotype** | **Dpi** | **Mean** | **SEM** | **n** | **Comparisons made**  **p-value** |
| --- | --- | --- | --- | --- | --- |
| control | UI | 1 | 0.05 | 3 | control VS tKO UI  p=0.0005  *** |
| tKO |  | 0.73 | 0.07 | 3 |  |
| control | 1 | 0.95 | 0.05 | 3 | control VS tKO 1dpi  p<0.0001  **** |
| tKO |  | 0.60 | 0.03 | 3 |  |
| control | 10 | 0.05 | 0.01 | 3 | control VS tKO 10dpi  p=0.8647  ns |
| tKO |  | 0.01 | 0.002 | 3 |  |
| control | 20 | 0.28 | 0.01 | 3 | control VS tKO 20dpi  p=0.0015  ** |
| tKO |  | 0.04 | 0.004 | 3 |  |

2 Way ANOVA with Sidak's multiple comparisons test

Figure 5 – figure supplement 5J – RNA-seq *Prx*

| **Genotype** | **Dpi** | **Mean** | **SEM** | **n** | **Comparisons made**  **p-value** |
| --- | --- | --- | --- | --- | --- |
| control | UI | 1 | 0.05 | 3 | control VS tKO UI  p=0.0076  ** |
| tKO |  | 0.81 | 0.05 | 3 |  |
| control | 1 | 0.48 | 0.04 | 3 | control VS tKO 1dpi  p=0.0344  * |
| tKO |  | 0.33 | 0.02 | 3 |  |
| control | 10 | 0.40 | 0.02 | 3 | control VS tKO 10dpi  p<0.0001  **** |
| tKO |  | 0.03 | 0.003 | 3 |  |
| control | 20 | 0.65 | 0.05 | 3 | control VS tKO 20dpi  p<0.0001  **** |
| tKO |  | 0.16 | 0.02 | 3 |  |

2 Way ANOVA with Sidak's multiple comparisons test

Figure 5 – figure supplement 5K – RNA-seq *Mpz*

| **Genotype** | **Dpi** | **Mean** | **SEM** | **n** | **Comparisons made**  **p-value** |
| --- | --- | --- | --- | --- | --- |
| control | UI | 1 | 0.03 | 3 | control VS tKO UI  p=0.0977  ns |
| tKO |  | 0.85 | 0.04 | 3 |  |
| control | 1 | 0.47 | 0.06 | 3 | control VS tKO 1dpi  P=0.2869  ns |
| tKO |  | 0.36 | 0.02 | 3 |  |
| control | 10 | 0.51 | 0.05 | 3 | control VS tKO 10dpi  p<0.0001  **** |
| tKO |  | 0.05 | 0.01 | 3 |  |
| control | 20 | 0.74 | 0.07 | 3 | control VS tKO 20dpi  p<0.0001  **** |
| tKO |  | 0.23 | 0.02 | 3 |  |

2 Way ANOVA with Sidak's multiple comparisons test

Figure 5 – figure supplement 5L – RNA-seq *Mbp*

| **Genotype** | **Dpi** | **Mean** | **SEM** | **n** | **Comparisons made**  **p-value** |
| --- | --- | --- | --- | --- | --- |
| control | UI | 1 | 0.07 | 3 | control VS tKO UI  p=0.2119  ns |
| tKO |  | 0.87 | 0.05 | 3 |  |
| control | 1 | 0.53 | 0.08 | 3 | control VS tKO 1dpi  p=0.3651  ns |
| tKO |  | 0.43 | 0.01 | 3 |  |
| control | 10 | 0.29 | 0.03 | 3 | control VS tKO 10dpi  p=0.0031  ** |
| tKO |  | 0.04 | 0.003 | 3 |  |
| control | 20 | 0.56 | 0.03 | 3 | control VS tKO 20dpi  p<0.0001  **** |
| tKO |  | 0.17 | 0.01 | 3 |  |

2 Way ANOVA with Sidak's multiple comparisons test

Figure 5 – figure supplement 5M – RNA-seq *Mag*

| **Genotype** | **Dpi** | **Mean** | **SEM** | **n** | **Comparisons made**  **p-value** |
| --- | --- | --- | --- | --- | --- |
| control | UI | 1 | 0.02 | 3 | control VS tKO UI  p=0.6798  ns |
| tKO |  | 0.9 | 0.06 | 3 |  |
| control | 1 | 0.68 | 0.10 | 3 | control VS tKO 1dpi  p=0.8730  ns |
| tKO |  | 0.61 | 0.004 | 3 |  |
| control | 10 | 1.37 | 0.10 | 3 | control VS tKO 10dpi  p<0.0001  **** |
| tKO |  | 0.10 | 0.01 | 3 |  |
| control | 20 | 1.03 | 0.07 | 3 | control VS tKO 20dpi  p<0.0001  **** |
| tKO |  | 0.48 | 0.03 | 3 |  |

2 Way ANOVA with Sidak's multiple comparisons test

Figure 5 – figure supplement 5N – RNA-seq *Pmp22*

| **Genotype** | **Dpi** | **Mean** | **SEM** | **n** | **Comparisons made**  **p-value** |
| --- | --- | --- | --- | --- | --- |
| control | UI | 1 | 0.04 | 3 | control VS tKO UI  p=0.0356  * |
| tKO |  | 0.85 | 0.05 | 3 |  |
| control | 1 | 0.64 | 0.07 | 3 | control VS tKO 1dpi  p=0.1038  ns |
| tKO |  | 0.51 | 0.01 | 3 |  |
| control | 10 | 0.27 | 0.02 | 3 | control VS tKO 10dpi  p=0.0011  ** |
| tKO |  | 0.03 | 0.003 | 3 |  |
| control | 20 | 0.66 | 0.01 | 3 | control VS tKO 20dpi  p<0.0001  **** |
| tKO |  | 0.16 | 0.01 | 3 |  |

2 Way ANOVA with Sidak's multiple comparisons test

Figure 5 – figure supplement 5O – RNA-seq *Plp1*

| **Genotype** | **Dpi** | **Mean** | **SEM** | **n** | **Comparisons made**  **p-value** |
| --- | --- | --- | --- | --- | --- |
| control | UI | 1 | 0.05 | 3 | control VS tKO UI  p=0.3908  ns |
| tKO |  | 1.19 | 0.07 | 3 |  |
| control | 1 | 0.70 | 0.06 | 3 | control VS tKO 1dpi  p=0.5810  ns |
| tKO |  | 0.85 | 0.04 | 3 |  |
| control | 10 | 1.35 | 0.17 | 3 | control VS tKO 10dpi  p=0.0004  *** |
| tKO |  | 0.77 | 0.08 | 3 |  |
| control | 20 | 1.23 | 0.05 | 3 | control VS tKO 20dpi  p=0.9576  ns |
| tKO |  | 1.16 | 0.02 | 3 |  |

2 Way ANOVA with Sidak's multiple comparisons test

Figure 5 – figure supplement 5P – RNA-seq *Hmgcr1*

| **Genotype** | **Dpi** | **Mean** | **SEM** | **n** | **Comparisons made**  **p-value** |
| --- | --- | --- | --- | --- | --- |
| control | UI | 1 | 0.01 | 3 | control VS tKO UI  p=0.3016  ns |
| tKO |  | 0.78 | 0.03 | 3 |  |
| control | 1 | 0.38 | 0.08 | 3 | control VS tKO 1dpi  p=0.9161  ns |
| tKO |  | 0.32 | 0.05 | 3 |  |
| control | 10 | 0.20 | 0.03 | 3 | control VS tKO 10dpi  p=0.0757  ns |
| tKO |  | 0.05 | 0.01 | 3 |  |
| control | 20 | 0.42 | 0.004 | 3 | control VS tKO 20dpi  p=0.2739  ns |
| tKO |  | 0.15 | 0.01 | 3 |  |

2 Way ANOVA with Sidak's multiple comparisons test

Figure 5 – figure supplement 5Q – RNA-seq *Lss*

| **Genotype** | **Dpi** | **Mean** | **SEM** | **n** | **Comparisons made**  **p-value** |
| --- | --- | --- | --- | --- | --- |
| control | UI | 1 | 0.002 | 3 | control VS tKO UI  p=0.0002  *** |
| tKO |  | 0.74 | 0.002 | 3 |  |
| control | 1 | 0.45 | 0.06 | 3 | control VS tKO 1dpi  p=0.3555  ns |
| tKO |  | 0.37 | 0.04 | 3 |  |
| control | 10 | 0.27 | 0.05 | 3 | control VS tKO 10dpi  p=0.0047  ** |
| tKO |  | 0.09 | 0.01 | 3 |  |
| control | 20 | 0.34 | 0.01 | 3 | control VS tKO 20dpi  p=0.0247  * |
| tKO |  | 0.20 | 0.02 | 3 |  |

2 Way ANOVA with Sidak's multiple comparisons test

Figure 5 – figure supplement 5R – RNA-seq *Dhcr24*

| **Genotype** | **Dpi** | **Mean** | **SEM** | **n** | **Comparisons made** | **p-value** |
| --- | --- | --- | --- | --- | --- | --- |
| control | UI | 1 | 0.02 | 3 | control VS tKO UI  p=0.0071  ** | |
| tKO |  | 0.81 | 0.06 | 3 |  |  |
| control | 1 | 0.56 | 0.06 | 3 | control VS tKO 1dpi  p=0.0501  ns | |
| tKO |  | 0.42 | 0.03 | 3 |  |  |
| control | 10 | 0.17 | 0.02 | 3 | control VS tKO 10dpi  p=0.0423  * | |
| tKO |  | 0.03 | 0.002 | 3 |  |  |
| control | 20 | 0.30 | 0.02 | 3 | control VS tKO 20dpi  p=0.0091  ** | |
| tKO |  | 0.12 | 0.02 | 3 |  |  |

2 Way ANOVA with Sidak's multiple comparisons test

Figure 5 – figure supplement 5S – RNA-seq *Elovl1*

| **Genotype** | **Dpi** | **Mean** | **SEM** | **n** | **Comparisons made**  **p-value** |
| --- | --- | --- | --- | --- | --- |
| control | UI | 1 | 0.01 | 3 | control VS tKO UI  p=0.0124  * |
| tKO |  | 0.86 | 0.01 | 3 |  |
| control | 1 | 0.74 | 0.06 | 3 | control VS tKO 1dpi  p=0.0076  ** |
| tKO |  | 0.59 | 0.01 | 3 |  |
| control | 10 | 0.61 | 0.03 | 3 | control VS tKO 10dpi  pP<0.0001  **** |
| tKO |  | 0.22 | 0.01 | 3 |  |
| control | 20 | 0.67 | 0.03 | 3 | control VS tKO 20dpi  p<0.0001  **** |
| tKO |  | 0.36 | 0.02 | 3 |  |

2 Way ANOVA with Sidak's multiple comparisons test

Figure 5 – figure supplement 5T – RNA-seq *Pmp2*

| **Genotype** | **Dpi** | **Mean** | **SEM** | **n** | **Comparisons made**  **p-value** |
| --- | --- | --- | --- | --- | --- |
| control | UI | 1 | 0.02 | 3 | control VS tKO UI  p=0.9992  ns |
| tKO |  | 0.98 | 0.01 | 3 |  |
| control | 1 | 0.53 | 0.01 | 3 | control VS tKO 1dpi  p=0.0150  * |
| tKO |  | 0.77 | 0.01 | 3 |  |
| control | 10 | 0.38 | 0.01 | 3 | control VS tKO 10dpi  p=0.0011  ** |
| tKO |  | 0.05 | 0.01 | 3 |  |
| control | 20 | 0.76 | 0.01 | 3 | control VS tKO 20dpi  p<0.0001  *** |
| tKO |  | 0.33 | 0.01 | 3 |  |

2 Way ANOVA with Sidak's multiple comparisons test

Figure 5 – figure supplement 5U – RNA-seq *Scd2*

| **Genotype** | **Dpi** | **Mean** | **SEM** | **n** | **Comparisons made**  **p-value** |
| --- | --- | --- | --- | --- | --- |
| control | UI | 1 | 0.02 | 3 | control VS tKO UI  p=0.0027  ** |
| tKO |  | 0.85 | 0.01 | 3 |  |
| control | 1 | 0.55 | 0.04 | 3 | control VS tKO 1dpi  p=0.0736  ns |
| tKO |  | 0.46 | 0.02 | 3 |  |
| control | 10 | 0.71 | 0.03 | 3 | control VS tKO 10dpi  p<0.0001  **** |
| tKO |  | 0.33 | 0.03 | 3 |  |
| control | 20 | 0.85 | 0.01 | 3 | control VS tKO 20dpi  p<0.0001  **** |
| tKO |  | 0.43 | 0.02 | 3 |  |

2 Way ANOVA with Sidak's multiple comparisons test

Figure 5 – figure supplement 5V – RNA-seq *Fads1*

| **Genotype** | **Dpi** | **Mean** | **SEM** | **n** | **Comparisons made**  **p-value** |
| --- | --- | --- | --- | --- | --- |
| control | UI | 1 | 0.04 | 3 | control VS tKO UI  p<0.0001  **** |
| tKO |  | 0.69 | 0.01 | 3 |  |
| control | 1 | 0.71 | 0.06 | 3 | control VS tKO 1dpi  p=0.0008  *** |
| tKO |  | 0.50 | 0.02 | 3 |  |
| control | 10 | 0.35 | 0.02 | 3 | control VS tKO 10dpi  p=0.2915  ns |
| tKO |  | 0.27 | 0.02 | 3 |  |
| control | 20 | 0.49 | 0.03 | 3 | control VS tKO 20dpi  p=0.0003  *** |
| tKO |  | 0.27 | 0.02 | 3 |  |

2 Way ANOVA with Sidak's multiple comparisons test

Figure 5 – figure supplement 5W – RNA-seq *Cers2*

| **Genotype** | **Dpi** | **Mean** | **SEM** | **n** | **Comparisons made**  **p-value** |
| --- | --- | --- | --- | --- | --- |
| control | UI | 1 | 0.01 | 3 | control VS tKO UI  p=0.0047  ** |
| tKO |  | 0.85 | 0.01 | 3 |  |
| control | 1 | 0.74 | 0.04 | 3 | control VS tKO 1dpi  p=0.1248  ns |
| tKO |  | 0.65 | 0.02 | 3 |  |
| control | 10 | 0.43 | 0.06 | 3 | control VS tKO 10dpi  p=0.0313  * |
| tKO |  | 0.32 | 0.01 | 3 |  |
| control | 20 | 0.58 | 0.02 | 3 | control VS tKO 20dpi  p<0.0001  **** |
| tKO |  | 0.35 | 0.02 | 3 |  |

2 Way ANOVA with Sidak's multiple comparisons test

Figure 5 – figure supplement 5X – RNA-seq *Ugt8a*

| **Genotype** | **Dpi** | **Mean** | **SEM** | **n** | **Comparisons made**  **p-value** |
| --- | --- | --- | --- | --- | --- |
| control | UI | 1 | 0.02 | 3 | control VS tKO UI  p=0.0077  ** |
| tKO |  | 0.78 | 0.03 | 3 |  |
| control | 1 | 0.50 | 0.10 | 3 | control VS tKO 1dpi  p=0.1013  ns |
| tKO |  | 0.35 | 0.04 | 3 |  |
| control | 10 | 0.33 | 0.04 | 3 | control VS tKO 10dpi  p<0.0005  *** |
| tKO |  | 0.02 | 0.001 | 3 |  |
| control | 20 | 0.59 | 0.02 | 3 | control VS tKO 20dpi  p<0.0001  **** |
| tKO |  | 0.14 | 0.02 | 3 |  |

2 Way ANOVA with Sidak's multiple comparisons test

**Figure 6 – figure supplement 1**

Figure 6 – figure supplement 1B – Voltage (V) A fibers

| **Genotype** | **Stimulus Intensity (V)** | **Mean** | **SEM** | **n** | **Comparisons made**  **p-value** |
| --- | --- | --- | --- | --- | --- |
| WT | 5 | 0.39 | 0.22 | 10 | Kruskal-Wallis  p<0.13  ns  No post-hoc |
| control |  | 0.31 | 0.19 | 16 |  |
| dKO |  | 1.57 | 0.56 | 7 |  |
| tKO |  | 0.22 | 0.14 | 7 |  |
| WT | 8 | 4.9 | 1.03 | 10 | Kruskal-Wallis  p=0.031  Post-Hoc:  WT vs Control p=0.004  Rest ns |
| control |  | 1.6 | 0.69 | 16 |  |
| dKO |  | 2.6 | 0.79 | 7 |  |
| tKO |  | 1.96 | 0.63 | 7 |  |
| WT | 10 | 5.3 | 1.22 | 10 | Kruskal-Wallis  p=0.015  Post-Hoc:  WT vs Control p=0.001  WT vs dKO= 0.048  WT vs tKO= 0.049 |
| control |  | 1.7 | 0.77 | 16 |  |
| dKO |  | 2 | 0.90 | 7 |  |
| tKO |  | 1.8 | 0.55 | 7 |  |
| WT | 15 | 6.5 | 1.3 | 10 | Kruskal-Wallis  p=0.017  Post-Hoc:  WT vs Control p=0.009  WT vs dKO= 0.006  WT vs tKO= 0.017 |
| control |  | 2.19 | 0.84 | 16 |  |
| dKO |  | 1.8 | 1.1 | 7 |  |
| tKO |  | 1.9 | 1.0 | 7 |  |

Statistic Test-> Kruskal Wallis with Mann-Whitney’s U post-hoc comparisons

Figure 6 – figure supplement 1C – Nerve Conduction Velocity (m/s)

| **Genotype** | **Stimulus Intensity (V)** | **Mean** | **SEM** | **N** | **Comparisons made**  **p-value** |
| --- | --- | --- | --- | --- | --- |
| WT | 5 | 20.9 | 8.1 | 10 | Kruskal-Wallis  p<0.13  ns  No post-hoc |
| control |  | 17.7 | 7.4 | 16 |  |
| dKO |  | 7.9 | 3.2 | 7 |  |
| tKO |  | 7.1 | 5.7 | 7 |  |
| WT | 8 | 49.6 | 11.9 | 10 | Kruskal-Wallis  p=0.025  Post-Hoc:  WT vs Control p=0.040  WT vs dKO = 0.096  WT vs tKO = 0.046 |
| control |  | 20.2 | 7.3 | 16 |  |
| dKO |  | 20.2 | 6.1 | 7 |  |
| tKO |  | 19.1 | 7.0 | 7 |  |
| WT | 10 | 46.2 | 9.7 | 10 | Kruskal-Wallis  p=0.047  Post-Hoc:  WT vs Control p=0.003  WT vs dKO = 0.058  WT vs tKO = 0.033 |
| control |  | 17.2 | 7.5 | 16 |  |
| dKO |  | 22.9 | 11.6 | 7 |  |
| tKO |  | 17.3 | 6.2 | 7 |  |
| WT | 15 | 51.9 | 11.9 | 10 | Kruskal-Wallis  p=0.016  Post-Hoc:  WT vs Control p=0.011  WT vs dKO = 0.010  WT vs tKO = 0.007 |
| control |  | 16.1 | 7.2 | 16 |  |
| dKO |  | 18.2 | 12.3 | 7 |  |
| tKO |  | 12.9 | 7.2 | 7 |  |

Statistic Test-> Kruskal Wallis with Mann-Whitney’s U post-hoc comparisons

**Figure 9 – figure supplement 1**

Figure 9 – figure supplement 1A – *Hdac9* mRNA level relative to 18S

| **Genotype** | **Gen** | **Mean** | **SEM** | **n** | **Comparisons made**  **p-value** |
| --- | --- | --- | --- | --- | --- |
| WT | *Hdac5* | 0.82x10^-6^ | 0.10x10^-6^ | 5 | WT VS Jun_OE  p=0.2613  ns |
| Jun_OE |  | 1.13x10^-6^ | 0.21x10^-6^ | 5 |  |

Unpaired t test
