# Supplementary material for: A genetic compensatory mechanism regulated by Jun and Mef2d modulates the expression of distinct class IIa Hdacs to ensure peripheral nerve myelination and repair
Source: eLife. 2022 Jan 25;11:e72917. doi: 10.7554/eLife.72917 (PMC8853665; doi:10.7554/eLife.72917)

Figure 1

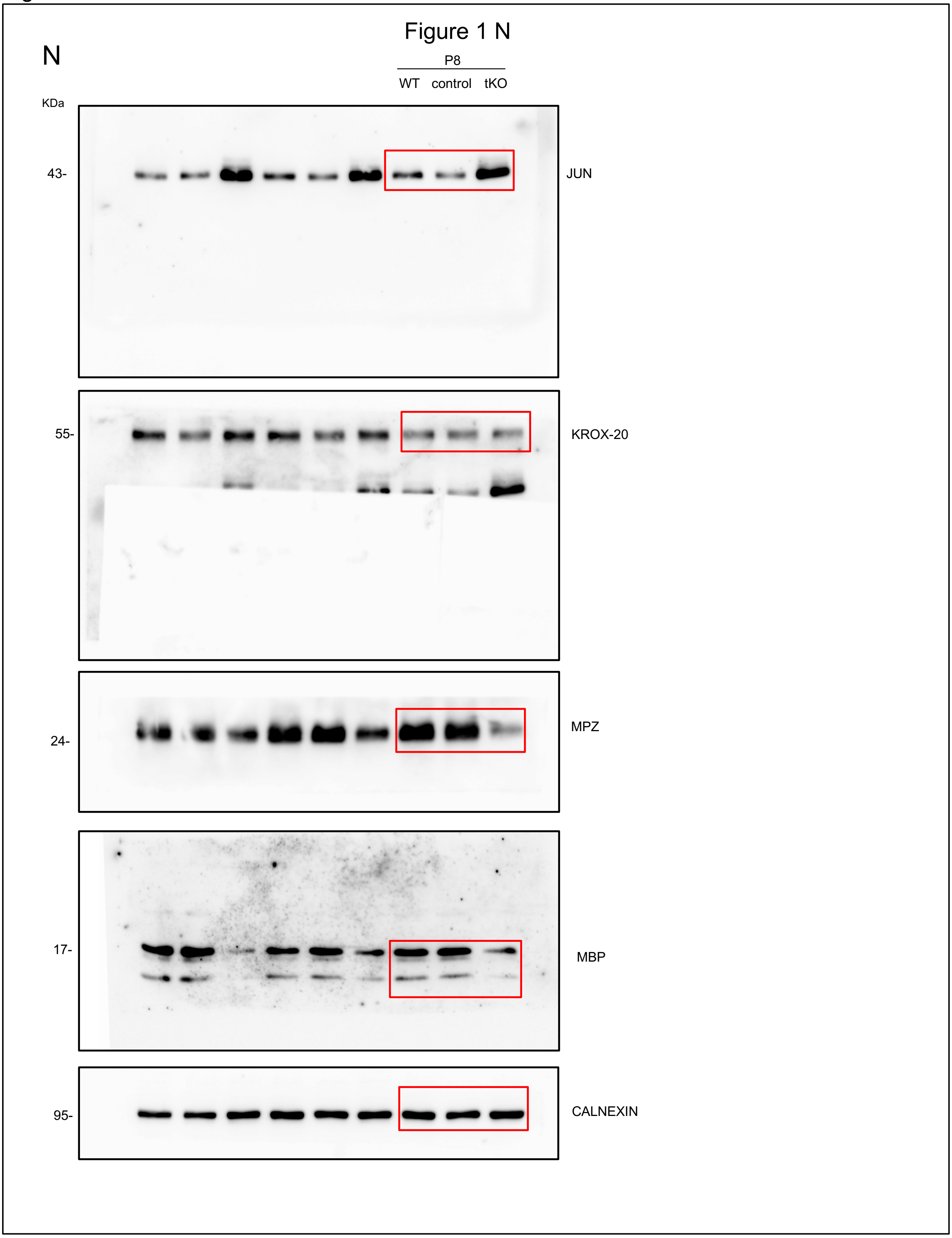

N
Figure 1 N
P8
WT control tKO
KDa
43-
JUN
55-
KROX-20
24-
MPZ
17-
MBP
95-
CALNEXIN

Figure 2

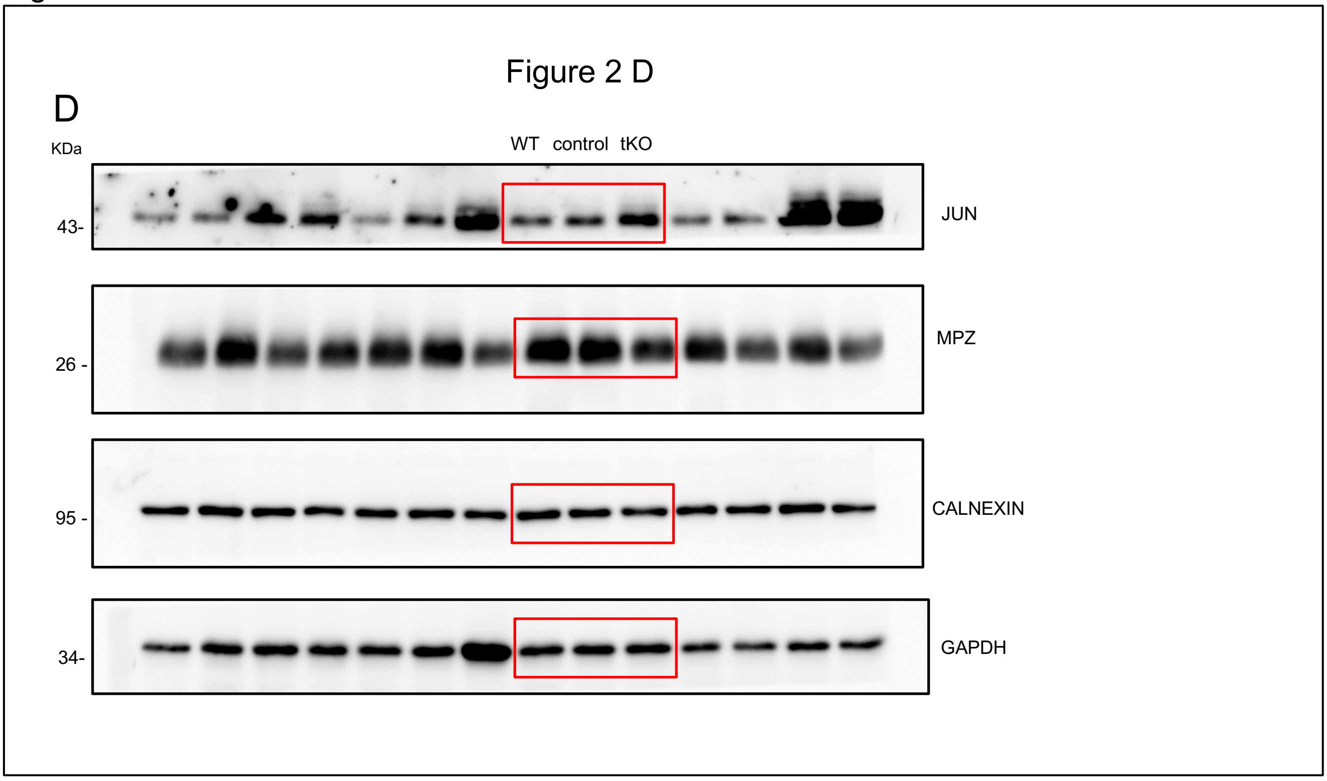
Figure 2 D
D
KDa
WT control tKO
43-
JUN
26 -
MPZ
95 -
CALNEXIN
34-
GAPDH

Figure 3

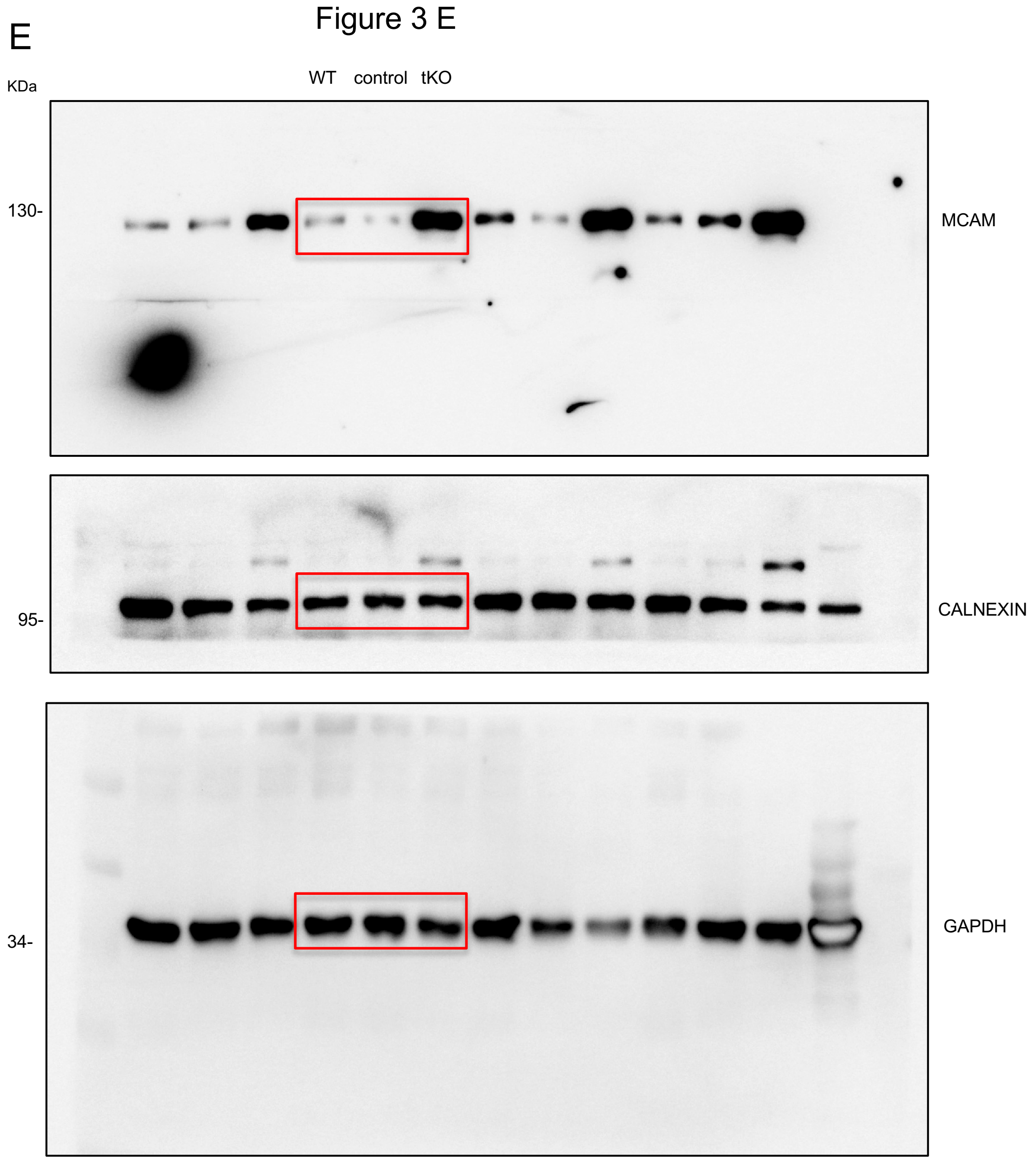
E
Figure 3 E
KDa
WT control tKO
130-
MCAM
95-
CALNEXIN
34-
GAPDH

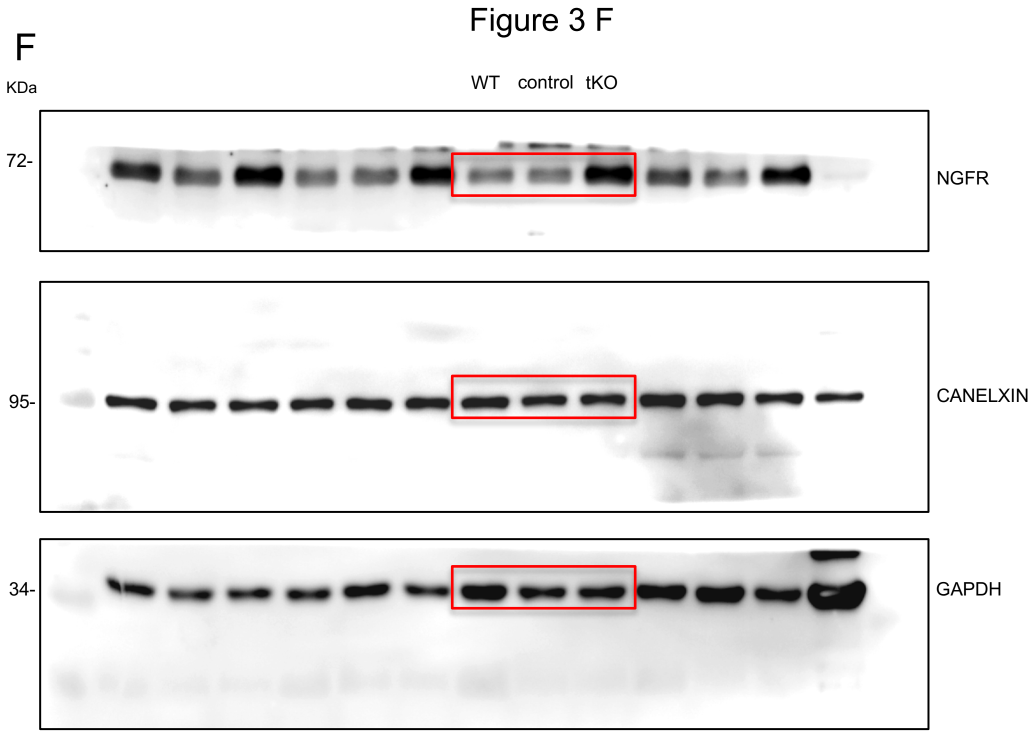
F
Figure 3 F
KDa
WT control tKO
72-
NGFR
95-
CANELXIN
34-
GAPDH

Figure 4

Figure 4 N

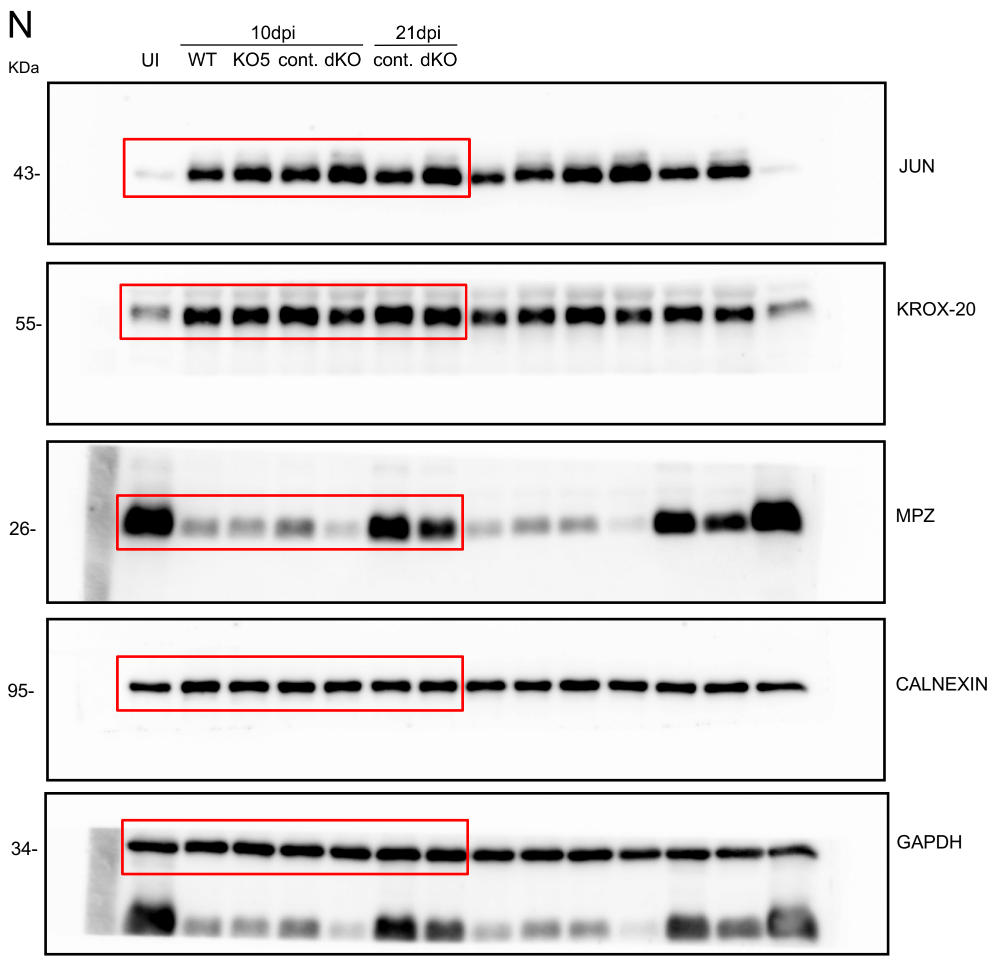

Figure 5

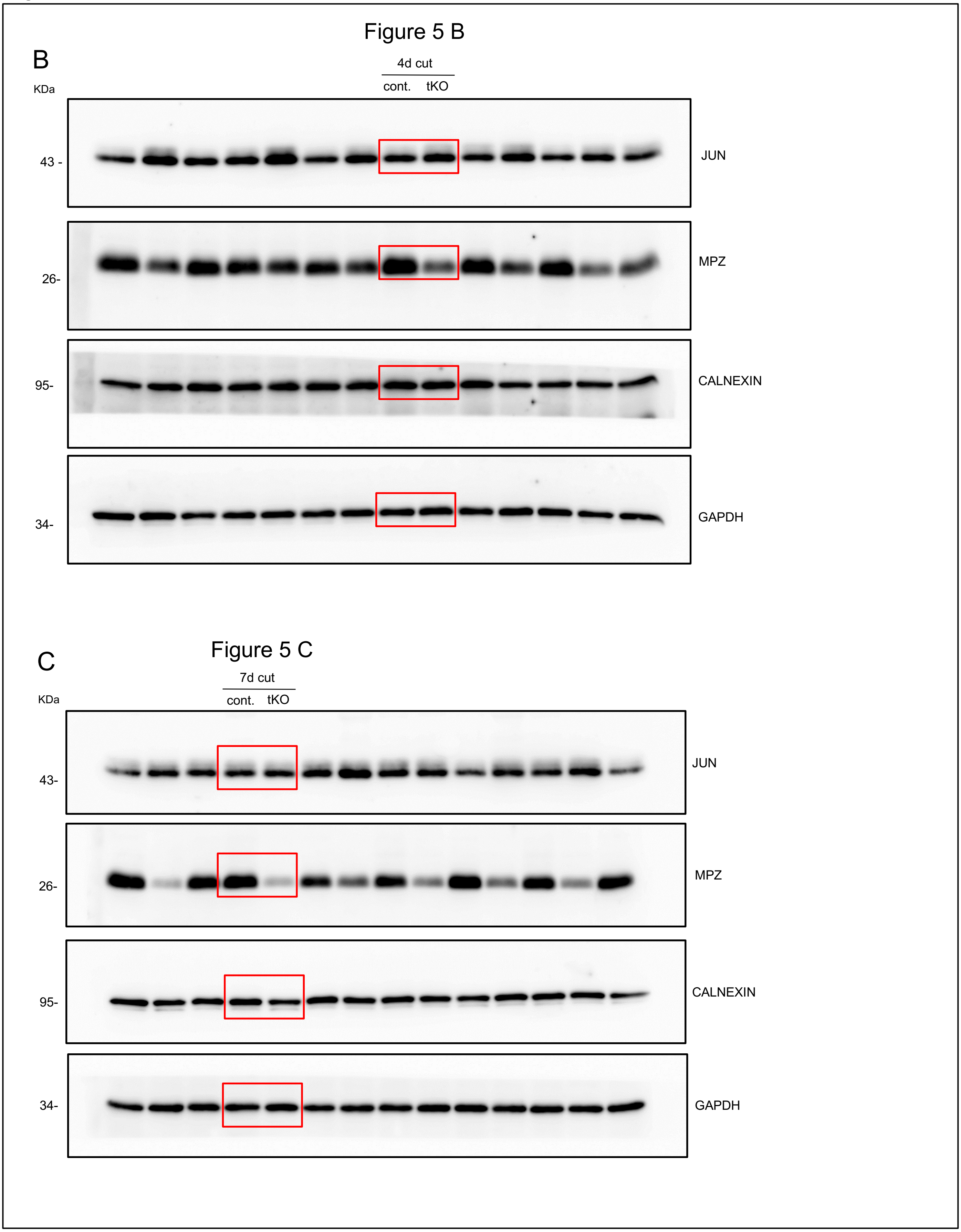
Figure 5 B
B
4d cut
cont.
tKO
KDa
43 -
JUN
26-
MPZ
95-
CALNEXIN
34-
GAPDH
Figure 5 C
C
7d cut
cont.
tKO
KDa
43-
JUN
26-
MPZ
95-
CALNEXIN
34-
GAPDH

Figure 5

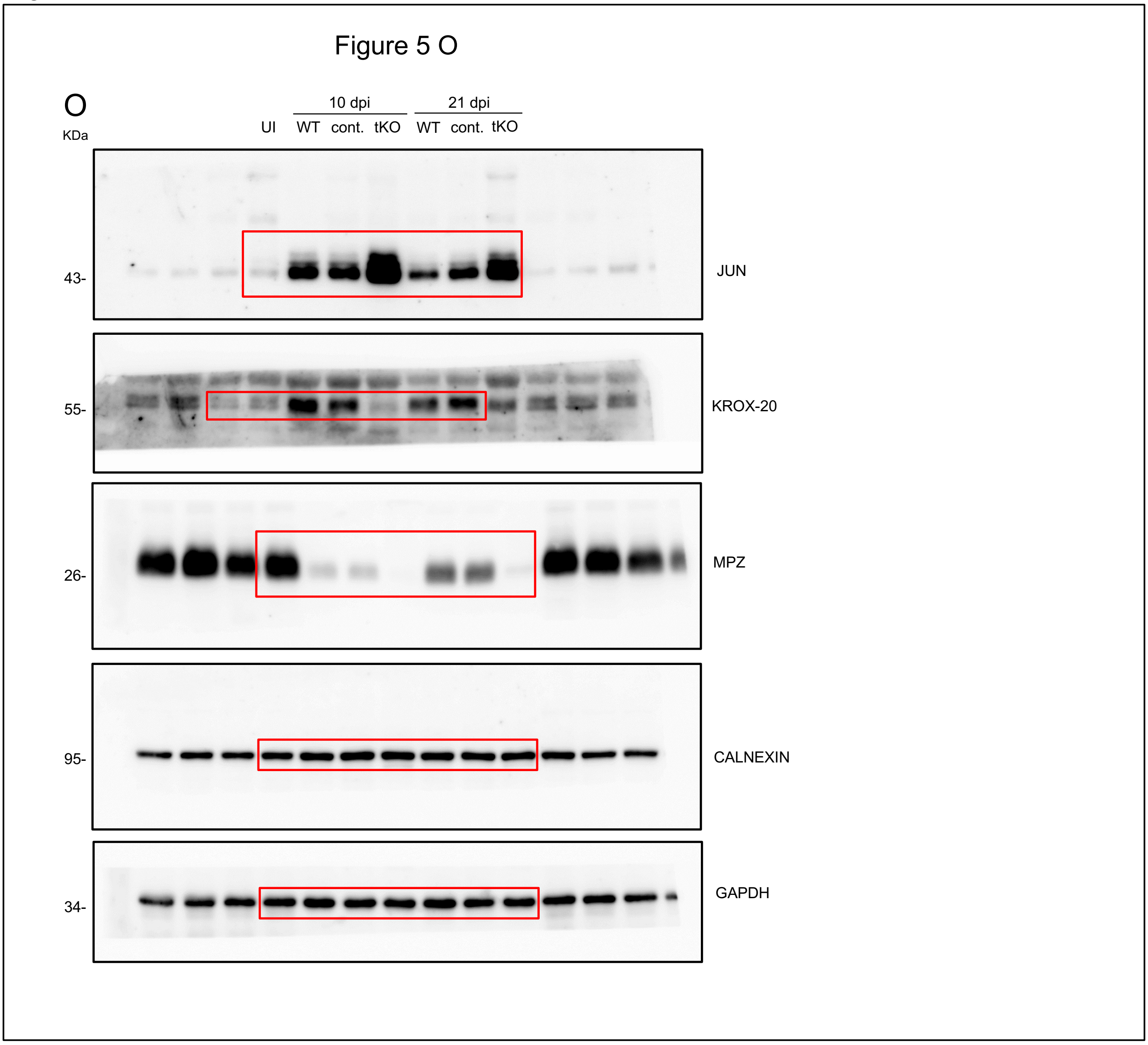
Figure 5 O
O
KDa
10 dpi
21 dpi
UI
WT
cont.
tKO
WT
cont.
tKO
43-
JUN
55-
KROX-20
26-
MPZ
95-
CALNEXIN
34-
GAPDH

Figure 9

Figure 9 H

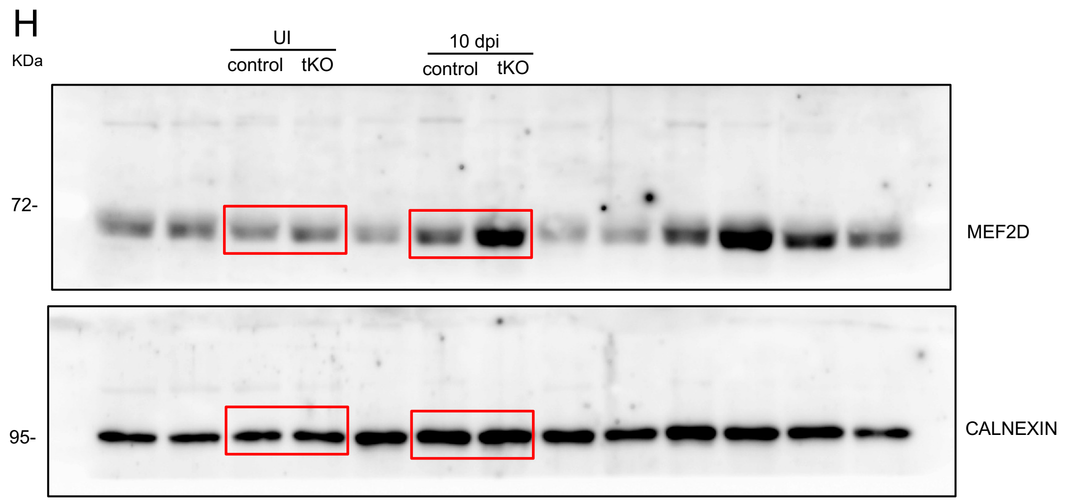

Figure 9 I

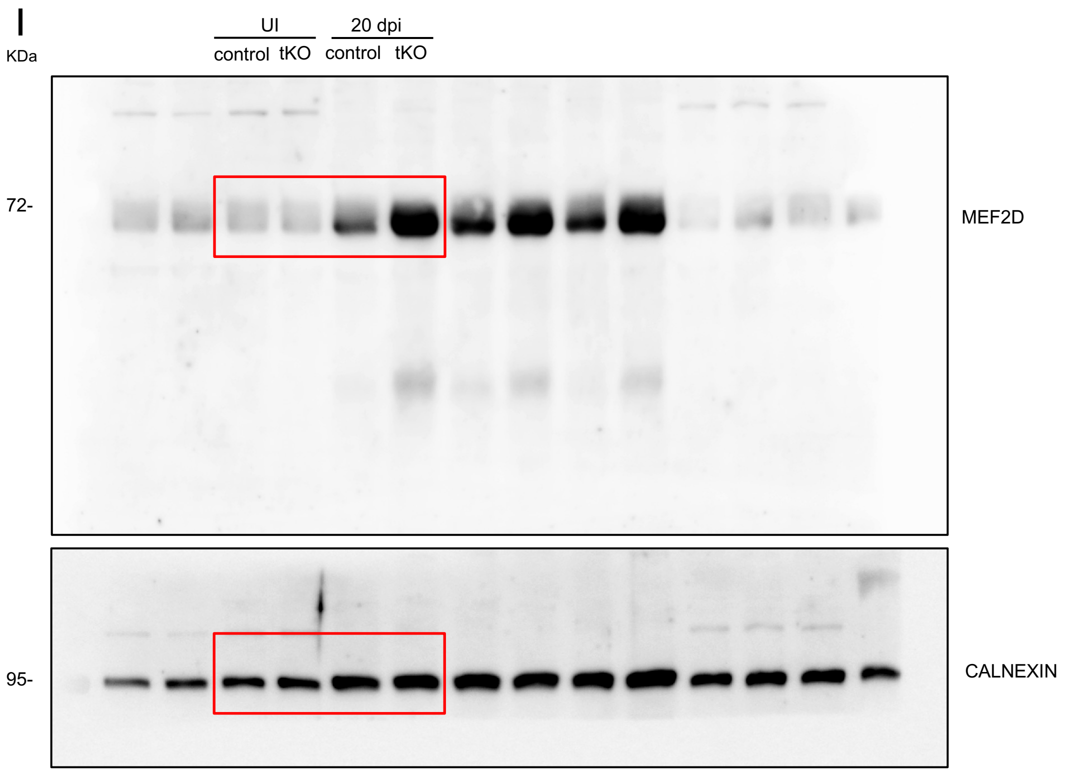

Figure 1 – figure supplement 1

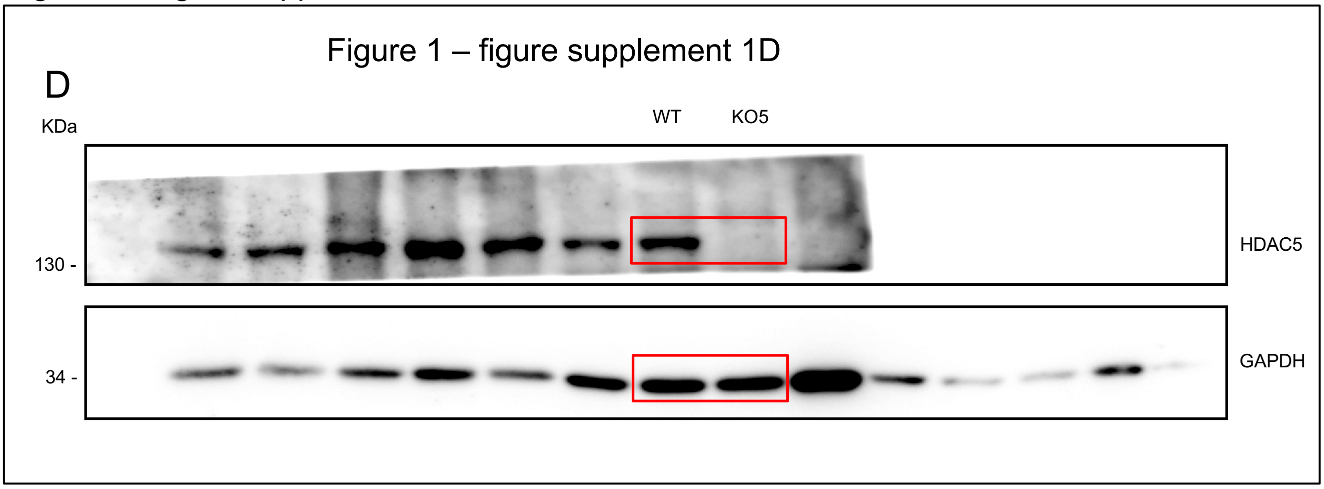

Figure 2 – figure supplement 1

Figure 2 – figure supplement 1C

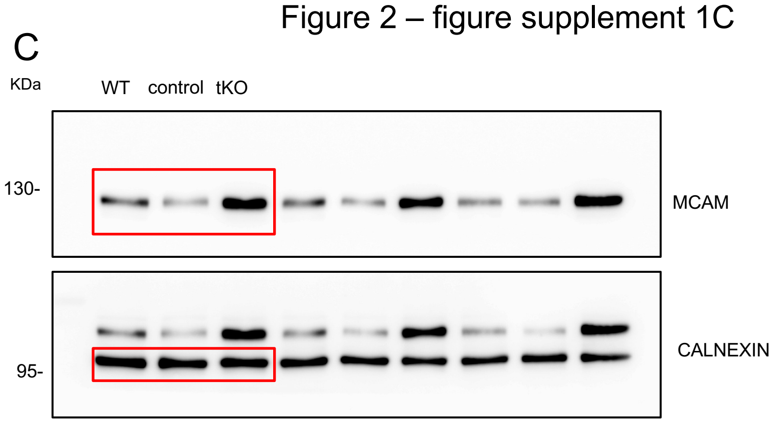

Figure 2 – figure supplement 1D

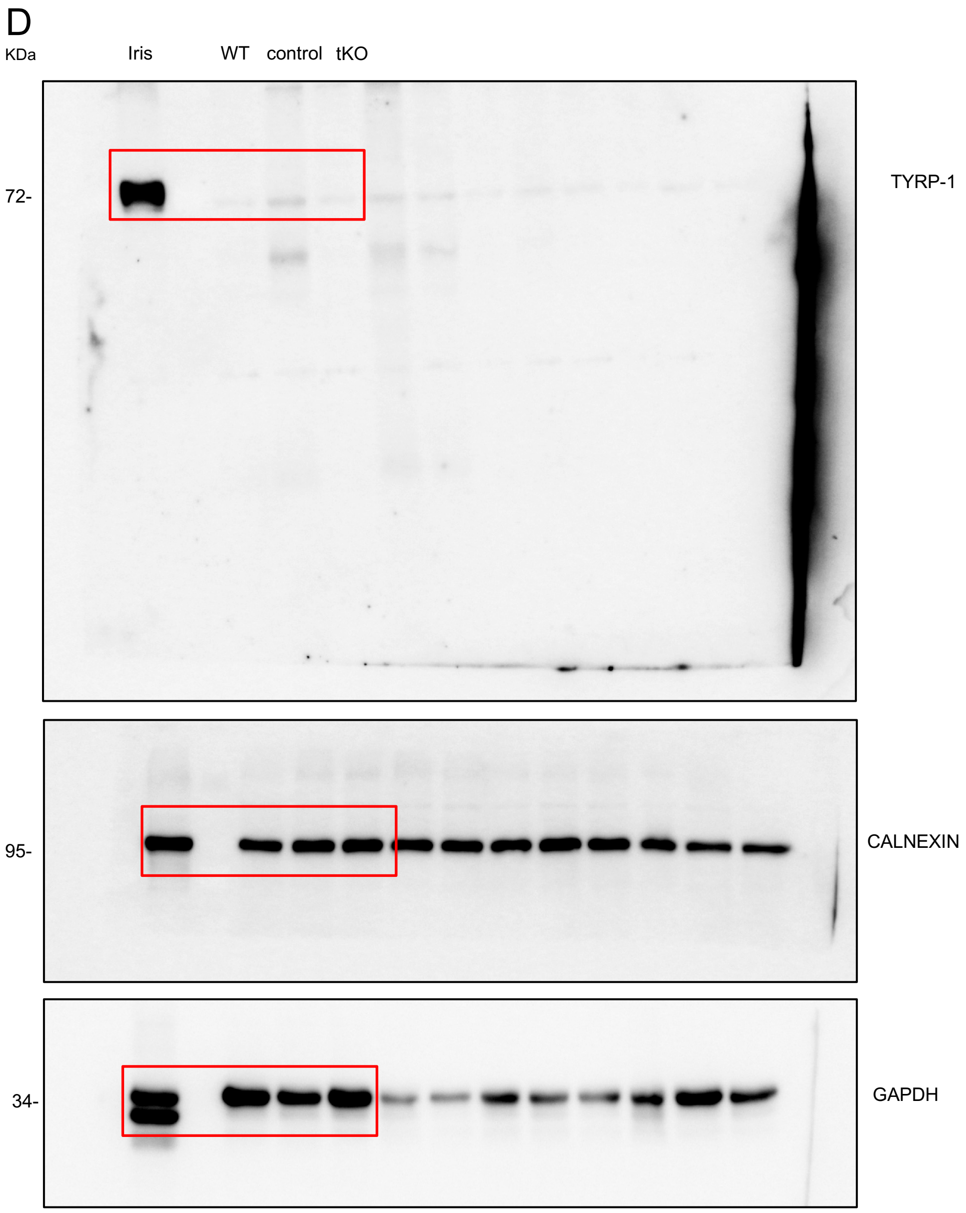

Figure 4 – figure supplement 1

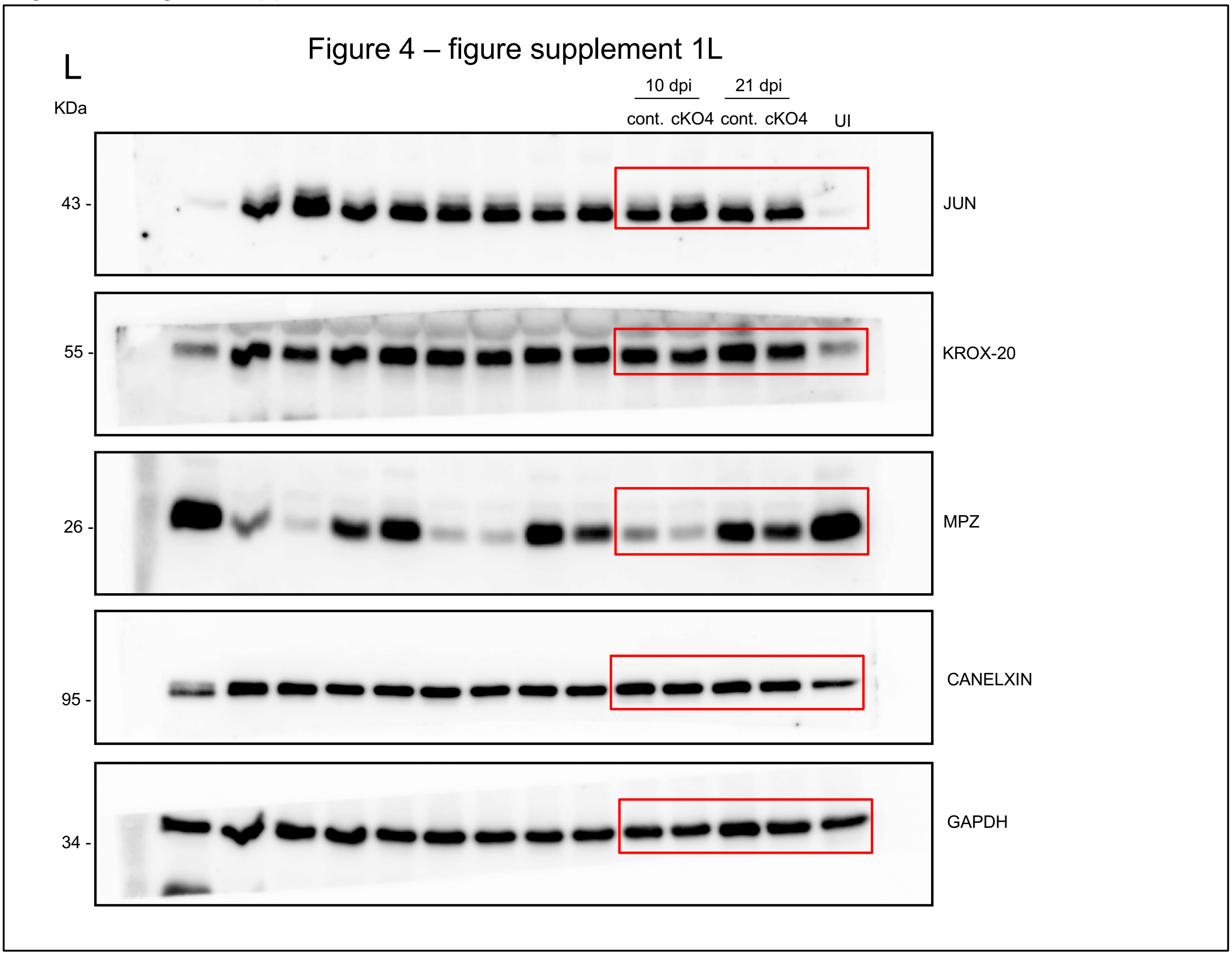

Figure 4 – figure supplement 3

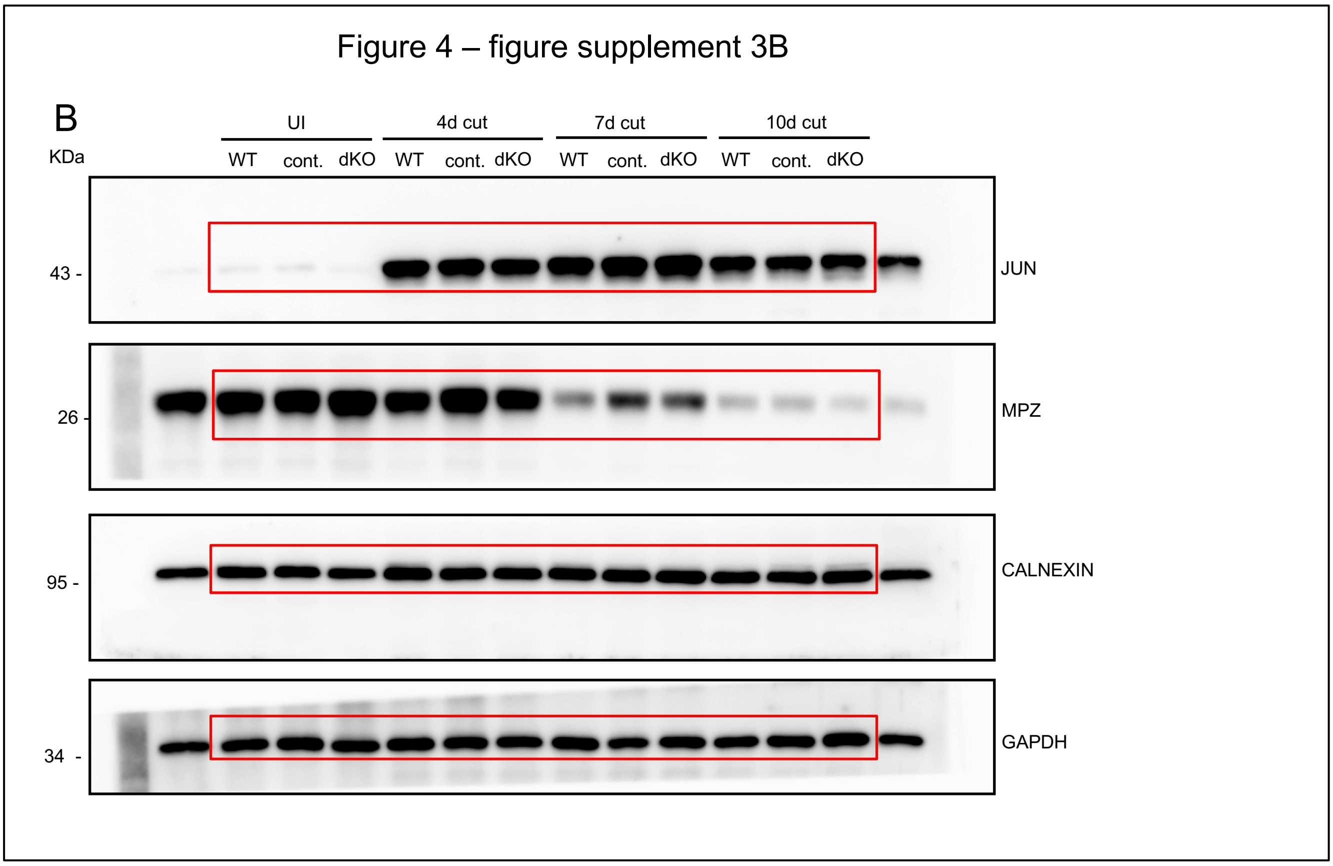

Figure 5 – figure supplement 1

Figure 5 – figure supplement 1L

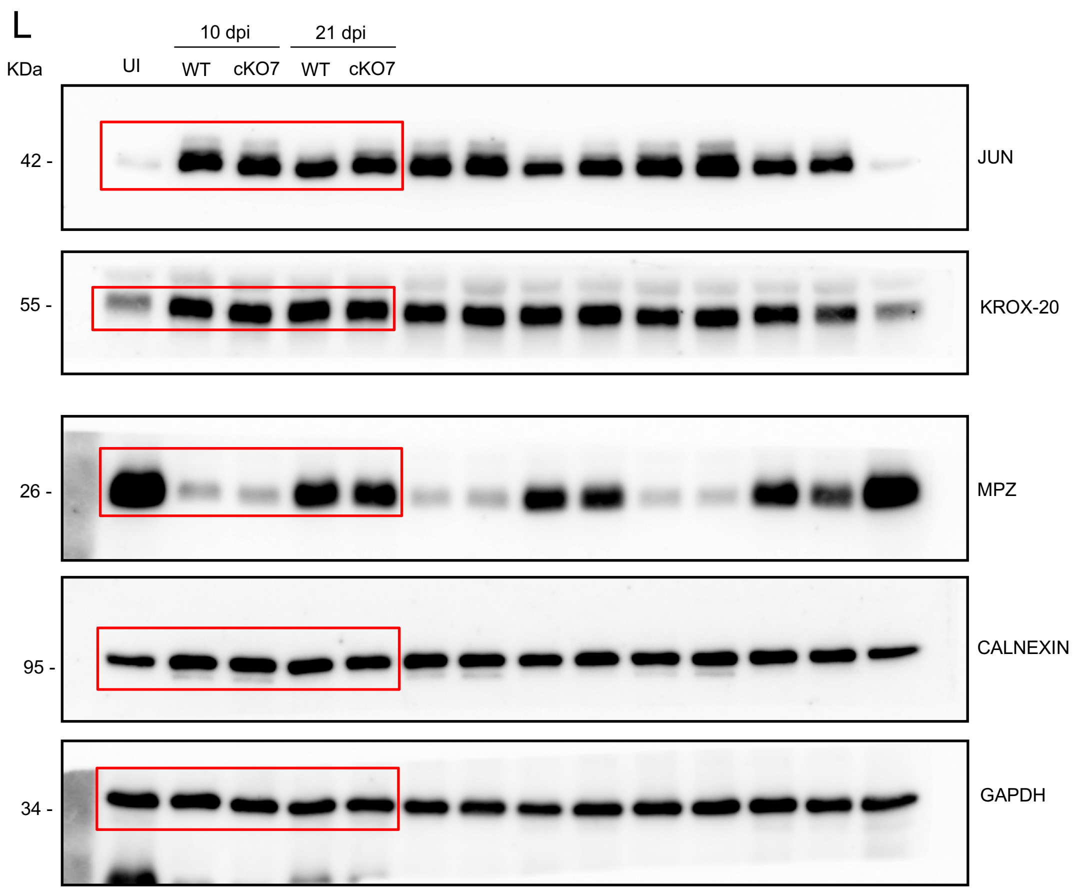

Figure 5 – figure supplement 2

Figure 5 – figure supplement 2H

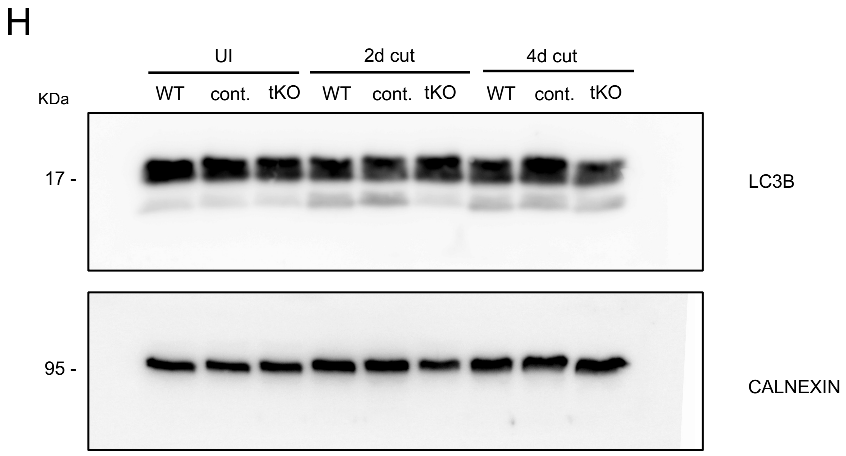

Supplement: Source data 4. [file elife-72917-data4.pdf]
